# Extending the Stochastic Titration CpHMD to CHARMM36m

**DOI:** 10.1021/acs.jpcb.2c04529

**Published:** 2022-10-03

**Authors:** João G. N. Sequeira, Filipe E. P. Rodrigues, Telmo G. D. Silva, Pedro B. P. S. Reis, Miguel Machuqueiro

**Affiliations:** BioISI - Instituto de Biossistemas e Ciências Integrativas, Faculdade de Ciências, Universidade de Lisboa, 1749-016 Lisboa, Portugal

## Abstract

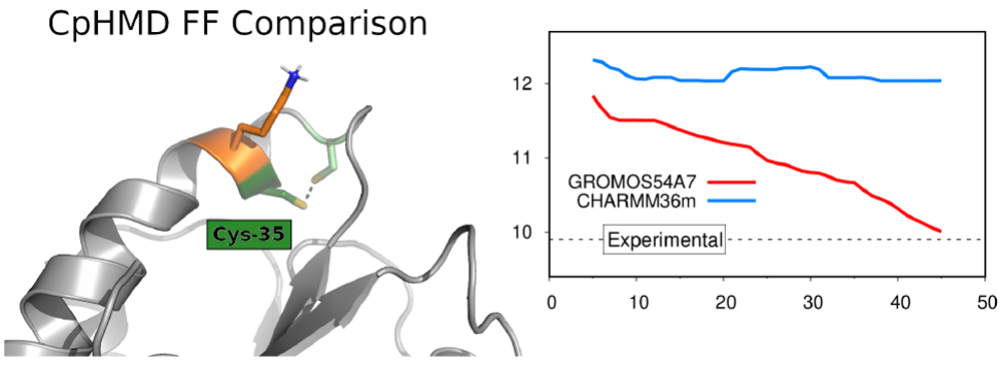

The impact of pH on proteins is significant but often neglected in molecular dynamics simulations. Constant-pH Molecular Dynamics (CpHMD) is the state-of-the-art methodology to deal with these effects. However, it still lacks widespread adoption by the scientific community. The stochastic titration CpHMD is one of such methods that, until now, only supported the GROMOS force field family. Here, we extend this method’s implementation to include the CHARMM36m force field available in the GROMACS software package. We test this new implementation with a diverse group of proteins, namely, lysozyme, Staphylococcal nuclease, and human and *E. coli* thioredoxins. All proteins were conformationally stable in the simulations, even at extreme pH values. The RMSE values (p*K*_a_ prediction vs experimental) obtained were very encouraging, in particular for lysozyme and human thioredoxin. We have also identified a few residues that challenged the CpHMD simulations, highlighting scenarios where the method still needs improvement independently of the force field. The CHARMM36m all-atom implementation was more computationally efficient when compared with the GROMOS 54A7, taking advantage of a shorter nonbonded interaction cutoff and a less frequent neighboring list update. The new extension will allow the study of pH effects in many systems for which this force field is particularly suited, i.e., proteins, membrane proteins, lipid bilayers, and nucleic acids.

## Introduction

The structure, stability, and function of proteins are usually pH-dependent. However, these pH effects are often ignored in molecular dynamics (MD) simulations due to the difficulty of sampling correct protonation states. Over the last 28 years, many constant-pH MD (CpHMD) methods have been developed to address these limitations.^1−44^ The different strategies employed can be distinguished mainly by (i) the type of protonation (continuous vs discrete); (ii) the force field (AMBER, CHARMM, GROMOS, OPLS, MARTINI, etc.) and its level of detail (all-atom, united-atom, and coarse grain); and (iii) the approximations used to deal with charge fluctuations in the simulation box, often related with the use of counterions and the long-range electrostatics treatment (reaction-field vs Ewald summation methods). In discrete CpHMD methods,^4,9,11,13−15,19,23,24,27−32,37−42,44^ continuum electrostatics calculations are used to estimate the energies for the Monte Carlo (MC) move while the MD simulations are run in either implicit or fully explicit solvent. The most recent continuous CpHMD methods are based on the λ-dynamics approach for free-energy calculations, where an individual λ variable is assigned to each titratable site of the protein.^8,16−18,20−22,25,26,33−36,43^ All protonation states coordinates vary continuously between 0 and 1, representing the protonated and deprotonated states, respectively.

With only a few exceptions,^15,36^ most methods are implemented in only one software package and for a single force field, hindering performance comparisons between methods and/or force fields. Nonetheless, there have been attempts to perform comparative studies of p*K*_a_ predictors, such as the p*K*_a_ Cooperative,^45,46^ that included several CpHMD methods. The blind predictions highlighted many problems with the available methods at the time. Similar initiatives should be promoted by the community to assess the evolution of the field in recent years.

Originally developed by Baptista et al.,^4^ the stochastic titration method is a seminal discrete protonation CpHMD. In this methodology, the MD simulation is periodically updated with protonation states sampled with MC from Poisson–Boltzmann-derived energies. All bonded and nonbonded terms are updated on-the-fly when a protonation/tautomer change occurs. Like the one maintained by Baptista et al., the implementation developed by our group only supported the GROMOS force field family, most notably, the 54A7.^47,48^ These are currently the two discrete CpHMD implementations that can be used directly with the GROMACS software package. Additionally to the large GROMOS community, the all-atom CHARMM36m is one of the most popular force fields, which is particularly suited for protein and lipids simulations.^49^ There are several CpHMD methods that already support the CHARMM force field family, most notable are the PHMD from the C. Brooks lab,^8,20,26^ the PHREM and EDS-HREX methods from the B. Brooks lab,^14,27^ the pH-REX and the PME-CpHMD methods from J. Shen lab,^18,21,33^ the hybrid neMD/MC in NAMD from the B. Roux lab,^34^ and the newly implemented CpHMD in GROMACS from the G. Groenhof lab.^43^ With this force field, the electrostatic interactions are treated using mostly the particle mesh Ewald (PME) approach,^50^ which requires system charge neutralization. This is a major limitation in its CpHMD implementation^51,52^ since the charge fluctuates as sites titrate during the simulation, complicating the neutralization effort. The simpler approach is to estimate the total charge of the solute at a given pH value and add a fixed amount of counterions, bringing the system charge fluctuations near zero.^11,42,53,54^ The resulting small and transient deviations from neutrality, can be handled by adding a uniform neutralizing background charge, which is implemented in all recent versions of the GROMACS software package,^55^ without triggering the appearance of unwanted artifacts.^56^ Other authors have developed specific methods to ensure complete charge neutrality, where titratable water molecules,^25^ individual ions,^21^ or ions as charge buffers^57,58^ are coupled to titratable sites, keeping the system neutral. Unfortunately, none of these charge titration methods are available yet in GROMACS. A combination of the two approaches would be ideal, where an average number of counterions generates a very realistic charge screening effect, while the water/ion titration ensures that the more approximate background charge correction is not triggered in the long-range electrostatics PME calculations.

Our stochastic titration CpHMD method derives all parameters required for the Poisson–Boltzmann (PB) calculations from the underlying force field. However, the treatment of model compounds retains some of the theoretical vagueness present in PB methods concerning their molecular definition and p*K*_a_ values.^15^ Instead of being a molecule featuring the same chemical group with a known experimental p*K*_a_ value, the model compound of a site is a nonphysical fragment defined as a portion of the amino acid residue (usually the complete side chain). The p*K*_a_ value of a model compound (p*K*^mod^) is then calibrated using experimental data of simple systems.^15^ For the amino acid side chains, we have used the p*K*_a_ values measured from NMR data on alanine-based pentapeptides (AAXAA, where X is a titratable residue).^59,60^ By performing a calibration, it is possible to offset systematic errors introduced by the PB parameters. Unfortunately, a new calibration procedure is required whenever important PB-related parameters are changed.

The systems used to benchmark new developments in the CpHMD field often include the same proteins.^5,8,9,15,18,19,28,36,37,61−63^ The hen egg-white lysozyme (HEWL) is arguably the most widely used test system for p*K*_a_ predictors^5,8,9,18,19,28,37,61−64^ due to the large number of residues with available experimental data,^65−67^ many of which with highly shifted p*K*_a_ values (mainly in the acidic range). Another important benchmark is the *Staphylococcus aureus* nuclease (SNase), which a very stable protein with a significant amount of experimental studies performed^68,69^ and several unexpectedly hard to predict residues. Unfortunately, both of these proteins lack titratable cysteine residues. In order to add this important residue to our benchmark, we have also included two thioredoxin proteins, the human form (^*h*^Trx) and another from *Escherichia coli* (^*Ec*^Trx), which has two reduced cysteine residues.

In this work, we extended our implementation of the stochastic CpHMD method to include the CHARMM36m (C^36m^) force field. Several procedures were optimized for this new force field, namely, the pH-dependent system charge neutralization and the calibration of the model compounds p*K*_a_ values. The results obtained with C^36m^ for the four protein systems were compared with the previous implementation, using GROMOS 54A7 (G^54A7^), and with experimental data.

## Methods

### CpHMD Extension to CHARMM36m

To extend the CpHMD implementation to another force field, it is necessary to create residue blocks for all titrable amino acid residues in the different protonation and tautomeric states.^62,70^ The C^36m^ force field already includes blocks of less common protonation states for some residues (the protonated aspartate and glutamate ASPP, GLUP; the deprotonated lysine LSN and cysteine CYM). For the Tyr residue, we adapted the phenolate block (PHEO) also available in the force field. The termini databases were updated to create all possible tautomeric neutral forms. From the atomic partial charges and the atom types (LJ parameters) of each block, we built the DelPhi databases of charges and radii. The radius of each atom was derived from its interaction energy with water at 2 RT, the same definition used in other CpHMD implementations using GROMOS force fields.^71^ However, the optimization of this energy cutoff may be required in the future to improve the performance of CpHMD with CHARMM force fields.

Another critical aspect to be considered in the C^36m^ implementation is the need to treat all long-range electrostatic interactions using PME, and consequent system charge neutralization.^52^ This is achieved by estimating the system’s total charge and adding the correct number of counterions to keep the charge fluctuations near zero.^54^ The remaining small system charge is counteracted by the PME background charge correction^56^ implemented in GROMACS. For a better comparison between force fields, we have adopted the same approach for the G^54A7^ simulations.

### System Setup

Four protein systems were prepared to test the C^36m^ implementation (Table 1): Lysozyme (HEWL),^72^*Staphylococcus aureus* nuclease (SNase),^73^ human thioredoxin (^*h*^Trx),^74^ and *E. coli* thioredoxin (^*Ec*^Trx).^75^ The experimental structure of SNase (PDB: 1STN) is missing 5 and 8 residues in the N- and C- termini, respectively, which were manually patched using PyMOL.^76^ In the ^*Ec*^Trx structure (PDB: 2TRX), the repeated chain (chain B, residues 1–108) was removed. All systems were solvated in a rhombic dodecahedral box with periodic boundary conditions.

**Table 1:** Proteins Used as Test Systems in This Study, Their PDB Codes, Number of Residues, and Number of Water Molecules in the Simulated System^a^

system name	protein name	PDB ID	# AAs	FF	# waters
HEWL	*G. gallus* lysozyme	4LZT	129	G^54A7^	6.4k
				C^36m^	6.0k
SNase	*S. aureus* nuclease	1STN	149	G^54A7^	8.5k
				C^36m^	8.0k
^*h*^Trx	*H. sapiens* thioredoxin	1TRW	105	G^54A7^	3.8k
				C^36m^	3.5k
^*Ec*^Trx	*E. coli* thioredoxin	2TRX	108	G^54A7^	3.9k
				C^36m^	3.4k

a The total number of ions added depends on the pH value and can be found in Table S1 in the Supporting Information. The number of water molecules in these systems will also change slightly since they are substituted by counterions.

The counterions used for each protein system and pH value (Table S1 in the Supporting Information) were calculated by evaluating the system’s average charge after short CpHMD segments (5 ns) in triplicate. The final number of counterions was selected so that the three replicates could achieve, on average, system neutrality. Although not used here, this counterion estimation step could have benefited from an initial estimation of the total charge using a computationally more efficient method, like PropKA,^77^ H++,^78^ DelPhiPKA,^79^ MCCE,^80^ or PypKa.^81^ Production runs were also done in triplicates and, after some time, some simulations converged their average system charges to values that differ more than 1 absolute protonic unit from the initial estimation. These simulations were restarted using a corrected number of counterions. This is a not very efficient solution since several pH values were restarted after the initial 5 ns. However, the adopted approach ensured that all charge fluctuations, which were dealt by the PME background charge correction, were very small, avoiding the appearance of unwanted artifacts.^56^

### Constant-pH MD Settings

A minimization procedure was applied to all systems. The steepest descent minimization algorithm was used with no constraints in the first step, while in the second the p-LINCS^82^ and SETTLE^83^ algorithms were turned on for solute and water molecules, respectively. Each replicate was initialized for 50 ps in NVT with an integration step of 1 fs, followed by another 50 ps in NPT with an integration step of 2 fs. In the NVT ensemble, the v-rescale thermostat^84^ was used to keep the temperature at 310 K (coupling constant of 0.05 ps). In the NPT ensemble, the v-rescale thermostat^84^ (coupling constant of 0.1 ps) was used in combination with the Parrinello–Rahman barostat^85^ (coupling constant of 0.5 ps and an isothermal compressibility of 4.5 × 10^–5^ bar^–1^).

In the CpHMD scheme, production MD simulations were interrupted at regular time intervals (τ = 20 ps) and new protonation states were obtained from Monte Carlo (MC) calculations using Poisson–Boltzmann (PB) derived free-energy terms.^4,11,37^ After the topology update, and prior to the next production MD segment, a very short (0.2 ps) solvent relaxation step (with frozen solute) is performed.^4,11^ All CpHMD simulations were performed using either the G^54A7^ or C^36m^ force fields for 50 ns. Three replicates were used to simulate the pH values ranging from 1–12 with a step of 1.0. The systems were considered to equilibrate within the initial 10 ns (see ”System Equilibration and Stability” section). Hence only the 10–50 ns segment was used for equilibrium analysis (see Results), except where stated otherwise. All MD simulations were performed with an integration step of 2 fs using GROMACS 5.1.5.^55^ The SPC and TIP3P water models were used in the G^54A7^ and C^36m^ simulations, respectively. The nonbonded interactions were treated with a single cutoff of 1.4/1.2 nm, updated every 5/10 steps in G^54A7^/C^36m^ simulations, respectively. Beyond the cutoff, all van der Waals interactions were truncated, and the Coulombic interactions were treated with the particle mesh Ewald (PME) method,^50^ using a Fourier space grid of 0.12 nm.

PB calculations were performed using DelPhi v5.0^86^ using a two focusing procedure.^87^ The dielectric constants were 80 and 2 for solvent and protein, respectively.^4,11^ The use of a dielectric constant of 2 for the protein is justified to mitigate the absence of polarization effects, not available in fixed charge models. Although the two force fields have slightly different charge distributions, we adopted the same value of solute dielectric constant to avoid adding any user bias. During the focusing procedure, a grid space of 0.1 nm was used for the larger grid, which was reduced to 0.025 nm in the smaller grid. Both grids contain 81 grid nodes on each side and are centered in the titrable group. The convergence threshold was set to 0.01 *k*_b_*T*/*e*.^88^ The MC runs were performed for 10^5^ cycles, using PETIT v1.6.1.^70^ In each cycle, random attempts are made to change the protonation state of every titrable group and of all pairs of sites with an interaction larger than 2 p*K* units.

### p*K*^mod^ Calibration

A model compound contains the same chemical group as a protonatable site in the protein whose p*K*_a_ value is presumably known.^15^ In our case, the model compounds are fragments of titratable amino acid residues, and their p*K*_a_ values (p*K*^mod^) require calibration. We have calibrated these p*K*^mod^ values for G^54A7^, and C^36m^ employing a previously reported protocol^15,32^ that uses experimental data of Alanine-based pentapeptides (Ala_2_-X-Ala_2_, where X is a pH titrating residue, i.e., Asp, Cys, Glu, His, Lys, or Tyr).^59,60^ These peptides are often used to study the effect of the protein environment on the protonation behavior of titrable residues.^15,32^ All N- and C-termini were capped with an acetyl or amino group, respectively, except for when these termini were titrating. In these cases, an Ala pentapeptide was used. The calibration procedure consisted of running CpHMD simulations (3 × 50 ns) at pH values near an initial p*K*^mod^ guess. After capturing the complete pH titration curve of the pentapeptide, a p*K*_a_ shift is obtained, which can be compared to the experimental p*K*_a_ values to generate the final p*K*^mod^ (Table S2 in the Supporting Information). The final values show some significant shifts that can be attributed to the model compound definition. In fact, the most sensitive sites are the termini, which are defined as quite simple chemical groups connected to the peptide main chain.

### Analyses and Error Calculations

Analyses were performed using GROMACS^55^ or in-house tools, visualized with PyMOL,^76^ and plotted with Gnuplot.^89^ Error values were calculated using the standard error of the mean from the 3 replicate simulations. In the case of p*K*_a_ values, we used a leave-one-out approach (jackknife) in the Hill curve fitting procedure to estimate the error values.^52^

## Results and Discussion

### System Equilibration and Stability

The main goal of this work is to evaluate the performance of the stochastic CpHMD method using the C^36m^ force field and comparing it with the G^54A7^ implementation. Four proteins commonly adopted in p*K*_a_ benchmarks (HEWL, SNase, ^*h*^Trx, and ^*Ec*^Trx) were used in this study. Before extracting equilibrium properties, like p*K*_a_ values, we need to ensure that our test systems are stable in our simulations at all pH values. We have calculated the Cα RMSD, radius of gyration, and secondary structure profiles over time for all replicates and pH values (pH values 4, 7, and 10 are shown in Figures S1–S3 in the Supporting Information). Overall, the simulations with the C^36m^ force field display similar structural properties variations when compared to the G^54A7^ simulations. We also report the cumulative average for the protonation of several residues (Glu-35 of HEWL and Glu-75 of SNase at pH 6, and Lys-36 of both Trx proteins at pH 11; Figure S4 in the Supporting Information) to illustrate a good convergence after 10 ns of simulation, even for the difficult cases and/or at the most extreme pH values.

**Figure 1:**
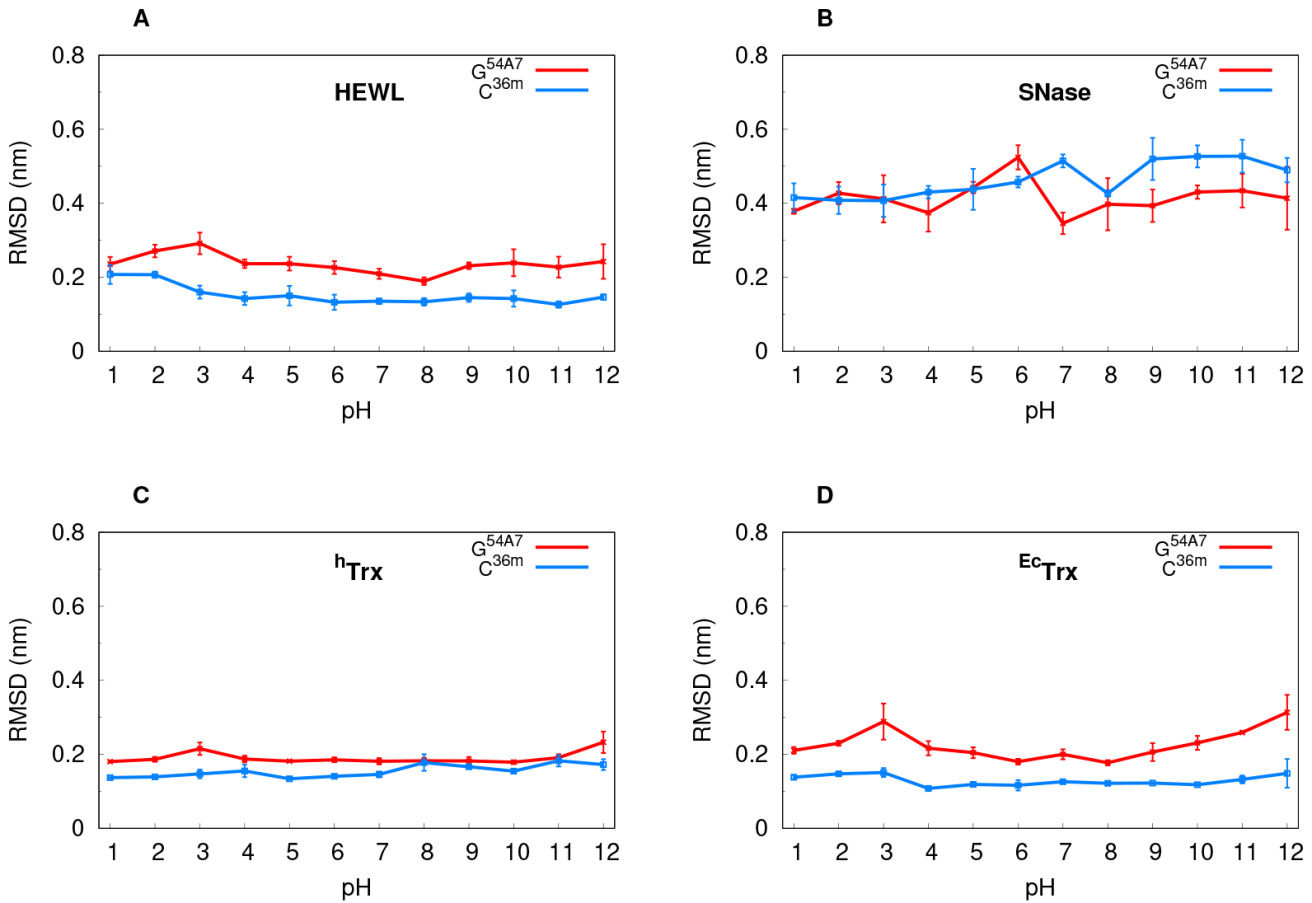
Average Cα RMSD values per pH value for each protein. The CpHMD simulations using G^54A7^ (red) and C^36m^ (blue) force fields are shown.

**Figure 2:**
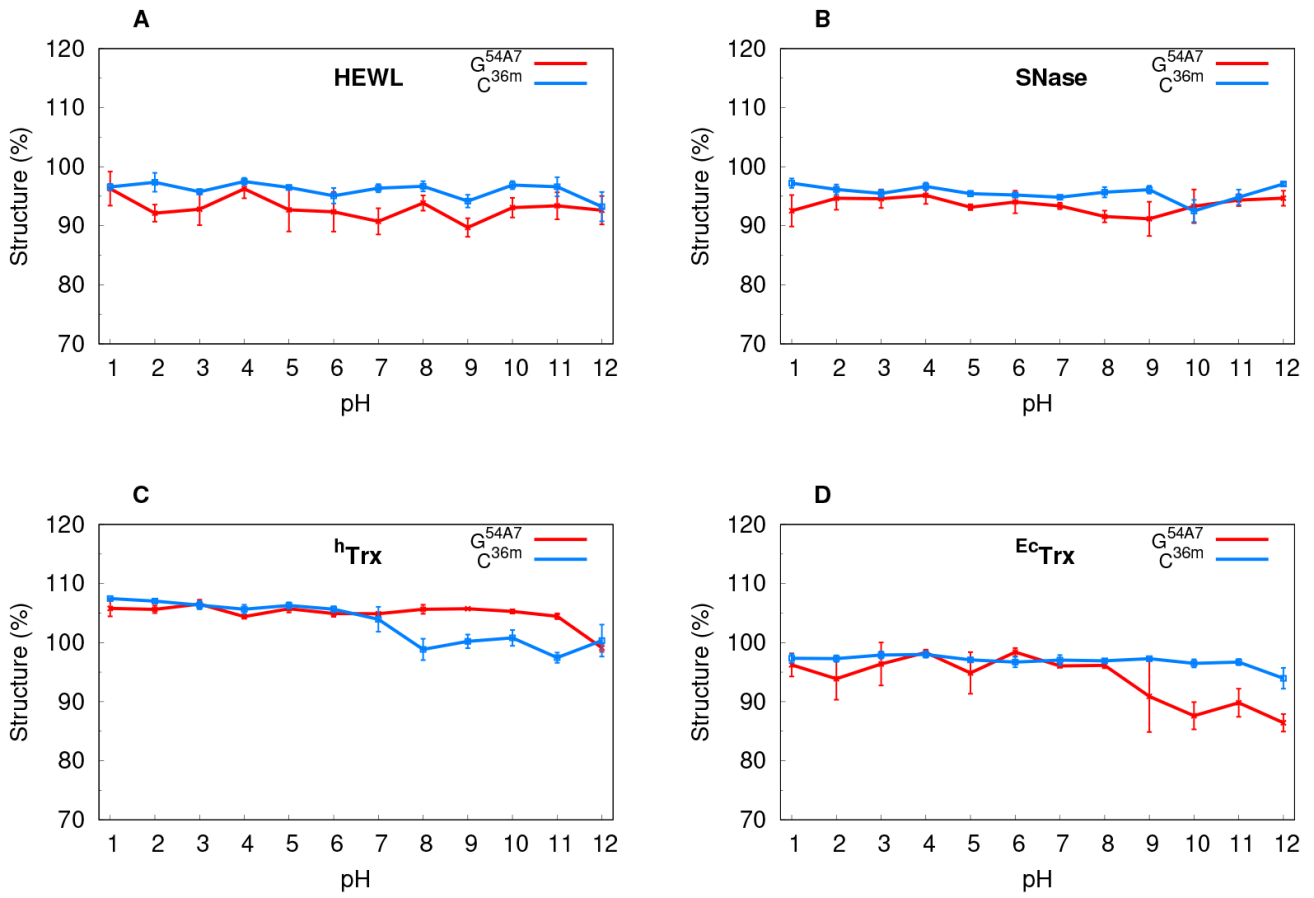
Average secondary structure per pH value for each protein. The CpHMD simulations using G^54A7^ (red) and C^36m^ (blue) force fields are shown. The structure represents the sum of all residues in helical or β-sheet conformations, and its percentages were calculated by comparing to the experimental structures. ^*h*^Trx shows a secondary structure percentage increase compared with its experimental reference, probably due to the absence of highly stabilized hydrogen bonds, like those present in the packed X-ray crystal structures.

Using the equilibrated segment (10–50 ns) of the simulations, we calculated the aforementioned structural properties and plotted them over our pH range (Figures 1, 2 and Figure S5 in the Supporting Information). The simulations with the C^36m^ force field usually have lower RMSD values while having similar gyration radii and secondary structure values. We observe that SNase has higher RMSD values than the other proteins (Figure 1B), indicating that its crystal structure may not be very representative in solution. In some cases, at more extreme pH values, we also see a slight increase in RMSD and a decrease in overall secondary structure (Figure 2C, D), suggesting some instability, but without jeopardizing the overall protein integrity and its protonation behavior. Interestingly, HEWL shows significant stability at acidic pH, even when using the G^54A7^ force field (Figure 1A). These results indicate that the use of an atomistic cutoff in combination with PME has a stabilizing effect on these simulations, contrary to what was observed for the group-based scheme and the generalized reaction-field treatment of the long-range electrostatic interactions.^15,37^ Altogether, C^36m^ tends to keep the conformations of the proteins closer to the initial structure when compared with G^54A7^, which can lead to differences in the conformational sampling and have an impact on each force field p*K*_a_ prediction ability.

### Total Titration Curves

**Figure 3:**
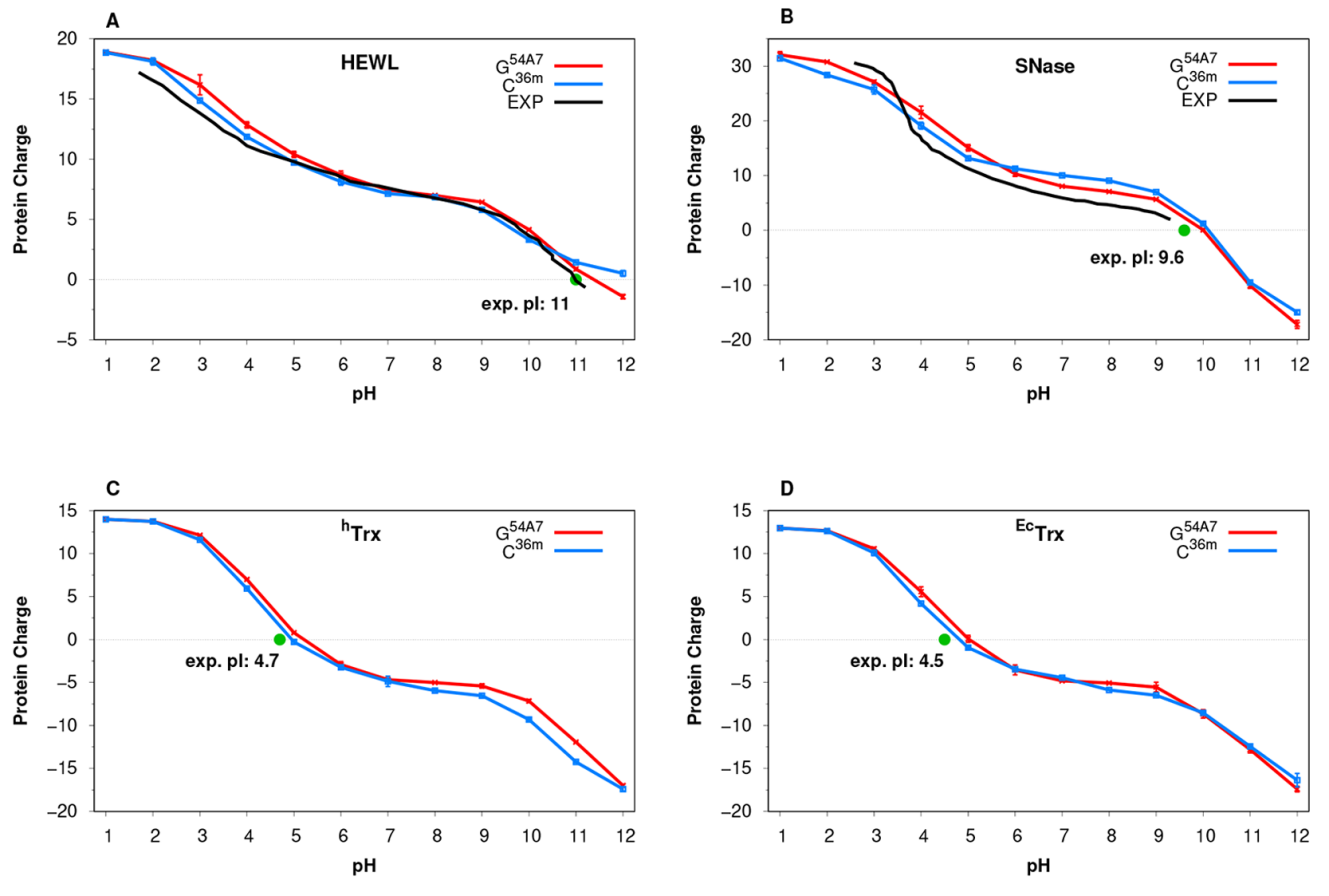
Full titration curves obtained for the four simulated systems using the two force fields. The available experimental titration curves are plotted as black lines, while the isoelectric points for each protein are represented with a green dot.^90−93^

From the CpHMD simulations, we calculated the total titration curves for each protein (Figure 3).

Overall, there is very good agreement between force fields and the available experimental data (for HEWL and SNase). Still, the SNase curve shows a slight deviation that is persistent across the pH range (Figure 3B). This discrepancy may be the result of a lack of representativeness of the crystallographic structure,^73^ consistent with the higher RMSD values observed (Figures 1B). This suggests that the protein may be trapped in an inadequate conformational region, which could be affecting our p*K*_a_ predictions. Both Trx proteins, for which there is no experimental total titration curve available, could also be validated around the protein charge neutrality using the experimental isoelectric point (pI) (Figure 3C,D and Table S3 the Supporting Information). Still, the good agreement shown between the titration curves and the pI values may be misleading since these properties tend to benefit from significant error cancellation, prompting for a more in-depth analysis.

### p*K*_a_ Predictions Benchmark

From the CpHMD simulations using C^36m^ and G^54A7^, we obtained individual p*K*_a_ values for all proteins studied (Tables S4–S7 and Figures S6–S9 in the Supporting Information). For some of these values, there are experimental data available,^94^ allowing for a direct comparison (Tables S8–S11 in the Supporting Information) and to benchmark the methods predictive ability (Table 2).

**Table 2:** RMSE of the p*K*_a_ Predictions Using the Null Model, PypKa, and the CpHMD Simulations with G^54A7^ and C^36m^ Force Fields, in Comparison to the Experimental Values (Tables S8–S11 in the Supporting Information)^a^

			G^54A7^		C^36m^		ΔFFs	
system	Null	PypKa	[0–10]	[10–50]	[0–10]	[10–50]	[0–10]	[10–50]
HEWL	1.27	0.71	0.90	1.00	0.96	0.99	0.67	0.68
SNase	0.76	1.25	1.24	1.22	1.53	1.46	0.66	0.72
^*h*^Trx	1.57	1.28	0.82	0.91	1.00	1.02	0.91	0.89
^*Ec*^Trx	1.69	2.83	2.08	1.52	2.29	2.14	0.68	0.83

a The mean average error (MAE) and maximum deviation values were also calculated (Table S12 in the Supporting Information). The RMSE between force fields (ΔFFs), where all titrating residues were considered (Tables S4–S7 in the Supporting Information), is also shown. In the CpHMD simulations, the conformational/protonation sampling was split in two time segments: [0–10] and [10–50] ns. The tyrosine residues were excluded from this calculation since their p*K*_a_ values were often outside our pH range.

The root-mean-square error (RMSE) metric captures the average deviation from the experimental values. Since the Null model RMSE is calculated using the water p*K*_a_ values of each residue type in the Ala-based pentapeptides, it can be used as the blind prediction or control independent of the proteins conformations. We also show the RMSE values calculated from the p*K*_a_ predictions using the PypKa software,^81,95^ a tool that relies on PB/MC calculations with a rigid structure that performs well when the structure is representative of the conformational ensemble in solution.^81^ We also split our CpHMD sampling into two time segments, one where there is still some significant conformational rearrangement ongoing (0–10 ns) and another where the proteins’ conformational ensemble should be better equilibrated (10–50 ns). Our results confirm that HEWL, despite its high Null model value, is very well-behaved, and all predictions are quite successful with a relatively low MAE (Table S12 in the Supporting Information). PypKa is able to outperform the more rigorous CpHMD method, suggesting a high representativeness in solution of the 4LZT crystal structure. The slight increase in RMSE in the 10–50 segment also supports the claim that the initial structure was particularly good for p*K*_a_ predictions. We do not see significant differences between force fields. The overall RMSE values obtained with HEWL are consistent with those reported in the literature^15,19,26,33,36,37^ using different methods and force fields (Table S13 in the Supporting Information), in particular when calculated with the same residues (Table S14 in the Supporting Information). The SNase protein has a particularly low Null model RMSE value (0.76), suggesting an easier challenge. However, both the PypKa and the CpHMD predictions are modest, indicating that the crystal structure has several residues in environments dissimilar to those exhibited during the experimental p*K*_a_ measurements. Moreover, these results suggest that the experimental structure contains interactions that are not very likely to occur and that could not be reverted within 50 ns of CpHMD simulations. We noted that the SNase structure used in this work (PDB code: 1STN) is different from the one adopted by other groups in their CpHMD benchmarks (PDB code: 3BDC),^33,34,36^ which may also explain the slightly worse performance when comparing with those studies (Table S15 in the Supporting Information). The C^36m^ predictions were slightly worse, indicating that CpHMD simulations using this force field tend to struggle more when an initial strong conformational bias needs to be corrected. In ^*h*^Trx, the CpHMD predictions were very successful using both force fields, improving on the Null model, the rigid structure calculations, and the previous reported data from the literature (Table S16 in the Supporting Information).^36^ In contrast with the human form, the ^*Ec*^Trx has two reduced cysteine residues.^96^ However, in the 2TRX crystal structure these residues were kept oxidized. Consequently, using a method that relies solely on the initial structure, such as PypKa, results in very poor p*K*_a_ estimations. In these cases, the structural equilibration allowed by CpHMD methods is essential to improve the predictions. The conformational relaxation is more pronounced in the simulations using the G^54A7^ force field compared to C^36m^, as was observed for the SNase protein.

We calculated the RMSE values and the mean average error for each type of titrating residue and observed that it is easier to predict p*K*_a_ values for some residues than others (Table 3 and Table S17 in the Supporting Information).

**Table 3:** RMSE Values Per Force Field for Each Titrating Residue Type^a^

		RMSE (ME)			
residue	exp/total residues	PypKa	G^54A7^	C^36m^	ΔFFs
CTr	1/4	0.78 (−0.78)	0.66 (+0.66)	0.63 (+0.63)	0.28 (+0.24)
Asp	20/33	1.38 (−0.47)	1.10 (+0.58)	1.09 (+0.18)	0.88 (+0.34)
Glu	24/29	0.74 (−0.41)	1.10 (+0.57)	0.62 (+0.14)	0.67 (+0.36)
Cys	2/2	4.43 (+4.02)	1.93 (+1.75)	3.69 (+3.48)	1.78 (−1.73)
His	6/7	1.95 (−0.40)	1.23 (−0.94)	2.40 (−1.78)	1.28 (+0.86)
NTr	2/4	0.86 (+0.33)	1.44 (−1.42)	1.38 (−1.07)	1.02 (−0.60)
Lys	6/51	0.52 (+0.19)	0.58 (−0.32)	1.01 (−-0.87)	0.62 (+0.34)

a The mean error (ME) values can be seen as a measure of the bias and are shown inside parentheses. The MAE and the maximum deviation values were also calculated (Table S17 of Supporting Information). The RMSE between force fields (ΔFFs) is also shown. The tyrosine residues were excluded from this calculation since their p*K*_a_ values were often outside our pH range.

As expected, the residue types that are usually located on the protein surface, hence well-solvated, are usually charged at physiological pH (CTr, Asp, Glu, NTr, and Lys) and will have their p*K*_a_ values closer to the reference, leading to lower RMSE values. Contrarily, His and Cys residues tend to be more internalized and in their neutral form, which presents a stronger challenge for p*K*_a_ calculations. Finally, the Tyr residues showed a similar trend but were excluded from this study since their p*K*_a_ values are often extrapolated (>12) and have large error values associated. By calculating the mean error of each residue type, we also observe a consistent bias in both force fields, where anionic groups have positive shifts in the p*K*_a_ values, whereas cationic groups have negative ones. This happens due to an overstabilization of the residues neutral form, and can be related with either the force field parameters (the groups interactions with SPC/TIP3P water) or the PB model and the atomic radii adopted. There is also a trend in the bias (ME) of the PypKa results (Table 3), where all residue types tend to favor the protonation state more abundant in the crystal. This leads to charged Asp, Glu, Ctr, Ntr, and Lys, while Cys and His are more commonly found in their neutral form. Our p*K*_a_ predictions for the two cysteine residues are particularly poor, but there are other residues where our methodology also struggles. The C^36m^ simulations show a very high RMSE value for histidines, while the G^54A7^ performs worse with glutamic acids. These differences between force fields may be the result of just one or two bad predictions, very specific to a particular protein and should be studied individually in more detail.

### Challenging Cases

We identified four exceptionally challenging cases in which the titrating groups have significantly shifted p*K*_a_ values measured experimentally or are trapped in nonrepresentative conformations: Glu-35 in HEWL; Glu-75 and His-121 of SNase; Asp-26 of ^*h*^Trx; Cys-32 and Cys-35 in ^*Ec*^Trx.

The Glu-35 is a critical residue in HEWL with which rigid body p*K*_a_ calculations struggle.^97^ The typical increase of the dielectric constant of the protein in PB-based predictors leads to better overall results by improving the predictions of the water-facing residues while worsening the estimations of the less abundant catalytic sites. This residue is located in the catalytic cleft of the protein and has a shift of ∼2 p*K* units in its experimental p*K*_a_ (6.2). Unlike PypKa, both force fields predicted correctly this residue (6.02 ± 0.17 for G^54A7^ and 5.99 ± 0.32 for C^36m^) highlighting the usefulness of CpHMD simulations when no drastic conformational rearrangements are coupled with protonation.

**Figure 4:**
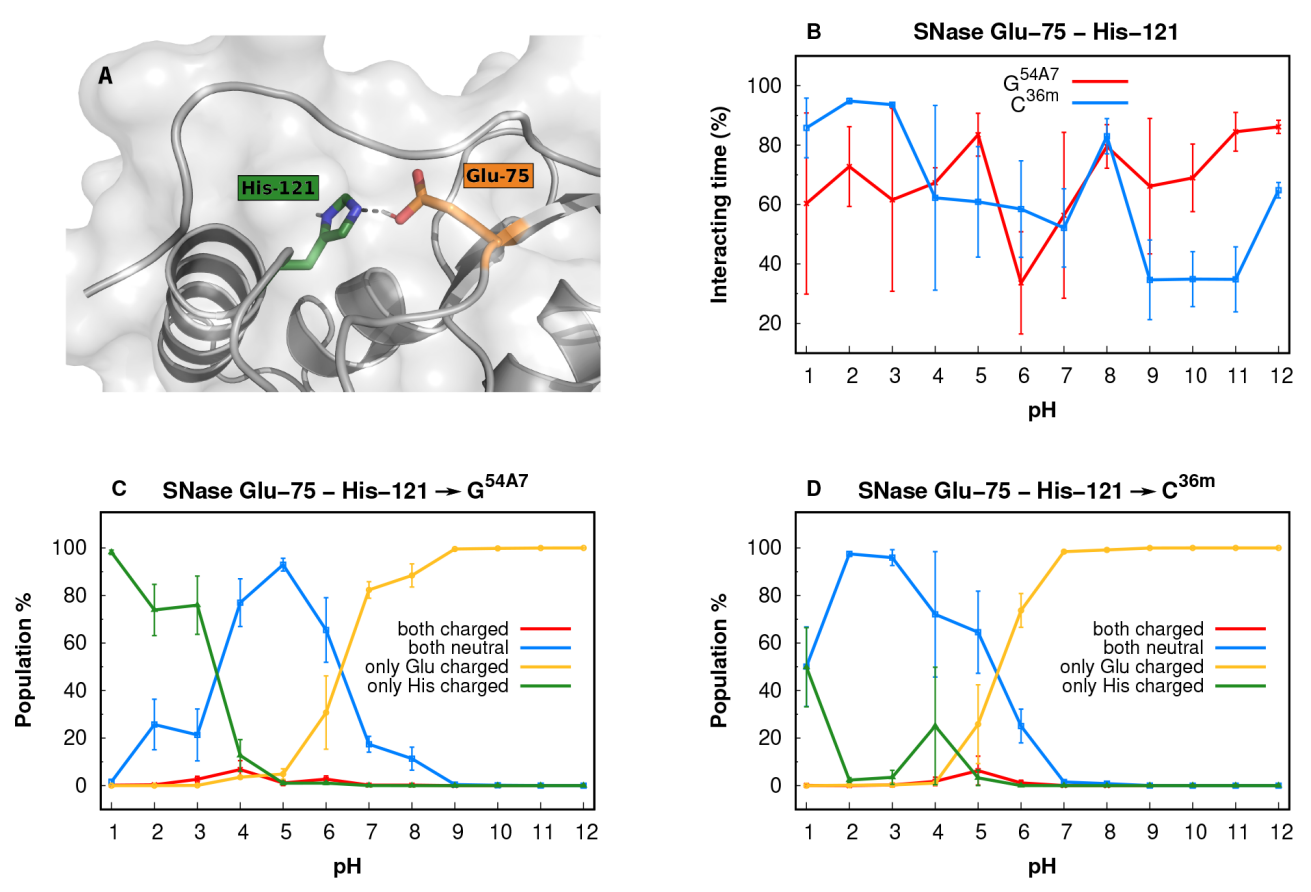
SNase Glu-75 and His-121 case study. (A) Structural representation of the SNase pocket (light gray) where Glu-75 (orange) is interacting with His-121 (dark green). (B) Interaction time (%) between Glu-75 and His-121, over the simulated pH values. The interactions were computed using a 0.4 nm cutoff distance between the residues side chains. (C) G^54A7^ population distribution of protonation state combinations for Glu-75 and His-121, across the simulated pH values. (D) C^36m^ population distribution of protonation state combinations for Glu-75 and His-121, across the simulated pH values.

Residues Glu-75 and His-121 of SNase are quite buried (Figure 4A) and interact with each other at all pH values (Figure 4B). At pH 3.5–5.0, this pair of anionic-cationic residues is either stabilized by a salt bridge (charged interaction) or a hydrogen bond (neutral interaction). The relative energies between these two states depend mostly on force field parameters and the group’s solvent exposure. Indeed, in a well-solvated environment, the salt bridge is favored, while desolvation stabilizes the hydrogen bond. The experimental p*K*_a_ values for both residues (3.26 for Glu-75 and 5.30 for His121; Table S9 in the Supporting Information) indicate that in solution at pH 3.5–5.0, the two residues form either a salt bridge or induce a local structural rearrangement that allows for both residues to be well-solvated and charged. In our G^54A7^ CpHMD simulations, no such conformational transition is observed in this pH range, leading to an overstabilization of the hydrogen bond between the two neutral forms (Figure 4C) and an inversion of the p*K*_a_ values (6.32 for Glu-75 and 3.40 for His-121; Table S9 in the Supporting Information). This lack of conformational transition is also observed in the C^36m^ simulations (5.41 for Glu-75 and 1.00 for His-121; Table S9 in the Supporting Information), where the overstabilization of the histidine neutral form is even more pronounced (Figure 4D), leading to a larger deviation in this residue and contributing significantly to the poor performance of this force field with this residue type (Table 3).

**Figure 5:**
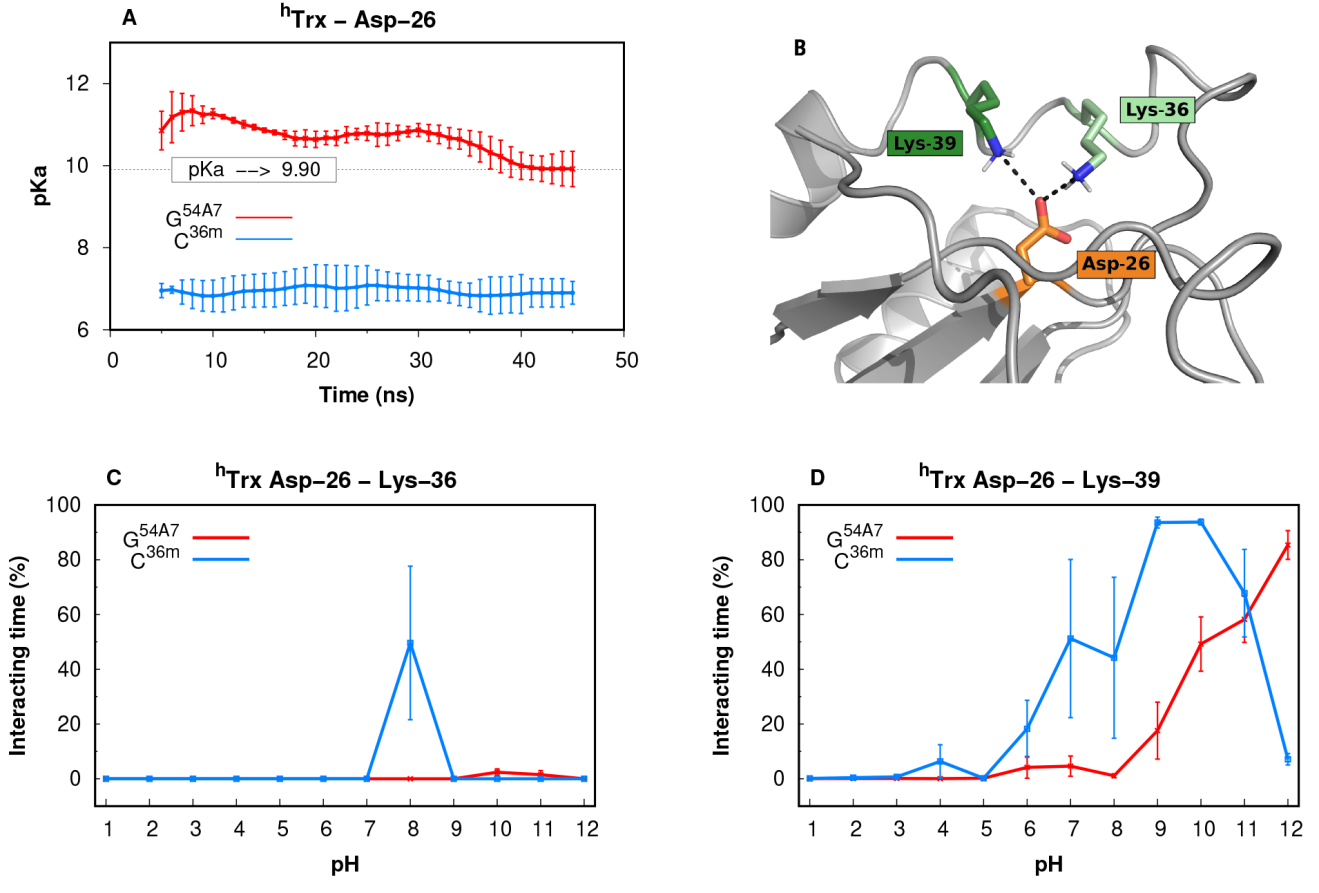
^*h*^Trx Asp-26 case study. (A) p*K*_a_ values of Asp-26 for both force fields calculated using the sampling of only 10 ns sliding windows. (B) Structural representation of the ^*Ec*^Trx pocket (light gray) where Asp-26 (orange) is located and interacting with Lys-36 (light green) and Lys-39 (dark green). The snapshot was obtained from one of the pH 8.0 simulations. Interaction times (%) between Asp-26 and Lys-36 (C) or Lys-39 (D), over the simulated pH values. The interactions were computed using a 0.4 nm cutoff distance between the residues side chains.

The ^*h*^Trx Asp-26 residue has one of the most shifted p*K*_a_ values (9.9) ever measured in a wild-type protein.^98^ For an Asp residue to have such a high p*K*_a_ value, its neutral form needs to be considerably stabilized by shielding it from the solvent and/or the charged state needs to be destabilized by a persistent interaction with an anionic group. In ^*h*^Trx, the Asp-26 residue is quite buried, and its p*K*_a_ shift probably results from the desolvation effect. We observe a clear difference in our ability to accurately estimate the p*K*_a_ value of this residue using G^54A7^ or C^36m^ (Figure 5A). While in GROMOS, the estimated p*K*_a_ converges to the experimental value with time, in CHARMM, the p*K*_a_ is always underestimated. We identified two interactions with neighboring lysine residues (Lys-36 and Lys-39; Figure 5B-D) that can explain this lowering in the p*K*_a_ value. These ionic interactions are slightly more prevalent in the C^36m^ simulations in the 6–10 pH range, which overstabilizes the Asp-26 ionized form.

**Figure 6:**
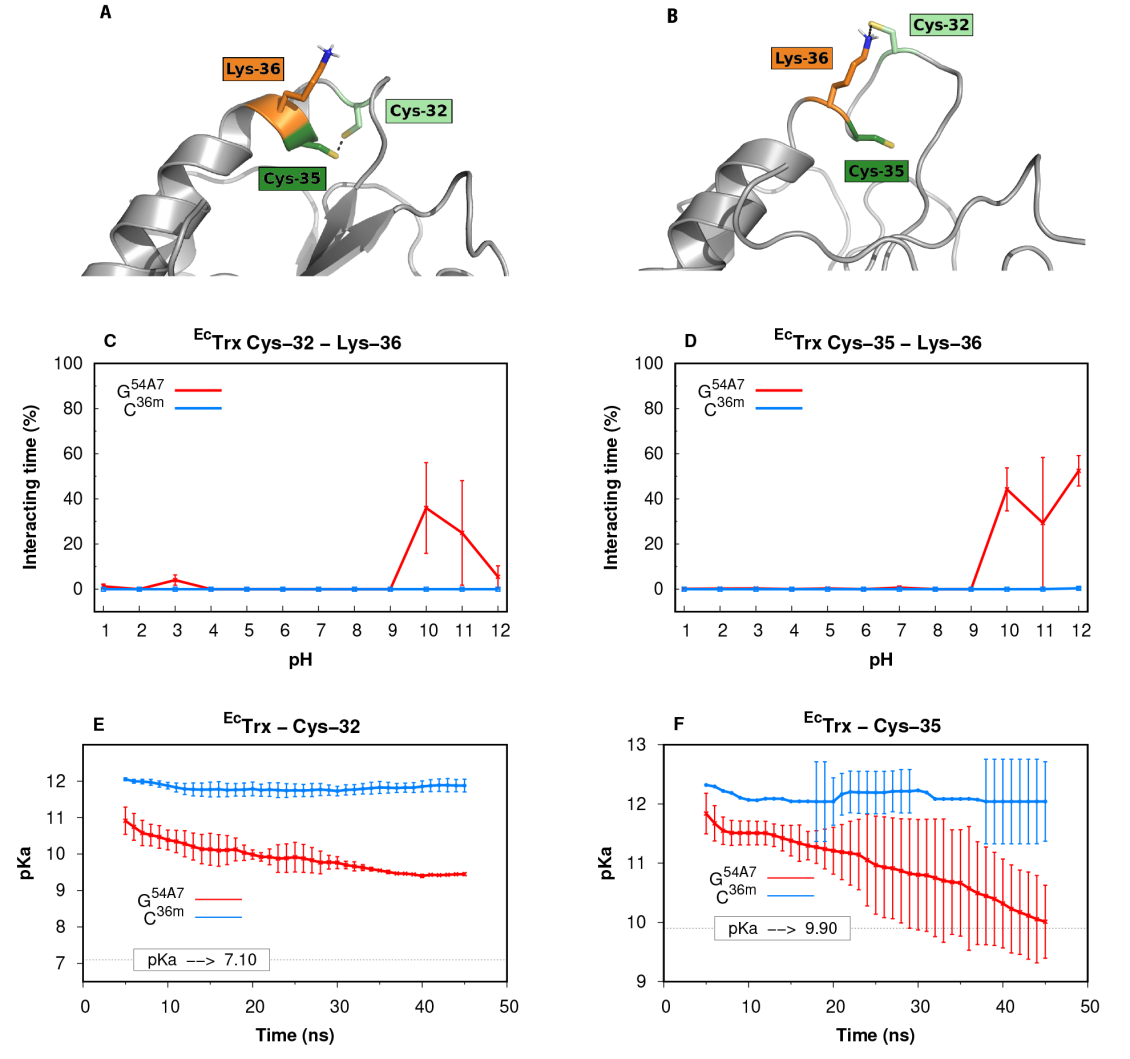
PH titration of Cys-32 and Cys-35 in ^*Ec*^Trx. (A) Starting structure of ^*Ec*^Trx (light gray) CpHMD simulations, depicting Cys-32 (light green) and Cys-35 (dark green) in close proximity, with Lys-36 (in orange) solvent exposed. (B) Selected snapshot highlighting an ionic pair between Cys-32 and Lys-36, while Cys-35 remained only partially exposed to solvent. Interaction times (%) between Lys-36 and Cys-32 (C) or Cys-35 (D), over the simulated pH values. The interactions were computed using a 0.4 nm cutoff distance between the residues side chains. p*K*_a_ values of Cys-32 (E) or Cys-35 (F) for both force fields calculated using the sampling of only 10 ns sliding windows. The errors bars were omitted for the pH values where the jackknife combinations failed the Hill curve fit.

The ^*Ec*^Trx protein was a particularly interesting test case due to the presence of two titrating cysteine residues (Cys-32 with a p*K*_a_ value of 7.1 and Cys-35 with a p*K*_a_ value of 9.9). Since these two residues were oxidized in the X-ray structure, they start in close proximity in our simulations (Figure 6A). From the experimental p*K*_a_ values, it is expected that, at least at pH values over 9.9, the two cysteine residues should break apart (Figure 6B) to accommodate the two negative charges. Furthermore, the p*K*_a_ value of Cys-32 (7.1) is significantly lower than that of a well-solvated cysteine (8.55^59^), indicating that this residue needs to interact with a neighboring cationic residue. Indeed, in the G^54A7^ simulations, both Cys-32 and Cys-35 are interacting with Lys-36 (Figure 6C, D) at pH values where both residues are ionized. However, in the C^36m^ simulations, there is no interaction with Lys-36 and little ionization in these two residues. This difference in behavior has a strong impact on the p*K*_a_ values calculated in the CpHMD simulations (Figure 6E, F). The conformational transition is coupled with the p*K*_a_ value of cysteines captured by the G^54A7^ simulations over time. Contrarily, with C^36m^, the p*K*_a_ values of both cysteine residues are consistently >12. During the 50 ns long G^54A7^ simulations, we observe a conformational change that resulted in an accurate prediction of the p*K*_a_ of Cys-35. However, for the Cys-32 residue, the observed conformational transition appears to be insufficient for a good estimation of its p*K*_a_ value. Significantly enriching conformations where Cys-32 (and not Cys-35) interacts with a cationic group (Figure 6B), necessary to obtain a p*K*_a_ value of 7.1, is a real challenge for any force field. The description of titrating groups that are originally in the neutral form and require a structural reorganization to stabilize the ionized form is particularly poor in the C^36m^. It is not possible to know which force field, if any, correctly describes the kinetics of the protonation-coupled side-chain rearrangement. Here, we can only discuss whether a force field provides within tens or hundreds of nanoseconds the conformational changes essential to accurately predict protein p*K*_a_ values. The slow side chain kinetics of C^36m^ have already been independently identified by other groups using a different CpHMD method.^43^ These authors propose a selective reduction in the torsion barriers of side chains in the force field and showed that it significantly improved their p*K*_a_ predictions without affecting the sampling of the overall configurational space accessible to proteins. We plan to confirm these findings usingour method, expecting that such an approach will help mitigate many of the shortcomings associated with C^36m^ as evidenced in this work.

### Computational Efficiency

The computational efficiency of the stochastic titration CpHMD method using the two force fields was evaluated systematically in a controlled benchmark (Table 4). We used HEWL and SNase, which have a different number of residues and titrable residues. As expected, the results show that the simulations run faster with the smaller protein (HEWL). Interestingly, we observe that the CpHMD simulations using the all-atom C^36m^ force field are faster than the united-atom G^54A7^, which has a significantly smaller number of particles in the system. This can be explained by the long-range electrostatics cutoff and corresponding update interval. With CHARMM, it is common to use a cutoff of 1.2 nm, and the energies are updated every 10 steps. In contrast, with GROMOS, a 1.4 nm cutoff and an update interval of 5 steps are used. The larger cutoff and higher update frequency in G^54A7^ increases the number of direct interactions and the time it takes to update those neighboring lists, which cancels out the computational gain of having fewer particles in the system. Nevertheless, the higher number of particles in the protein when using C^36m^ still impacts the percentage of time spent in the PB/MC step since the electrostatic potential in the PB-solver grid seems to be slightly harder to converge. We also note that the solvent relaxation step is very small and only takes 1–2% of the total simulation time. Therefore, it seems that, to improve the overall computational efficiency of CpHMD, we need to move the MD part to GPU code (13-fold improvement with HEWL using GROMACS 2021.5 CUDA code with a NVIDIA GeForce RTX 3080) and find ways to optimize or substitute the PB energies calculations (MC is almost instantaneous in CpHMD) without loosing accuracy.^99^

**Table 4:** Simulation Speed Benchmark (ns/day) for the G^54A7^ and C^36m^ Force Fields^a^

						MD	
system	total residues (N)	titrable residues (N)	FF	CpHMD (ns/day)	PB/MC	relax	production
HEWL	129	21	G^54A7^	15.7 ± 0.1	19.6%	1.7%	77.4%
			C^36m^	17.5 ± 0.1	24.5%	2.1%	72.2%
SNase	149	56	G^54A7^	10.4 ± 0.0	34.3%	1.1%	63.4%
			C^36m^	10.7 ± 0.1	40.1%	1.6%	57.2%

a The percentage of time spent in each step (PB/MC, MD relax, and MD production) is also described. HEWL and SNase were selected since they are the two larger proteins studied. The benchmark was performed on 8 cores of a 16/32-cores/threads machine equipped with two Intel Xeon CPU E5-2620 v4 @ 2.10 GHz processors.

## Conclusions

Until now, our implementation of the stochastic CpHMD method only supported the GROMOS force field family. Here, we introduced an extension to the method that allows for CpHMD simulations using the C^36m^ force field. We used a diverse group of proteins, HEWL, SNase, ^*h*^Trx, and ^*Ec*^Trx, some of which are often used in benchmark assays, that allowed us to benchmark the p*K*_a_ predictive abilities of both force fields. We showed that all proteins were conformationally stable in the simulations, even at extreme pH values. We also observed that, in some systems, like HEWL, the p*K*_a_ predictions become slightly worse with the simulation time, indicating that the initial structure is very representative but also suggesting that the protein structure may be starting to become unstable at some pH values.^37^ Notwithstanding, the RMSE values obtained with HEWL are in line with those reported in the literature using different methods and force fields.^15,19,36,37^ The SNase proved to be a very difficult challenge with very modest CpHMD predictions that were unable to beat the Null model. It is not surprising that this protein and several of its mutations were adopted by one of the first p*K*_a_ challenges in the “Protein Electrostatics” community.^45,46^ In ^*h*^Trx, the CpHMD predictions were very successful using both force fields. However, the two reduced cysteine residues that required a structural rearrangement in ^*Ec*^Trx, were very hard to sample correctly, leading to bad p*K*_a_ predictions particularly using C^36m^. This issue may be related to the torsion barriers of the amino acid side chains being too high in C^36m^ force field, which was shown to be detrimental to p*K*_a_ predictions.^43^ In overall, these force field differences lead to noticeable discrepancies in performance where the CHARMM36m method proved to be less accurate in some residues than its GROMOS counterpart.

In terms of computational efficiency, we noticed that, unexpectedly, the all-atom C^36m^ implementation was slightly faster than the G^54A7^ one, as a result of the longer cutoff for the nonbonded interactions and a higher update frequency used with the all-atom force field. We expect that this C^36m^ extension will allow the study of pH effects in many systems for which this force field is particularly suited, i.e., proteins, membrane proteins, lipid bilayers, and nucleic acids. Furthermore, since the CHARMM force field family has great development and support, we hope that this upgrade is ever-evolving and will allow us to directly compare our implementation of the stochastic CpHMD method with other known CpHMD implementations that support C^36m^.

## Supporting Information Available

Tables showing the number of counterions used, p*K*^mod^ values for both force fields, isoelectric points of all proteins, individual p*K*_a_ values obtained from the calculations, and RMSE/MAE/Max; deviation values obtained from the literature; plots showing time series of RMSD, radius of gyration, secondary structure, and individual titration curves of all sites calculated with both force fields (PDF)
All force field files and the CpHMD code used in this work (ZIP)

## References

[ref1] Mertz J. E.; Pettitt B. M. Molecular Dynamics at a Constant pH. Int. J. High Perform. Comput. Appl. 1994, 8, 47–53.

[ref2] Baptista A. M.; Martel P. J.; Petersen S. B. Simulation of protein conformational freedom as a function of pH: constant-pH molecular dynamics using implicit titration. Proteins Struct. Funct. Bioinf. 1997, 27, 523–544. 10.1002/(SICI)1097-0134(199704)27:4<523::AID-PROT6>3.0.CO;2-B. 9141133

[ref3] Börjesson U.; Hünenberger P. H. Explicit-solvent molecular dynamics simulation at constant pH: methodology and application to small amines. J. Chem. Phys. 2001, 114, 9706 10.1063/1.1370959.

[ref4] Baptista A. M.; Teixeira V. H.; Soares C. M. Constant-pH molecular dynamics using stochastic titration. J. Chem. Phys. 2002, 117, 4184–4200. 10.1063/1.1497164.

[ref5] Burgi R.; Kollman P. A.; van Gunsteren W. F. Simulating proteins at constant pH: An approach combining molecular dynamics and Monte Carlo simulation. Proteins Struct. Funct. Bioinf. 2002, 47, 469–480. 10.1002/prot.10046. 12001225

[ref6] Dlugosz M.; Antosiewicz J. M. Constant-pH molecular dynamics simulations: a test case of succinic acid. Chem. Phys. 2004, 302, 161–170. 10.1016/j.chemphys.2004.03.031.

[ref7] Dlugosz M.; Antosiewicz J. M.; Robertson A. D. Constant-pH molecular dynamics study of protonation-structure relationship in a heptapeptide derived from ovomucoid third domain. Phys. Rev. E 2004, 69, 021915 10.1103/PhysRevE.69.021915. 14995499

[ref8] Lee M. S.; Salsbury F. R.; Brooks C. L. III Constant-pH molecular dynamics using continuous titration coordinates. Proteins Struct. Funct. Bioinf. 2004, 56, 738–752. 10.1002/prot.20128. 15281127

[ref9] Mongan J.; Case D. A.; McCammon J. A. Constant pH molecular dynamics in generalized Born implicit solvent. J. Comput. Chem. 2004, 25, 2038–2048. 10.1002/jcc.20139. 15481090

[ref10] Khandogin J.; Brooks C. L. III Constant pH molecular dynamics with proton tautomerism. Biophys. J. 2005, 89, 141–157. 10.1529/biophysj.105.061341. 15863480 PMC1366513

[ref11] Machuqueiro M.; Baptista A. M. Constant-pH Molecular Dynamics with Ionic Strength Effects: Protonation-Conformation Coupling in Decalysine. J. Phys. Chem. B 2006, 110, 2927–2933. 10.1021/jp056456q. 16471903

[ref12] Stern H. A. Molecular simulation with variable protonation states at constant pH. J. Chem. Phys. 2007, 126, 164112 10.1063/1.2731781. 17477594

[ref13] Machuqueiro M.; Baptista A. M. Molecular Dynamics Constant-pH and Reduction Potential: Application to Cytochrome c_3_. J. Am. Chem. Soc. 2009, 131, 12586–12594. 10.1021/ja808463e. 19685871

[ref14] Itoh S. G.; Damjanović A.; Brooks B. R. pH replica-exchange method based on discrete protonation states. Proteins Struct. Funct. Bioinf. 2011, 79, 3420–3436. 10.1002/prot.23176. PMC3373023 22002801

[ref15] Machuqueiro M.; Baptista A. M. Is the prediction of p*K*_a_values by constant-pH molecular dynamics being hindered by inherited problems?. Proteins Struct. Funct. Bioinf. 2011, 79, 3437–3447. 10.1002/prot.23115. 22072522

[ref16] Vorobjev Y. N. Potential of mean force of water-proton bath and molecular dynamic simulation of proteins at constant pH. J. Comput. Chem. 2012, 33, 832–842. 10.1002/jcc.22909. 22278814

[ref17] Donnini S.; Tegeler F.; Groenhof G.; Grubmüller H. Constant pH molecular dynamics in explicit solvent with λ-dynamics. J. Chem. Theory Comput. 2011, 7, 1962–1978. 10.1021/ct200061r. 21687785 PMC3114466

[ref18] Wallace J. A.; Shen J. K. Continuous constant pH molecular dynamics in explicit solvent with pH-based replica exchange. J. Chem. Theory Comput. 2011, 7, 2617–2629. 10.1021/ct200146j. 26606635 PMC6425487

[ref19] Swails J. M.; Roitberg A. E. Enhancing conformation and protonation state sampling of hen egg white lysozyme using pH replica exchange molecular dynamics. J. Chem. Theory Comput. 2012, 8, 4393–4404. 10.1021/ct300512h. 26605601

[ref20] Goh G. B.; Knight J. L.; Brooks C. L. Constant pH molecular dynamics simulations of nucleic acids in explicit solvent. J. Chem. Theory Comput. 2012, 8, 36–46. 10.1021/ct2006314. 22337595 PMC3277849

[ref21] Wallace J. A.; Shen J. K. Charge-leveling and proper treatment of long-range electrostatics in all-atom molecular dynamics at constant pH. J. Chem. Phys. 2012, 137, 184105 10.1063/1.4766352. 23163362 PMC3511335

[ref22] Bennett W. F. D.; Chen A. W.; Donnini S.; Groenhof G.; Tieleman D. P. Constant pH simulations with the coarse-grained MARTINI model - Application to oleic acid aggregates. Can. J. Chem. 2013, 91, 839–846. 10.1139/cjc-2013-0010.

[ref23] Henriques J.; Costa P. J.; Calhorda M. J.; Machuqueiro M. Charge Parametrization of the DvH-c_3_ Heme Group: Validation Using Constant-(pH,*E*) Molecular Dynamics Simulations. J. Phys. Chem. B 2013, 117, 70–82. 10.1021/jp3082134. 23199023

[ref24] Carvalheda C. A.; Campos S. R. R.; Machuqueiro M.; Baptista A. M. Structural effects of pH and deacylation on surfactant protein C in an organic solvent mixture: a constant-pH MD study. J. Chem. Inf. Model. 2013, 53, 2979–2989. 10.1021/ci400479c. 24106805

[ref25] Chen W.; Wallace J. A.; Yue Z.; Shen J. K. Introducing titratable water to all-atom molecular dynamics at constant pH. Biophys. J. 2013, 105, L15–L17. 10.1016/j.bpj.2013.06.036. 23972860 PMC3752133

[ref26] Goh G. B.; Hulbert B. S.; Zhou H.; Brooks C. L. III Constant pH molecular dynamics of proteins in explicit solvent with proton tautomerism. Proteins Struct. Funct. Bioinf. 2014, 82, 1319–1331. 10.1002/prot.24499. PMC4394622 24375620

[ref27] Lee J.; Miller B. T.; Damjanovic A.; Brooks B. R. Constant pH molecular dynamics in explicit solvent with enveloping distribution sampling and Hamiltonian exchange. J. Chem. Theory Comput. 2014, 10, 2738–2750. 10.1021/ct500175m. 25061443 PMC4095908

[ref28] Swails J. M.; York D. M.; Roitberg A. E. Constant pH replica exchange molecular dynamics in explicit solvent using discrete protonation states: implementation testing, and validation. J. Chem. Theory Comput. 2014, 10, 1341–1352. 10.1021/ct401042b. 24803862 PMC3985686

[ref29] Magalhães P. R.; Machuqueiro M.; Baptista A. M. Constant-pH Molecular Dynamics Study of Kyotorphin in an Explicit Bilayer. Biophys. J. 2015, 108, 2282–2290. 10.1016/j.bpj.2015.03.052. 25954885 PMC4423061

[ref30] Vila-Viçosa D.; Teixeira V. H.; Baptista A. M.; Machuqueiro M. Constant-pH MD simulations of an oleic acid bilayer. J. Chem. Theory Comput. 2015, 11, 2367–2376. 10.1021/acs.jctc.5b00095. 26574431

[ref31] Santos H. A.; Vila-Viçosa D.; Teixeira V. H.; Baptista A. M.; Machuqueiro M. Constant-pH MD simulations of DMPA/DMPC lipid bilayers. J. Chem. Theory Comput. 2015, 11, 5973–5979. 10.1021/acs.jctc.5b00956. 26588046

[ref32] Teixeira V. H.; Vila-Viçosa D.; Reis P. B.; Machuqueiro M. p*K*_a_ Values of Titrable Amino Acids at the Water/Membrane Interface. J. Chem. Theory Comput. 2016, 12, 930–934. 10.1021/acs.jctc.5b01114. 26863409

[ref33] Huang Y.; Chen W.; Wallace J. A.; Shen J. All-atom continuous constant pH molecular dynamics with particle mesh Ewald and titratable water. J. Chem. Theory Comput. 2016, 12, 5411–5421. 10.1021/acs.jctc.6b00552. 27709966 PMC5713900

[ref34] Radak B. K.; Chipot C.; Suh D.; Jo S.; Jiang W.; Phillips J. C.; Schulten K.; Roux B. Constant-pH Molecular Dynamics Simulations for Large Biomolecular Systems. J. Chem. Theory Comput. 2017, 13, 5933–5934. 10.1021/acs.jctc.7b00875. 29111720 PMC5726918

[ref35] Dobrev P.; Donnini S.; Groenhof G.; Grubmüller H. Accurate Three States Model for Amino Acids with Two Chemically Coupled Titrating Sites in Explicit Solvent Atomistic Constant pH Simulations and pKa Calculations. J. Chem. Theory Comput. 2017, 13, 147–160. 10.1021/acs.jctc.6b00807. 27966355

[ref36] Huang Y.; Harris R. C.; Shen J. Generalized Born based continuous constant pH molecular dynamics in Amber: Implementation, benchmarking and analysis. J. Chem. Inf. Model. 2018, 58, 1372–1383. 10.1021/acs.jcim.8b00227. 29949356 PMC6516748

[ref37] Vila-Viçosa D.; Reis P. B. P. S.; Baptista A. M.; Oostenbrink C.; Machuqueiro M. A pH Replica Exchange Scheme in the Stochastic Titration Constant-pH MD Method. J. Chem. Theory Comput. 2019, 15, 3108–3116. 10.1021/acs.jctc.9b00030. 30908045

[ref38] Barroso da Silva F. L.; Sterpone F.; Derreumaux P. OPEP6: A new constant-pH molecular dynamics simulation scheme with OPEP coarse-grained force field. J. Chem. Theory Comput. 2019, 15, 3875–3888. 10.1021/acs.jctc.9b00202. 31059255

[ref39] Oliveira N. F. B.; Pires I. D. S.; Machuqueiro M. Improved GROMOS 54A7 Charge Sets for Phosphorylated Tyr, Ser, and Thr to Deal with pH-Dependent Binding Phenomena. J. Chem. Theory Comput. 2020, 16, 6368–6376. 10.1021/acs.jctc.0c00529. 32809819

[ref40] Antosiewicz J. M.; Długosz M. Constant-pH Brownian dynamics simulations of a protein near a charged surface. ACS Omega 2020, 5, 30282–30298. 10.1021/acsomega.0c04817. 33251463 PMC7689933

[ref41] Reilley D. J.; Wang J.; Dokholyan N. V.; Alexandrova A. N. Titr-DMD - A Rapid, Coarse-Grained Quasi-All-Atom Constant pH Molecular Dynamics Framework. J. Chem. Theory Comput. 2021, 17, 4538–4549. 10.1021/acs.jctc.1c00338. 34165292 PMC10662685

[ref42] da Rocha L.; Baptista A. M.; Campos S. R. Approach to Study pH-Dependent Protein Association Using Constant-pH Molecular Dynamics: Application to the Dimerization of β-Lactoglobulin. J. Chem. Theory Comput. 2022, 18, 1982–2001. 10.1021/acs.jctc.1c01187. 35171602 PMC9775224

[ref43] Buslaev P.; Aho N.; Jansen A.; Bauer P.; Hess B.; Groenhof G. Best practices in constant pH MD simulations: accuracy and sampling. ChemRxiv, May 18, 2022, ver. 1. 10.26434/chemrxiv-2022-c6lg2. PMC9558372 36107791

[ref44] Oliveira N. F.; Machuqueiro M. Novel US-CpHMD Protocol to Study the Protonation-Dependent Mechanism of the ATP/ADP Carrier. J. Chem. Inf. Model. 2022, 62, 2550–2560. 10.1021/acs.jcim.2c00233. 35442654 PMC9775199

[ref45] Nielsen J. E.; Gunner M.; Garcia-Moreno E. B. The pKa Cooperative: A collaborative effort to advance structure-based calculations of pKa values and electrostatic effects in proteins. Proteins: Struct., Funct., Bioinf. 2011, 79, 3249–3259. 10.1002/prot.23194. PMC3375608 22002877

[ref46] Alexov E.; Mehler E. L.; Baker N.; M. Baptista A.; Huang Y.; Milletti F.; Erik Nielsen J.; Farrell D.; Carstensen T.; Olsson M. H.; et al. Progress in the prediction of pKa values in proteins. Proteins Struct. Funct. Bioinf. 2011, 79, 3260–3275. 10.1002/prot.23189. PMC3243943 22002859

[ref47] Schmid N.; Eichenberger A. P.; Choutko A.; Riniker S.; Winger M.; Mark A. E.; van Gunsteren W. F. Definition and testing of the GROMOS force-field versions 54A7 and 54B7. Eur. Biophys. J. 2011, 40, 843 10.1007/s00249-011-0700-9. 21533652

[ref48] Huang W.; Lin Z.; van Gunsteren W. F. Validation of the GROMOS 54A7 Force Field with Respect to β-Peptide Folding. J. Chem. Theory Comput. 2011, 7, 1237–1243. 10.1021/ct100747y. 26610119

[ref49] Huang J.; Rauscher S.; Nawrocki G.; Ran T.; Feig M.; de Groot B. L.; Grubmüller H.; MacKerell A. D. CHARMM36m: an improved force field for folded and intrinsically disordered proteins. Nat. Methods 2017, 14, 71–73. 10.1038/nmeth.4067. 27819658 PMC5199616

[ref50] Darden T.; York D.; Pedersen L. Particle mesh Ewald: An Nlog(N) method for Ewald sums in large systems. J. Chem. Phys. 1993, 98, 10089–10092. 10.1063/1.464397.

[ref51] Vila-Viçosa D.; Teixeira V. H.; Santos H. A. F.; Baptista A. M.; Machuqueiro M. Treatment of ionic strength in biomolecular simulations of charged lipid bilayers. J. Chem. Theory Comput. 2014, 10, 5483–5492. 10.1021/ct500680q. 26583231

[ref52] Silva T. F.; Vila-Viçosa D.; Reis P. B.; Victor B. L.; Diem M.; Oostenbrink C.; Machuqueiro M. The impact of using single atomistic long-range cutoff schemes with the GROMOS 54A7 force field. J. Chem. Theory Comput. 2018, 14, 5823–5833. 10.1021/acs.jctc.8b00758. 30354115

[ref53] Dissanayake T.; Swails J. M.; Harris M. E.; Roitberg A. E.; York D. M. Interpretation of pH-activity profiles for acid-base catalysis from molecular simulations. Biochemistry 2015, 54, 1307–1313. 10.1021/bi5012833. 25615525 PMC4441796

[ref54] Reis P. B.; Vila-Viçosa D.; Campos S. R.; Baptista A. M.; Machuqueiro M. Role of Counterions in Constant-pH Molecular Dynamics Simulations of PAMAM Dendrimers. ACS Omega 2018, 3, 2001–2009. 10.1021/acsomega.7b01708. 30023821 PMC6045380

[ref55] Abraham M. J.; Murtola T.; Schulz R.; Páll S.; Smith J. C.; Hess B.; Lindahl E. GROMACS: High performance molecular simulations through multi-level parallelism from laptops to supercomputers. SoftwareX 2015, 1–2, 19–25. 10.1016/j.softx.2015.06.001.

[ref56] Hub J. S.; de Groot B. L.; Grubmüller H.; Groenhof G. Quantifying Artifacts in Ewald Simulations of Inhomogeneous Systems with a Net Charge. J. Chem. Theory Comput. 2014, 10, 381–390. 10.1021/ct400626b. 26579917

[ref57] Donnini S.; Ullmann R. T.; Groenhof G.; Grubmueller H. Charge-neutral constant pH molecular dynamics simulations using a parsimonious proton buffer. J. Chem. Theory Comput. 2016, 12, 1040–1051. 10.1021/acs.jctc.5b01160. 26881315

[ref58] Aho N.; Buslaev P.; Jansen A.; Bauer P.; Groenhof G.; Hess B. Scalable Constant pH Molecular Dynamics in GROMACS. ChemRxiv, January 27, 2022, ver. 2. 10.26434/chemrxiv-2022-n025t-v2. PMC9558312 36128977

[ref59] Thurlkill R. L.; Grimsley G. R.; Scholtz J. M.; Pace C. N. p*K* values of the ionizable groups of proteins. Protein Sci. 2006, 15, 1214–1218. 10.1110/ps.051840806. 16597822 PMC2242523

[ref60] Grimsley G. R.; Scholtz J. M.; Pace C. N. A summary of the measured p*K* values of the ionizable groups in folded proteins. Protein Sci. 2009, 18, 247–251. 10.1002/pro.19. 19177368 PMC2708032

[ref61] Khandogin J.; Brooks C. L. III Toward the accurate first-principles prediction of ionization equilibria in proteins. Biochemistry-US 2006, 45, 9363–9373. 10.1021/bi060706r. 16878971

[ref62] Machuqueiro M.; Baptista A. M. Acidic range titration of HEWL using a constant-pH molecular dynamics method. Proteins Struct. Funct. Bioinf. 2008, 72, 289–298. 10.1002/prot.21923. 18214978

[ref63] Williams S. L.; de Oliveira C. A. F.; McCammon J. A. Coupling constant pH molecular dynamics with accelerated molecular dynamics. J. Chem. Theory Comput. 2010, 6, 560–568. 10.1021/ct9005294. 20148176 PMC2817915

[ref64] Demchuk E.; Wade R. C. Improving the Continuum Dielectric Approach to Calculating pKas of Ionizable Groups in Proteins. J. Phys. Chem. 1996, 100, 17373–17387. 10.1021/jp960111d.

[ref65] Kuramitsu S.; Hamaguchi K. Analysis of the acid-base titration curve of hen lysozyme. J. Biochem. 1980, 87, 1215–1219. 6771251

[ref66] Bartik K.; Redfield C.; Dobson C. M. Measurement of the individual pKa values of acidic residues of hen and turkey lysozymes by two-dimensional 1H NMR. Biophys. J. 1994, 66, 1180–1184. 10.1016/S0006-3495(94)80900-2. 8038389 PMC1275825

[ref67] Webb H.; Tynan-Connolly B. M.; Lee G. M.; Farrell D.; O’Meara F.; Søndergaard C. R.; Teilum K.; Hewage C.; McIntosh L. P.; Nielsen J. E. Remeasuring HEWL pKa values by NMR spectroscopy: Methods analysis, accuracy, and implications for theoretical pKa calculations. Proteins Struct. Funct. Bioinf. 2011, 79, 685–702. 10.1002/prot.22886. 21287606

[ref68] Lee K. K.; Fitch C. A.; Lecomte J. T.; García-Moreno E. B. Electrostatic effects in highly charged proteins: salt sensitivity of p K a values of histidines in staphylococcal nuclease. Biochemistry 2002, 41, 5656–5667. 10.1021/bi0119417. 11969427

[ref69] Castaneda C. A.; Fitch C. A.; Majumdar A.; Khangulov V.; Schlessman J. L.; García-Moreno B. E. Molecular determinants of the p*K*_a_ values of Asp and Glu residues in staphylococcal nuclease. Proteins Struct. Funct. Bioinf. 2009, 77, 570–588. 10.1002/prot.22470. 19533744

[ref70] Baptista A. M.; Soares C. M. Some Theoretical and Computational Aspects of the Inclusion of Proton Isomerism in the Protonation Equilibrium of Proteins. J. Phys. Chem. B 2001, 105, 293–309. 10.1021/jp002763e.

[ref71] Teixeira V. H.; Cunha C. A.; Machuqueiro M.; Oliveira A. S. F.; Victor B. L.; Soares C. M.; Baptista A. M. On the use of different dielectric constants for computing individual and pairwise terms in Poisson-Boltzmann studies of protein ionization equilibrium. J. Phys. Chem. B 2005, 109, 14691–14706. 10.1021/jp052259f. 16852854

[ref72] Walsh M. A.; Schneider T. R.; Sieker L. C.; Dauter Z.; Lamzin V. S.; Wilson K. S. Refinement of triclinic hen egg-white lysozyme at atomic resolution. Acta Crystallographica Section D: Biological Crystallography 1998, 54, 522–546. 10.1107/S0907444997013656. 9761848

[ref73] Hynes T. R.; Fox R. O. The crystal structure of staphylococcal nuclease refined at 1.7 Å resolution. Proteins: StructureFunction, and Bioinformatics 1991, 10, 92–105. 10.1002/prot.340100203. 1896431

[ref74] Qin J.; Clore G. M.; Gronenborn A. M. The high-resolution three-dimensional solution structures of the oxidized and reduced states of human thioredoxin. Structure 1994, 2, 503–522. 10.1016/S0969-2126(00)00051-4. 7922028

[ref75] Katti S. K.; LeMaster D. M.; Eklund H. Crystal structure of thioredoxin from Escherichia coli at 1.68 Å resolution. Journal of molecular biology 1990, 212, 167–184. 10.1016/0022-2836(90)90313-B. 2181145

[ref76] The PyMOL Molecular Graphics System, ver. 2.4; Schrödinger, LLC, 2020.

[ref77] Olsson M. H.; Søndergaard C. R.; Rostkowski M.; Jensen J. H. PROPKA3: consistent treatment of internal and surface residues in empirical p*K*_a_ predictions. J. Chem. Theory Comput. 2011, 7, 525–537. 10.1021/ct100578z. 26596171

[ref78] Anandakrishnan R.; Aguilar B.; Onufriev A. V. H. H++ 3.0: automating pK prediction and the preparation of biomolecular structures for atomistic molecular modeling and simulations. Nucleic Acids Res. 2012, 40, W537–W541. 10.1093/nar/gks375. 22570416 PMC3394296

[ref79] Wang L.; Zhang M.; Alexov E. DelPhiPKa web server: predicting p K a of proteins, RNAs and DNAs. Bioinformatics 2016, 32, 614–615. 10.1093/bioinformatics/btv607. 26515825 PMC5963359

[ref80] Song Y.; Mao J.; Gunner M. MCCE2: improving protein p*K*_a_ calculations with extensive side chain rotamer sampling. J. Comput. Chem. 2009, 30, 2231–2247. 10.1002/jcc.21222. 19274707 PMC2735604

[ref81] Reis P. B.; Vila-Viçosa D.; Rocchia W.; Machuqueiro M. PypKa: A Flexible Python Module for Poisson-Boltzmann-Based p K a Calculations. J. Chem. Inf. Model. 2020, 60, 4442–4448. 10.1021/acs.jcim.0c00718. 32857502

[ref82] Hess B. P-LINCS: A Parallel Linear Constraint Solver for Molecular Simulation. J. Chem. Theory Comput. 2008, 4, 116–122. 10.1021/ct700200b. 26619985

[ref83] Miyamoto S.; Kollman P. A. SETTLE: An analytical version of the SHAKE and RATTLE algorithm for rigid water models. J. Comput. Chem. 1992, 13, 952–962. 10.1002/jcc.540130805.

[ref84] Bussi G.; Donadio D.; Parrinello M. Canonical sampling through velocity rescaling. J. Chem. Phys. 2007, 126, 014101 10.1063/1.2408420. 17212484

[ref85] Parrinello M.; Rahman A. Polymorphic transitions in single crystals: A new molecular dynamics method. J. Appl. Phys. 1981, 52, 7182–7190. 10.1063/1.328693.

[ref86] Rocchia W.; Sridharan S.; Nicholls A.; Alexov E.; Chiabrera A.; Honig B. Rapid grid-based construction of the molecular surface and the use of induced surface charge to calculate reaction field energies: Applications to the molecular systems and geometric objects. J. Comput. Chem. 2002, 23, 128–137. 10.1002/jcc.1161. 11913378

[ref87] Gilson M. K.; Sharp K. A.; Honig B. Calculating the Eletrostatic Potential of Molecules in Solution: Method and Error Assessment. J. Comput. Chem. 1988, 9, 327–335. 10.1002/jcc.540090407.

[ref88] Teixeira V. H.; Vila-Viçosa D.; Baptista A. M.; Machuqueiro M. Protonation of DMPC in a Bilayer Environment Using a Linear Response Approximation. J. Chem. Theory Comput. 2014, 10, 2176–2184. 10.1021/ct5000082. 26580542

[ref89] Williams T.; Kelley C. Gnuplot 4.6: an interactive plotting program; 2013. http://gnuplot.sourceforge.net/.

[ref90] Tanford C.; Roxby R. Interpretation of protein titration curves Application to lysozyme. Biochemistry 1972, 11, 2192–2198. 10.1021/bi00761a029. 5027621

[ref91] Visser A.; van Engelen J.; Visser N.; van Hoek A.; Hilhorst R.; Freedman R. Fluorescence dynamics of staphylococcal nuclease in aqueous solution and reversed micelles. Biochimica et Biophysica Acta (BBA) - Protein Structure and Molecular Enzymology 1994, 1204, 225–234. 10.1016/0167-4838(94)90012-4. 8142463

[ref92] Wollman E.; d’Auriol L.; Rimsky L.; Shaw A.; Jacquot J.; Wingfield P.; Graber P.; Dessarps F.; Robin P.; Galibert F. Cloning and expression of a cDNA for human thioredoxin. J. Biol. Chem. 1988, 263, 15506–15512. 10.1016/S0021-9258(19)37617-3. 3170595

[ref93] Stefanková P.; Barák I. Thioredoxin - structural and functional complexity. Gen. Physiol. Biophys. 2005, 24, 3–11. 15900083

[ref94] Pahari S.; Sun L.; Alexov E. PKAD: a database of experimentally measured pKa values of ionizable groups in proteins. Database 2019, 2019, baz024 10.1093/database/baz024. 30805645 PMC6389863

[ref95] Reis P. B.; Clevert D.-A.; Machuqueiro M. pKPDB: a protein data bank extension database of pKa and pI theoretical values. Bioinformatics 2021, 38, 297–298. 10.1093/bioinformatics/btab518. 34260689

[ref96] Dyson H. J.; Jeng M.-F.; Tennant L. L.; Slaby I.; Lindell M.; Cui D.-S.; Kuprin S.; Holmgren A. Effects of buried charged groups on cysteine thiol ionization and reactivity in Escherichia coli thioredoxin: structural and functional characterization of mutants of Asp 26 and Lys 57. Biochemistry 1997, 36, 2622–2636. 10.1021/bi961801a. 9054569

[ref97] Nielsen J. E.; McCammon J. A. On the evaluation and optimization of protein X-ray structures for pKa calculations. Protein Sci. 2003, 12, 313–326. 10.1110/ps.0229903. 12538895 PMC2312414

[ref98] Qin J.; Clore G. M.; Gronenborn A. M. Ionization equilibria for side-chain carboxyl groups in oxidized and reduced human thioredoxin and in the complex with its target peptide from the transcription factor NFκB. Biochemistry 1996, 35, 7–13. 10.1021/bi952299h. 8555200

[ref99] Reis P.; Bertolini M.; Montanari F.; Rocchia W.; Machuqueiro M.; Clevert D.-A. A fast and interpretable deep learning approach for accurate electrostatics-driven pKa predictions in proteins. J. Chem. Theory Comput. 2022, 18, 5068 10.1021/acs.jctc.2c00308. 35837736 PMC9369009

