# Supplementary material for: Extending the Stochastic Titration CpHMD to CHARMM36m
Source: J Phys Chem B. 2022 Oct 3;126(40):7870–82. doi: 10.1021/acs.jpcb.2c04529 (PMC9776569; doi:10.1021/acs.jpcb.2c04529)
Supplement: Supplementary file 1 — jp2c04529_si_001.pdf [file jp2c04529_si_001.pdf]

Supporting Information: Extending the stochastic titration CpHMD to CHARMM36m

João G. N. Sequeira,[†,‡] Filipe E. P. Rodrigues,[†,‡] Telmo G. D. Silva,[†] Pedro B.P.S. Reis,[*,†] and Miguel Machuqueiro[*,†]

†*BioISI – Instituto de Biossistemas e Ciências Integrativas, Faculdade de Ciências, Universidade de Lisboa, 1749-016, Lisboa, Portugal*

‡*Equal Contribution*

E-mail: pdreis@ciencias.ulisboa.pt; machuque@ciencias.ulisboa.pt

Phone: +351-21-7500112

Results

Table S1: Number of ions used in the GROMOS 54A7 (G^{54A7}) and CHARMM36m(C^{36m}) simulations, with pH ranging from 1-12, for each biological system.

Protein	FF	pH											
		1	2	3	4	5	6	7	8	9	10	11	12
HEWL	G^{54A7}	18 Cl^-	18 Cl^-	15 Cl^-	12 Cl^-	9 Cl^-	8 Cl^-	7 Cl^-	7 Cl^-	6 Cl^-	4 Cl^-	2 Cl^-	0
	C^{36m}	19 Cl^-	18 Cl^-	16 Cl^-	13 Cl^-	10 Cl^-	9 Cl^-	7 Cl^-	7 Cl^-	6 Cl^-	4 Cl^-	1 Cl^-	1 Na^+
SNase	G^{54A7}	31 Cl^-	30 Cl^-	25 Cl^-	17 Cl^-	12 Cl^-	9 Cl^-	8 Cl^-	7 Cl^-	5 Cl^-	2 Na^+	11 Na^+	18 Na^+
	C^{36m}	32 Cl^-	30 Cl^-	27 Cl^-	22 Cl^-	15 Cl^-	10 Cl^-	8 Cl^-	7 Cl^-	5 Cl^-	0	10 Na^+	17 Na^+
^{h}Trx	G^{54A7}	14 Cl^-	14 Cl^-	12 Cl^-	6 Cl^-	0	3 Na^+	5 Na^+	6 Na^+	7 Na^+	9 Na^+	14 Na^+	17 Na^+
	C^{36m}	14 Cl^-	14 Cl^-	12 Cl^-	7 Cl^-	1 Cl^-	3 Na^+	5 Na^+	5 Na^+	6 Na^+	7 Na^+	12 Na^+	17 Na^+
^{Ec}Trx	G^{54A7}	13 Cl^-	12 Cl^-	10 Cl^-	4 Cl^-	1 Na^+	3 Na^+	5 Na^+	6 Na^+	6 Na^+	9 Na^+	12 Na^+	16 Na^+
	C^{36m}	13 Cl^-	12 Cl^-	10 Cl^-	6 Cl^-	0	4 Na^+	5 Na^+	5 Na^+	6 Na^+	8 Na^+	13 Na^+	17 Na^+

Table S2: pK^{mod} of all titrable residues for the Null model and for both force fields used. The Null model values were obtained from experimental pK_{a} measured with the AAXAA pentapeptides, where X is the a.a. of interest.[S1,S2]

Residue	Null	G^{54A7}	C^{36m}
CTr	3.67	2.93	1.90
Asp	3.94	3.94	3.90
Glu	4.25	4.24	4.11
His	6.54	6.79	6.78
NTr	8.00	8.08	9.01
Cys	8.55	8.62	8.46
Lys	10.40	10.49	10.49

Table S3: Isoelectric points calculated with the PypKa software[S3] and with the CpHMD simulations using G^{54A7} and C^{36m} force fields. The experimental values[S4–S7] are also shown for comparison.

System	exp. pI	PypKa	G^{54A7}	C^{36m}
HEWL	∼11.0 [Ref. S4]	11.4	11.3	>12.0
SNase	9.6 [Ref. S5]	10.0	9.8	10.1
^{h}Trx	4.7 [Ref. S6]	5.2	5.1	5.0
^{Ec}Trx	4.5 [Ref. S7]	5.0	4.9	4.8

Table S4: HEWL pK_a predictions using st-CpHMD with G^{54A7} and C^{36m} for all titrating residues. The difference between predictions is presented (Diff = G^{54A7} – C^{36m}). Predictions outside of the used pH range were excluded, to avoid extrapolation.

Residue	G^{54A7}	C^{36m}	Diff
	pK_a	pK_a	
NTr-1	6.67 ± 0.08	5.96 ± 0.09	0.71
Lys-1	10.50 ± 0.04	10.18 ± 0.01	0.32
Glu-7	3.55 ± 0.04	3.19 ± 0.04	0.36
Lys-13	10.76 ± 0.06	9.96 ± 0.06	0.80
His-15	5.67 ± 0.07	4.61 ± 0.07	1.06
Asp-18	3.99 ± 0.07	3.18 ± 0.07	0.81
Tyr-20	10.04 ± 0.25	>12.0	—
Tyr-23	11.16 ± 0.28	>12.0	—
Lys-33	9.28 ± 0.04	9.09 ± 0.11	0.19
Glu-35	6.02 ± 0.17	5.99 ± 0.32	0.03
Asp-48	2.35 ± 0.20	3.04 ± 0.27	-0.69
Asp-52	4.97 ± 0.23	4.99 ± 0.09	-0.02
Tyr-53	11.44 ± 0.06	>12.0	—
Asp-66	3.92 ± 0.15	2.38 ± 0.21	1.54
Asp-87	2.60 ± 0.05	2.42 ± 0.04	0.17
Lys-96	10.47 ± 0.14	9.23 ± 0.03	1.25
Lys-97	10.33 ± 0.04	10.11 ± 0.02	0.22
Asp-101	3.87 ± 0.20	4.11 ± 0.09	-0.24
Lys-116	10.35 ± 0.07	9.83 ± 0.02	0.52
Asp-119	3.31 ± 0.17	2.77 ± 0.05	0.54
CTr-129	3.41 ± 0.05	3.38 ± 0.03	0.03

Table S5: SNase pK_a predictions using st-CpHMD with G^{54A7} and C^{36m} for all titrating residues. The difference between predictions is presented (Diff = G^{54A7} – C^{36m}). Predictions outside of the used pH range were excluded, to avoid extrapolation.

Residue	G^{54A7}	C^{36m}	Diff
	pK_a	pK_a	
NTr-1	7.25 ± 0.05	7.78 ± 0.05	-0.53
Lys-5	10.06 ± 0.12	10.32 ± 0.01	-0.26
Lys-6	10.49 ± 0.04	10.23 ± 0.11	0.26
His-8	5.72 ± 0.05	5.97 ± 0.03	-0.25
Lys-9	>12.0	9.80 ± 0.12	—
Glu-10	4.06 ± 0.22	3.22 ± 0.05	0.84
Lys-16	10.45 ± 0.03	10.32 ± 0.02	0.13
Asp-19	2.66 ± 0.13	2.51 ± 0.07	0.15
Asp-21	4.58 ± 0.06	4.43 ± 0.04	0.14
Lys-24	9.87 ± 0.09	9.56 ± 0.09	0.31
Tyr-27	>12.0	11.96 ± 0.25	—
Lys-28	10.65 ± 0.05	10.13 ± 0.10	0.53
Asp-40	4.12 ± 0.02	3.49 ± 0.10	0.62
Glu-43	4.07 ± 0.09	4.29 ± 0.11	-0.21
Lys-45	10.93 ± 0.03	10.55 ± 0.08	0.38
His-46	3.97 ± 1.00	2.09 ± 0.64	1.88
Lys-48	10.54 ± 0.01	10.47 ± 0.03	0.07
Lys-49	11.10 ± 0.08	10.71 ± 0.06	0.39
Glu-52	5.28 ± 0.11	5.04 ± 0.25	0.24
Lys-53	11.10 ± 0.03	10.86 ± 0.02	0.24
Tyr-54	>12.0	>12.0	—
Glu-57	4.37 ± 0.08	4.29 ± 0.10	0.08
Lys-63	11.08 ± 0.02	10.25 ± 0.20	0.83
Lys-64	10.65 ± 0.02	10.67 ± 0.02	-0.01
Glu-67	4.72 ± 0.02	3.74 ± 0.05	0.98
Lys-70	10.75 ± 0.03	10.86 ± 0.02	-0.11
Lys-71	10.74 ± 0.04	10.59 ± 0.04	0.15
Glu-73	4.79 ± 0.02	3.48 ± 0.08	1.31
Glu-75	6.32 ± 0.15	5.41 ± 0.20	0.91
Asp-77	0.76 ± 10.08	0.90 ± 0.74	-0.15
Lys-78	10.19 ± 0.11	10.46 ± 0.01	-0.26
Asp-83	3.33 ± 0.24	2.92 ± 0.26	0.41

Lys-84	10.80 ± 0.04	10.55 ± 0.06	0.25
Tyr-85	9.51 ± 0.01	9.77 ± 0.09	-0.26
Tyr-91	>12.0	-8.99 ± 0.90	—
Tyr-93	>12.0	>12.0	—
Asp-95	2.95 ± 0.23	3.08 ± 0.03	-0.13
Lys-97	10.32 ± 0.02	10.72 ± 0.03	-0.40
Glu-101	3.06 ± 0.17	3.69 ± 0.13	-0.63
Lys-110	11.56 ± 0.13	11.37 ± 0.04	0.19
Tyr-113	9.99 ± 0.05	9.99 ± 0.01	0.00
Tyr-115	10.69 ± 0.36	10.48 ± 0.24	0.21
Lys-116	10.51 ± 0.02	10.48 ± 0.03	0.03
His-121	3.40 ± 0.17	1.00 ± 1.35	2.40
Glu-122	5.06 ± 0.20	4.38 ± 0.14	0.68
His-124	4.70 ± 0.03	4.80 ± 0.21	-0.10
Lys-127	10.34 ± 0.03	10.22 ± 0.03	0.12
Glu-129	4.79 ± 0.08	3.71 ± 0.06	1.08
Lys-133	11.25 ± 0.03	10.93 ± 0.06	0.32
Lys-134	10.49 ± 0.03	10.36 ± 0.01	0.13
Glu-135	4.44 ± 0.00	3.47 ± 0.06	0.97
Lys-136	11.14 ± 0.03	10.44 ± 0.02	0.70
Glu-142	4.04 ± 0.02	3.86 ± 0.02	0.19
Asp-143	3.73 ± 0.09	3.79 ± 0.02	-0.06
Asp-146	3.86 ± 0.16	3.58 ± 0.10	0.28
CTr-149	3.78 ± 0.07	3.53 ± 0.12	0.26

Table S6: ^{h}Trx pK_a predictions using st-CpHMD with G^{54A7} and C^{36m} for all titrating residues. The difference between predictions is presented (Diff = G^{54A7} – C^{36m}). Predictions outside of the used pH range were excluded, to avoid extrapolation.

Residue	G^{54A7}	C^{36m}	Diff
	pK_a	pK_a	
NTr-1	5.72 ± 0.07	6.89 ± 0.06	-1.17
Lys-3	10.85 ± 0.01	10.55 ± 0.07	0.30
Glu-6	4.65 ± 0.06	4.61 ± 0.03	0.04
Lys-8	11.22 ± 0.03	10.28 ± 0.08	0.94
Glu-13	4.58 ± 0.02	4.33 ± 0.01	0.26
Asp-16	4.20 ± 0.05	4.32 ± 0.04	-0.11
Asp-20	3.41 ± 0.05	3.19 ± 0.03	0.22
Lys-21	11.00 ± 0.04	10.42 ± 0.07	0.58
Asp-26	10.53 ± 0.17	6.94 ± 0.38	3.59
Lys-36	10.80 ± 0.02	10.49 ± 0.03	0.31
Lys-39	10.94 ± 0.08	11.70 ± 0.18	-0.77
His-43	6.04 ± 0.06	5.08 ± 0.07	0.96
Glu-47	4.22 ± 0.04	4.07 ± 0.02	0.15
Lys-48	11.02 ± 0.04	10.80 ± 0.01	0.21
Tyr-49	11.65 ± 0.13	>12.0	—
Glu-56	5.58 ± 0.16	3.65 ± 0.13	1.93
Asp-58	4.96 ± 0.22	5.31 ± 0.05	-0.35
Asp-60	3.32 ± 0.10	3.83 ± 0.06	-0.51
Asp-61	4.95 ± 0.15	4.61 ± 0.05	0.33
Asp-64	3.57 ± 0.09	3.37 ± 0.08	0.19
Glu-68	4.63 ± 0.02	4.08 ± 0.07	0.55
Glu-70	3.94 ± 0.04	4.15 ± 0.01	-0.21
Lys-72	11.08 ± 0.04	11.02 ± 0.01	0.06
Lys-81	11.54 ± 0.04	10.07 ± 0.06	1.47
Lys-82	10.92 ± 0.02	10.70 ± 0.02	0.23
Lys-85	11.18 ± 0.03	10.32 ± 0.05	0.86
Glu-88	4.05 ± 0.08	3.89 ± 0.02	0.16
Lys-94	10.46 ± 0.01	9.89 ± 0.06	0.57
Glu-95	3.59 ± 0.04	3.67 ± 0.02	-0.09
Lys-96	10.66 ± 0.01	10.67 ± 0.01	-0.01
Glu-98	4.75 ± 0.03	4.28 ± 0.02	0.47
Glu-103	4.70 ± 0.01	4.52 ± 0.03	0.18
CTr-105	4.42 ± 0.06	4.15 ± 0.01	0.26

Table S7: ^{Ec}Trx pK_a predictions using st-CpHMD with G^{54A7} and C^{36m} for all titrating residues. The difference between predictions is presented (Diff = G^{54A7} – C^{36m}). Predictions outside of the used pH range were excluded, to avoid extrapolation.

Residue	G^{54A7} pK_a	C^{36m} pK_a	Diff
NTr-1	5.78 ± 0.22	7.20 ± 0.03	-1.42
Asp-2	2.84 ± 0.08	2.87 ± 0.05	-0.02
Lys-3	11.75 ± 0.06	11.08 ± 0.11	0.66
His-6	5.87 ± 0.03	5.82 ± 0.07	0.05
Asp-9	4.28 ± 0.20	4.19 ± 0.10	0.09
Asp-10	3.18 ± 0.06	3.63 ± 0.06	-0.46
Asp-13	4.46 ± 0.07	3.77 ± 0.06	0.69
Asp-15	5.21 ± 0.23	5.22 ± 0.07	-0.01
Lys-18	11.05 ± 0.02	10.89 ± 0.01	0.16
Asp-20	4.06 ± 0.06	4.05 ± 0.02	0.00
Asp-26	9.38 ± 0.54	7.47 ± 0.03	1.91
Glu-30	3.33 ± 0.10	4.02 ± 0.04	-0.69
Cys-32	9.66 ± 0.11	11.81 ± 0.17	-2.14
Cys-35	10.84 ± 0.81	12.15 ± 0.50	-1.31
Lys-36	11.21 ± 0.10	10.48 ± 0.05	0.73
Asp-43	4.91 ± 0.10	3.34 ± 0.01	1.57
Glu-44	4.49 ± 0.03	3.70 ± 0.06	0.79
Asp-47	4.39 ± 0.07	3.56 ± 0.09	0.83
Glu-48	4.45 ± 0.06	4.20 ± 0.06	0.25
Tyr-49	>12.0	>12.0	—
Lys-52	11.06 ± 0.08	11.14 ± 0.00	-0.08
Lys-57	9.96 ± 0.23	10.87 ± 0.05	-0.91
Asp-61	3.56 ± 0.11	3.93 ± 0.10	-0.37
Lys-69	10.79 ± 0.19	10.84 ± 0.02	-0.05
Tyr-70	11.10 ± 0.32	>12.0	—
Lys-82	10.97 ± 0.11	9.85 ± 0.04	1.12
Glu-85	4.43 ± 0.01	4.44 ± 0.01	-0.01
Lys-90	10.21 ± 0.03	10.07 ± 0.07	0.14
Lys-96	11.21 ± 0.06	10.31 ± 0.10	0.90
Lys-100	11.28 ± 0.01	10.82 ± 0.02	0.46
Glu-101	4.27 ± 0.01	4.42 ± 0.06	-0.16
Asp-104	4.37 ± 0.04	4.00 ± 0.03	0.37
CTr-108	3.74 ± 0.12	3.31 ± 0.01	0.43

Table S8: HEWL experimental pK_a values (taken from PKAD[S8]) and pK_a predictions using PypKa, and CpHMD with G^{54A7} and C^{36m}. The differences (Diff) between the estimated and experimental pK_as are also shown.

Residue	pK_a^Exp	PypKa		G^{54A7}		C^{36m}	
		pK_a	Diff	pK_a	Diff	pK_a	Diff
NTr-1	7.90	7.44	-0.46	6.67 ± 0.08	-1.23	5.96 ± 0.09	-1.94
Lys-1	10.90	10.44	-0.46	10.50 ± 0.04	-0.40	10.18 ± 0.01	-0.72
Glu-7	2.85	3.30	0.45	3.55 ± 0.04	0.70	3.19 ± 0.04	0.34
Lys-13	10.60	11.22	0.63	10.76 ± 0.06	0.16	9.96 ± 0.06	-0.64
His-15	5.36	5.74	0.38	5.67 ± 0.07	0.31	4.61 ± 0.07	-0.75
Asp-18	2.66	3.19	0.53	3.99 ± 0.07	1.33	3.18 ± 0.07	0.52
Tyr-20	10.30	9.99	-0.31	10.04 ± 0.25	-0.26	>12.0	—
Tyr-23	9.80	9.63	-0.17	11.16 ± 0.28	1.36	>12.0	—
Lys-33	10.60	10.53	-0.06	9.28 ± 0.04	-1.32	9.09 ± 0.11	-1.51
Glu-35	6.20	4.75	-1.45	6.02 ± 0.17	-0.18	5.99 ± 0.32	-0.21
Asp-48	1.60	1.74	0.14	2.35 ± 0.20	0.75	3.04 ± 0.27	1.44
Asp-52	3.68	2.72	-0.96	4.97 ± 0.23	1.29	4.99 ± 0.09	1.31
Tyr-53	12.10	11.46	-0.64	11.44 ± 0.06	-0.66	>12.0	—
Asp-66	0.90	2.76	1.86	3.92 ± 0.15	3.02	2.38 ± 0.21	1.48
Asp-87	2.07	2.19	0.12	2.60 ± 0.05	0.53	2.42 ± 0.04	0.35
Lys-96	10.80	11.26	0.46	10.47 ± 0.14	-0.33	9.23 ± 0.03	-1.57
Lys-97	10.30	11.16	0.86	10.33 ± 0.04	0.03	10.11 ± 0.02	-0.19
Asp-101	4.09	3.76	-0.33	3.87 ± 0.20	-0.22	4.11 ± 0.09	0.02
Lys-116	10.40	10.10	-0.30	10.35 ± 0.07	-0.05	9.83 ± 0.02	-0.57
Asp-119	3.20	2.78	-0.42	3.31 ± 0.17	0.11	2.77 ± 0.05	-0.43
CTr-129	2.75	1.97	-0.78	3.41 ± 0.05	0.66	3.38 ± 0.03	0.63

Table S9: SNase experimental pK_a values (taken from PKAD[S8]) and pK_a predictions using PypKa, and CpHMD with G^{54A7} and C^{36m}. The differences (Diff) between the estimated and experimental pK_as are also shown.

Residue	pK_a^{Exp}	PypKa		G^{54A7}		C^{36m}	
		pK_a	Diff	pK_a	Diff	pK_a	Diff
His-8	6.52	6.60	0.08	5.72 ± 0.05	-0.80	5.97 ± 0.03	-0.55
Glu-10	2.82	3.15	0.33	4.06 ± 0.22	1.24	3.22 ± 0.05	0.40
Asp-40	3.87	3.38	-0.49	4.12 ± 0.02	0.25	3.49 ± 0.10	-0.38
Glu-43	4.32	5.04	0.72	4.07 ± 0.09	-0.25	4.29 ± 0.11	-0.03
His-46	5.86	1.23	-4.63	3.97 ± 1.00	-1.89	2.09 ± 0.64	-3.77
Glu-52	3.93	3.33	-0.60	5.28 ± 0.11	1.35	5.04 ± 0.25	1.11
Glu-57	3.49	3.77	0.28	4.37 ± 0.08	0.88	4.29 ± 0.10	0.80
Glu-67	3.76	2.45	-1.31	4.72 ± 0.02	0.96	3.74 ± 0.05	-0.02
Glu-73	3.31	3.80	0.49	4.79 ± 0.02	1.49	3.48 ± 0.08	0.17
Glu-75	3.26	2.15	-1.11	6.32 ± 0.15	3.06	5.41 ± 0.20	2.15
Asp-95	2.16	1.84	-0.32	2.95 ± 0.23	0.79	3.08 ± 0.03	0.92
Glu-101	3.81	3.15	-0.66	3.06 ± 0.17	-0.74	3.69 ± 0.13	-0.12
His-121	5.30	6.14	0.84	3.40 ± 0.17	-1.90	1.00 ± 1.35	-4.30
Glu-122	3.89	3.68	-0.21	5.06 ± 0.20	1.17	4.38 ± 0.14	0.49
His-124	5.73	6.45	0.71	4.70 ± 0.03	-1.03	4.80 ± 0.21	-0.93
Glu-129	3.75	2.43	-1.32	4.79 ± 0.08	1.04	3.71 ± 0.06	-0.04
Glu-135	3.76	2.47	-1.29	4.44 ± 0.00	0.68	3.47 ± 0.06	-0.29
Glu-142	4.49	4.24	-0.25	4.04 ± 0.02	-0.45	3.86 ± 0.02	-0.63
Asp-143	3.80	3.86	0.06	3.73 ± 0.09	-0.07	3.79 ± 0.02	-0.01
Asp-146	3.86	3.79	-0.07	3.86 ± 0.16	0.00	3.58 ± 0.10	-0.28

Table S10: ^{h}Trx experimental pK_a values (taken from PKAD[S8]) and pK_a predictions using PypKa, and CpHMD with G^{54A7} and C^{36m}. The differences (Diff) between the estimated and experimental pK_as are also shown.

Residue	pK_a^{Exp}	PypKa		G^{54A7}		C^{36m}	
		pK_a	Diff	pK_a	Diff	pK_a	Diff
Glu-6	4.80	4.29	-0.51	4.65 ± 0.06	-0.15	4.61 ± 0.03	-0.19
Glu-13	4.40	4.11	-0.29	4.58 ± 0.02	0.18	4.33 ± 0.01	-0.07
Asp-16	4.00	3.94	-0.05	4.20 ± 0.05	0.20	4.32 ± 0.04	0.32
Asp-20	3.80	3.90	0.10	3.41 ± 0.05	-0.39	3.19 ± 0.03	-0.61
Asp-26	9.90	5.02	-4.88	10.53 ± 0.17	0.62	6.94 ± 0.38	-2.96
Glu-47	4.10	3.74	-0.35	4.22 ± 0.04	0.12	4.07 ± 0.02	-0.03
Glu-56	3.10	2.59	-0.51	5.58 ± 0.16	2.48	3.65 ± 0.13	0.55
Asp-58	2.80	1.70	-1.10	4.96 ± 0.22	2.15	5.31 ± 0.05	2.51
Asp-60	4.20	4.02	-0.18	3.32 ± 0.10	-0.88	3.83 ± 0.06	-0.38
Asp-61	5.30	4.84	-0.46	4.95 ± 0.15	-0.35	4.61 ± 0.05	-0.69
Asp-64	3.20	2.88	-0.32	3.57 ± 0.09	0.37	3.37 ± 0.08	0.17
Glu-68	4.90	3.70	-1.20	4.63 ± 0.02	-0.27	4.08 ± 0.07	-0.82
Glu-70	4.60	4.08	-0.52	3.94 ± 0.04	-0.66	4.15 ± 0.01	-0.45
Glu-88	3.70	3.45	-0.25	4.05 ± 0.08	0.35	3.89 ± 0.02	0.19
Glu-95	4.10	3.95	-0.15	3.59 ± 0.04	-0.51	3.67 ± 0.02	-0.43
Glu-98	3.90	4.06	0.16	4.75 ± 0.03	0.85	4.28 ± 0.02	0.38
Glu-103	4.40	4.20	-0.20	4.70 ± 0.01	0.30	4.52 ± 0.03	0.12

Table S11: ^{Ec}Trx experimental pK_a values (taken from PKAD[S8]) and pK_a predictions using PypKa, and CpHMD with G^{54A7} and C^{36m}. The differences (Diff) between the estimated and experimental pK_as are also shown.

Residue	pK_a^{Exp}	PypKa		G^{54A7}		C^{36m}	
		pK_a	Diff	pK_a	Diff	pK_a	Diff
NTr-1	7.40	8.52	1.12	5.78 ± 0.22	-1.62	7.20 ± 0.03	-0.20
His-6	6.20	6.44	0.24	5.87 ± 0.03	-0.33	5.82 ± 0.07	-0.38
Asp-20	3.80	3.91	0.11	4.06 ± 0.06	0.26	4.05 ± 0.02	0.25
Asp-26	7.50	4.75	-2.74	9.38 ± 0.54	1.88	7.47 ± 0.03	-0.03
Cys-32	7.10	9.25	2.15	9.66 ± 0.11	2.56	11.81 ± 0.17	4.71
Cys-35	9.90	15.78	5.88	10.84 ± 0.81	0.94	12.15 ± 0.50	2.25

Table S12: Mean average error (MAE) of the pK_a predictions for each tested protein. The maximum deviation values observed for each case are shown in parenthesis.

System	PypKa	G^{54A7}		C^{36m}		ΔFFs	
		[0–10]	[10–50]	[0–10]	[10–50]	[0–10]	[10–50]
HEWL	0.59 (1.86)	0.61 (2.64)	0.70 (3.02)	0.75 (2.09)	0.81 (1.94)	0.53 (1.44)	0.53 (1.54)
SNase	0.79 (4.63)	0.97 (3.10)	1.05 (3.06)	0.98 (4.30)	0.87 (4.30)	0.45 (2.16)	0.49 (2.40)
^{h}Trx	0.66 (4.88)	0.60 (1.97)	0.64 (2.48)	0.61 (2.94)	0.64 (2.96)	0.55 (3.98)	0.56 (3.59)
^{Ec}Trx	2.04 (5.88)	1.68 (3.81)	1.26 (2.56)	1.49 (4.95)	1.30 (4.71)	0.50 (2.14)	0.63 (2.14)

Table S13: HEWL pK_a RMSE values of several CpHMD-based methods. Different studies may have used different sets of residues to calculate the RMSE. We have recalculated the RMSE values when a different set of experimental pK_a values was used.

Ref	Method	Force Field	RMSE
S9	st-CpHMD	G43A1	0.70
		G53A6	0.79
S10	pHRE	G54A7	0.83
S11	pH-REMD	AMBER ff99SB	0.82
S12	GBNeck2-CpHMD	AMBER ff14SB	0.97
		CHARMM C22	1.22

Table S14: RMSE values calculated from HEWL pK_a predictions obtained in this work and from the literature.[S12–S14] Since different residues are used in each work, we also recalculated the RMSE value using subsets of our data (shown in parenthesis).

Residues	Experimental	This Work	Huang et al 2018[S12]	Huang et al 2016[S13]	Goh et al 2014[S14]
NTr-1	7.9	5.96	–	–	–
Lys-1	10.9	10.18	–	–	–
Glu-7	2.85	3.19	3.5	3.2	2.7
Lys-13	10.6	9.96	–	–	–
His-15	5.36	4.61	6.5	4	6
Asp-18	2.66	3.18	1.1	2.9	2.1
Lys-33	10.6	9.09	–	–	–
**Glu-35**	6.2	5.99	4.6	7.1	7
Asp-48	1.6	3.04	1.8	0.9	1.3
Asp-52	3.68	4.99	3.3	5.6	4.5
Asp-66	0.9	2.38	3.1	1.1	1.5
Asp-87	2.07	2.42	1.8	2.3	1.3
Lys-96	10.8	9.23	–	–	–
Lys-97	10.3	10.11	–	–	–
Asp-101	4.09	4.11	4.8	5.2	5.1
Lys-116	10.4	9.83	–	–	–
Asp-119	3.2	2.77	2.4	3.5	1.6
CTr-129	2.75	3.38	–	–	–
**RMSE**		0.99	1.14 (0.85)	0.92 (0.85)	0.82 (0.85)

Table S15: RMSE values calculated from SNase pK_a predictions obtained in this work and from the literature.[S12,S13,S15] Since different residues are used in each work, we also recalculated the RMSE value using subsets of our data (shown in parenthesis).

Residues	Experimental	This Work	Huang et al 2018[S12]	Radak et al 2017[S15]	Huang et al 2016[S13]
His-8	6.52	5.97	6.5	6.66	–
Glu-10	2.82	3.22	3.7	3.23	3.2
Asp-40	3.87	3.49	2.8	3.32	2.9
Glu-43	4.32	4.29	3.7	4.44	4.1
His-46	5.86	2.09	–	–	–
Glu-52	3.93	5.04	3.9	5.01	4.7
Glu-57	3.49	4.29	3.4	4.85	4.1
Glu-67	3.76	3.74	4.5	4.23	4
Glu-73	3.31	3.48	3.9	3.48	3.6
**Glu-75**	3.26	5.41	2.6	2.98	2.7
Asp-95	2.16	3.08	4.3	2.74	3
Glu-101	3.81	3.69	3.5	4.55	4.7
**His-121**	5.3	1	6.8	5.36	–
Glu-122	3.89	4.38	3	3.9	4.4
His-124	5.73	4.8	–	–	–
Glu-129	3.75	3.71	4.5	5.5	5.5
Glu-135	3.76	3.47	4.2	2.9	2.9
Glu-142	4.49	3.86	–	–	–
Asp-143	3.8	3.79	–	4.41	–
Asp-146	3.86	3.58	–	4.01	–
**RMSE**		1.46	0.90 (1.34)	0.72 (1.26)	0.79 (0.79)

Table S16: RMSE values calculated from ^{h}Trx pK_a predictions obtained in this work and from the literature.[S12] Since different residues are used in each work, we also recalculated the RMSE value using subsets of our data (shown in parenthesis).

Residues	Experimental	This Work	Huang et al 2018[S12]
Glu-6	4.8	4.61	3.9
Glu-13	4.4	4.33	4.4
Asp-16	4	4.32	4
Asp-20	3.8	3.19	2.9
**Asp-26**	9.9	6.94	6.2
Glu-47	4.1	4.07	4.3
Glu-56	3.1	3.65	4.5
Asp-58	2.8	5.31	3.8
Asp-60	4.2	3.83	3.6
Asp-61	5.3	4.61	4.6
Asp-64	3.2	3.37	3.1
Glu-68	4.9	4.08	4.3
Glu-70	4.6	4.15	5
Glu-88	3.7	3.89	3.8
Glu-95	4.1	3.67	3.5
Glu-98	3.9	4.28	3.9
Glu-103	4.4	4.52	4.7
**RMSE**		1.02	1.09 (1.02)

Table S17: MAE values per force field for each titrating residue type. The maximum deviation values are in parenthesis.

Residue	Exp./Total residues	PypKa	G^{54A7}	C^{36m}	ΔFFs
CTr	1 / 4	0.78 (0.78)	0.66 (0.66)	0.63 (0.63)	0.24 (0.43)
Asp	20 / 33	0.76 (4.88)	0.77 (3.02)	0.75 (2.96)	0.53 (3.59)
Glu	24 / 29	0.61 (1.45)	0.84 (3.06)	0.42 (2.15)	0.50 (1.93)
Cys	2 / 2	4.01 (5.88)	1.75 (2.56)	3.48 (4.71)	1.73 (2.14)
His	6 / 7	1.15 (4.63)	1.04 (1.89)	1.78 (4.30)	0.96 (2.40)
NTr	2 / 4	0.79 (1.12)	1.42 (1.62)	1.07 (1.94)	0.96 (1.42)
Lys	6 / 51	0.46 (0.86)	0.38 (1.32)	0.87 (1.57)	0.45 (2.29)

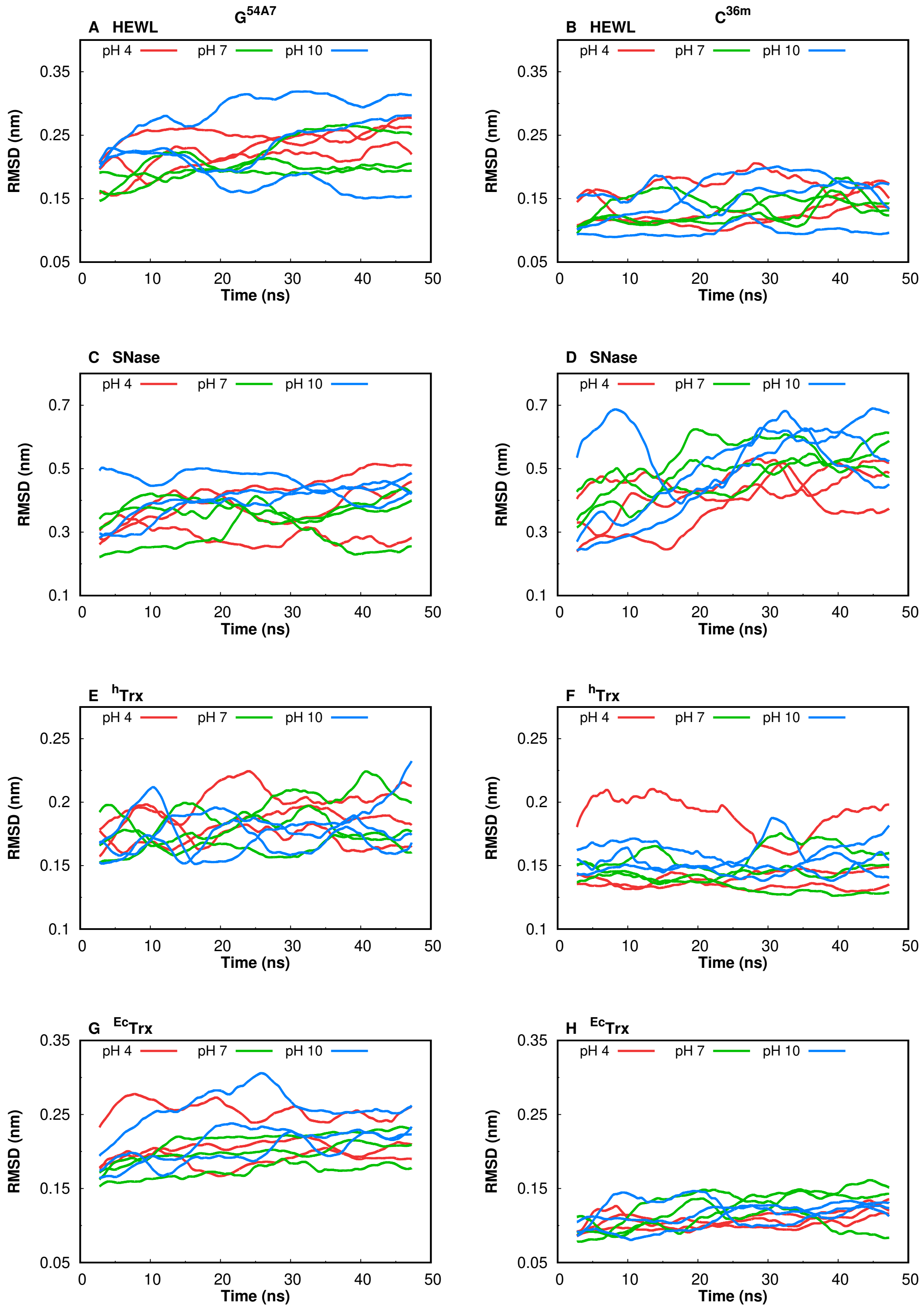

Figure S1: Cα RMSD values over time of all simulated systems using G^{54A7} and C^{36m}. Triplicates of acidic (4), neutral and alkaline pH (10) simulations are represented. A sliding window average (5 ns) was applied to remove the undesired fast fluctuations.

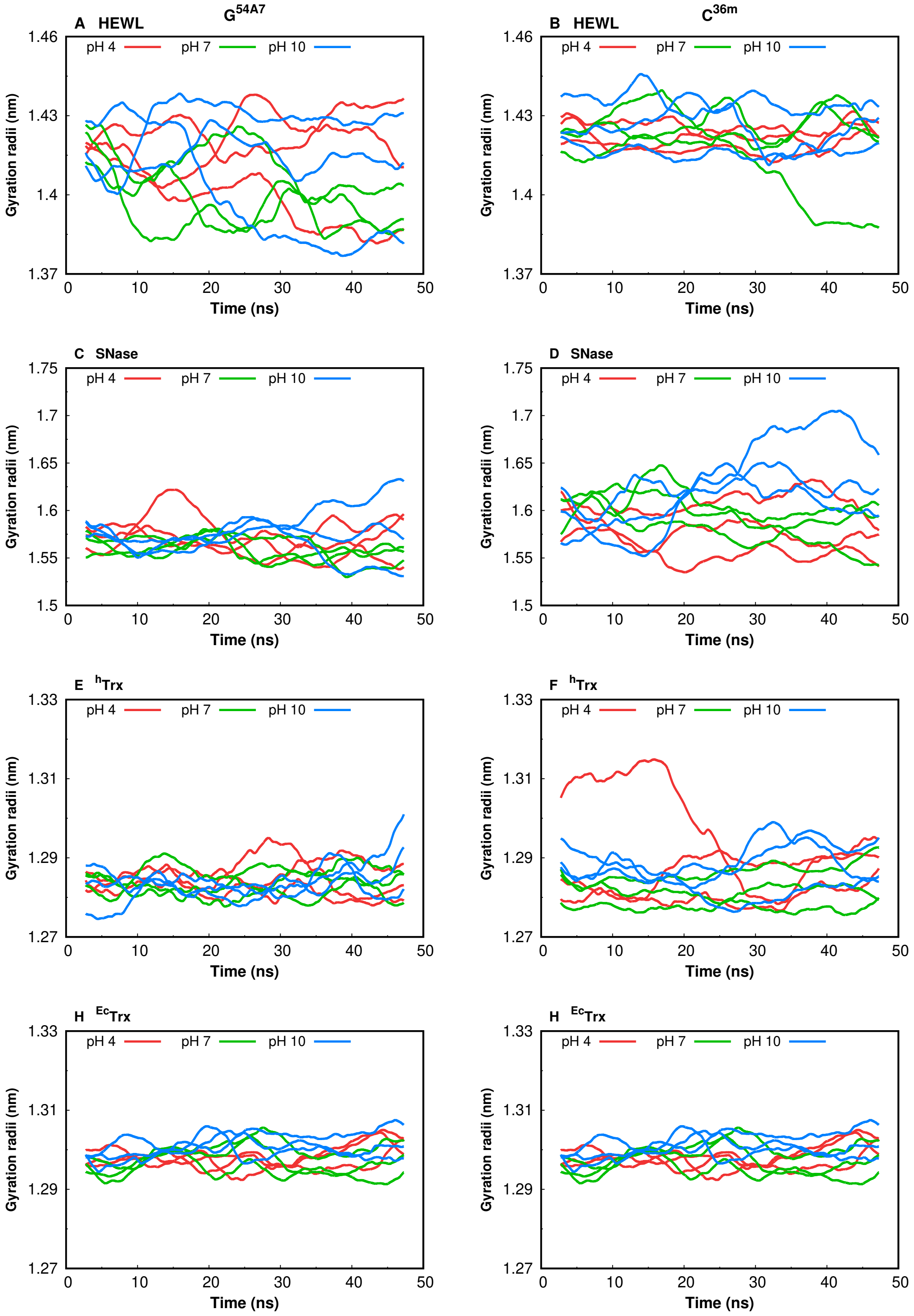

Figure S2: Gyration radii values over time of all simulated systems using G^{54A7} and C^{36m}. Triplicates of acidic (4), neutral and alkaline pH (10) simulations are represented. A sliding window average (5 ns) was applied to remove the undesired fast fluctuations.

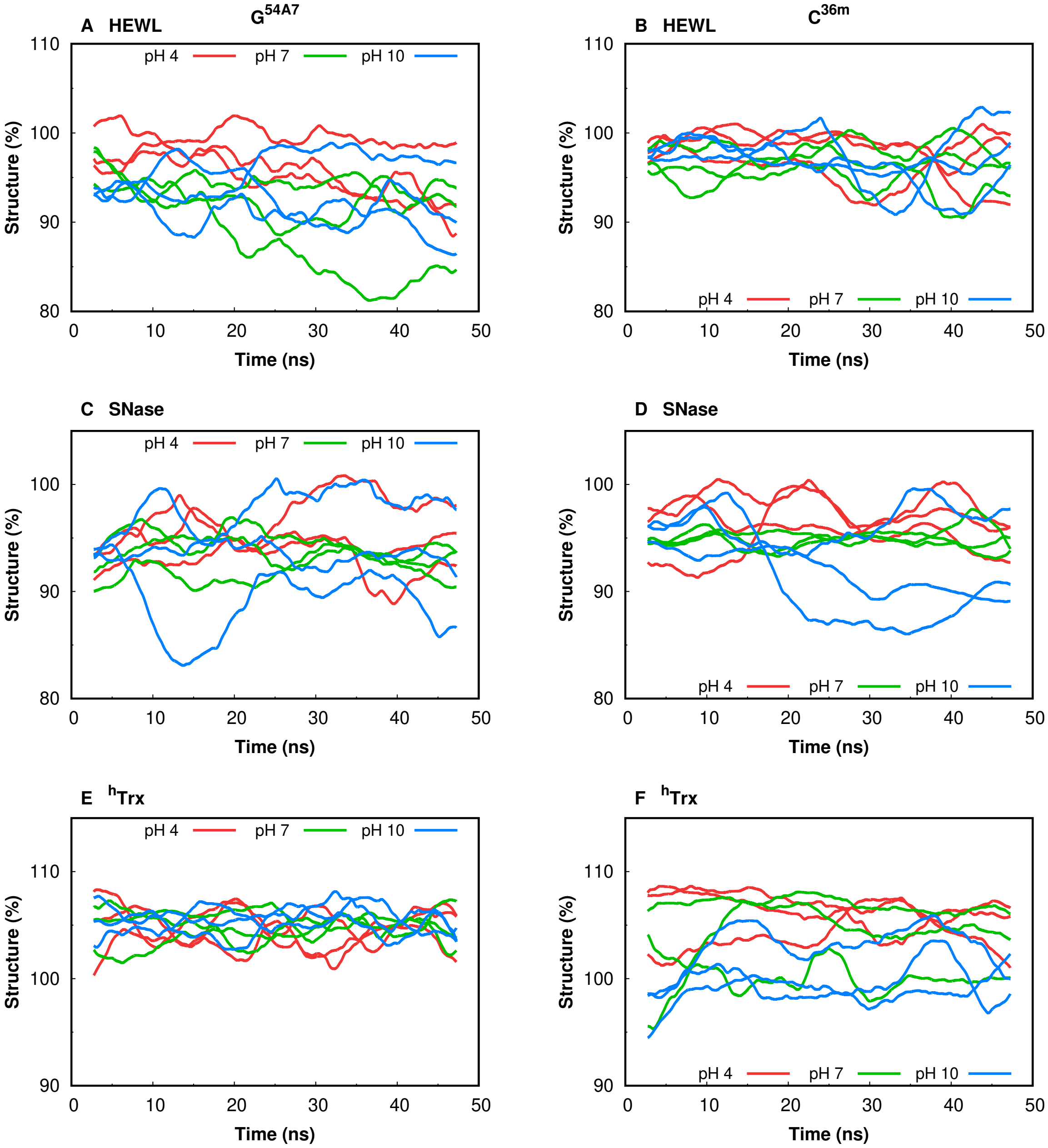
A HEWL G54A7
B HEWL C36m
C SNase
D SNase
E hTrx
F hTrx
pH 4
pH 7
pH 10
Structure (%)
Time (ns)

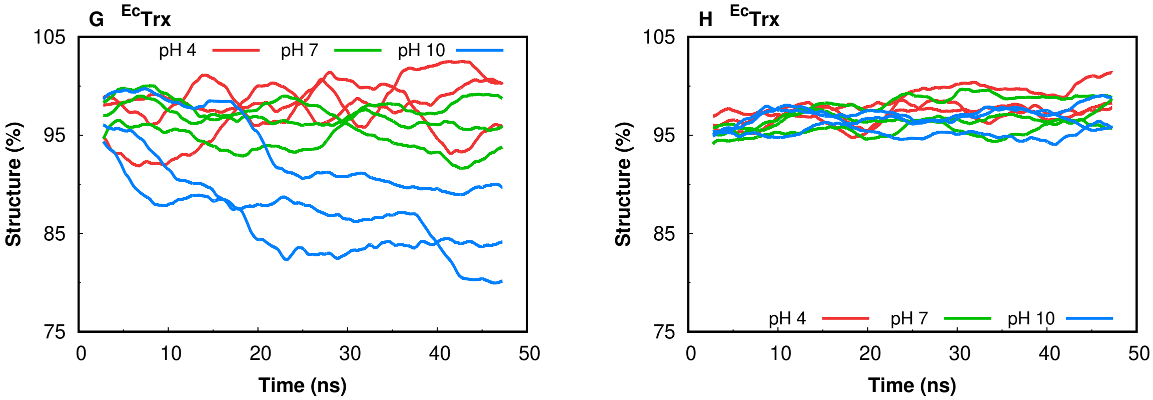

Figure S3: Secondary structure percentage over time of all simulated systems using G^{54A7} and C^{36m}. Triplicates of acidic (4), neutral and alkaline pH (10) simulations are represented. A sliding window average (5 ns) was applied to remove the undesired fast fluctuations.

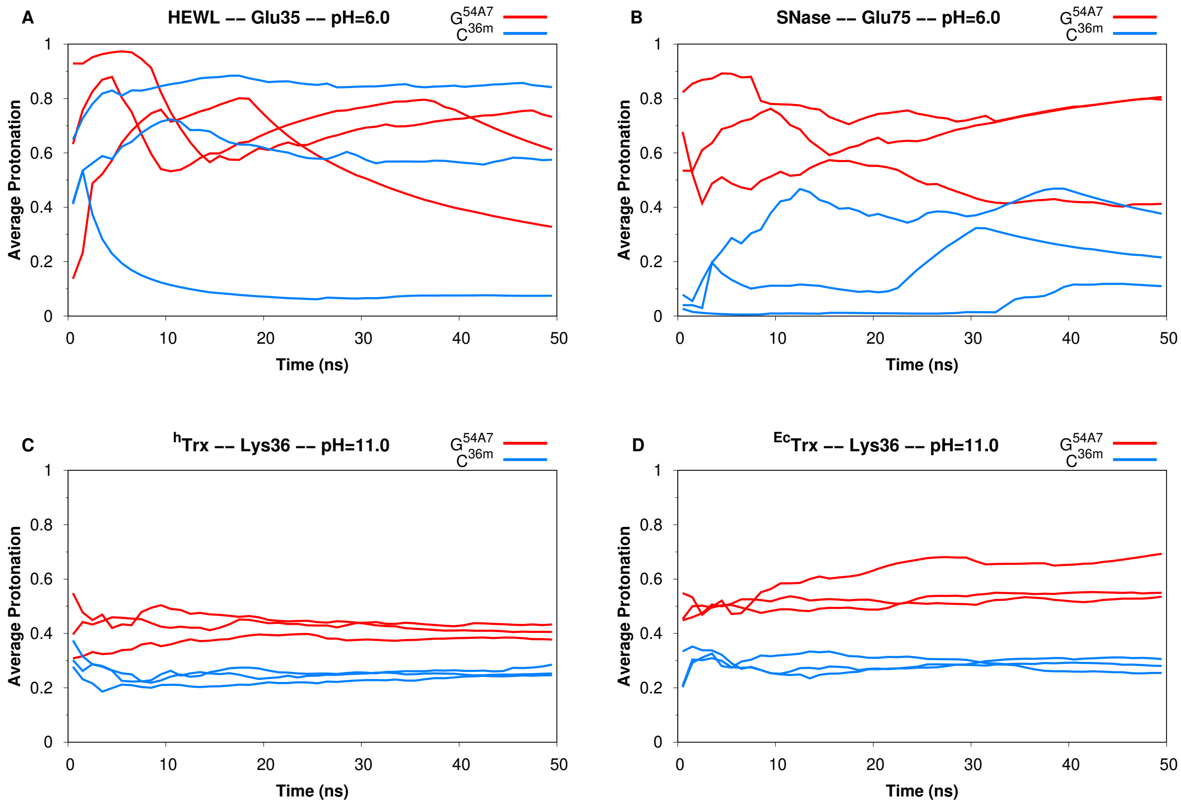

Figure S4: Accumulated average protonation over time of special residues for all simulated systems using G^{54A7} and C^{36m}. Triplicates of simulations with pH values closest to the respective estimated pK_a of each residue are represented.

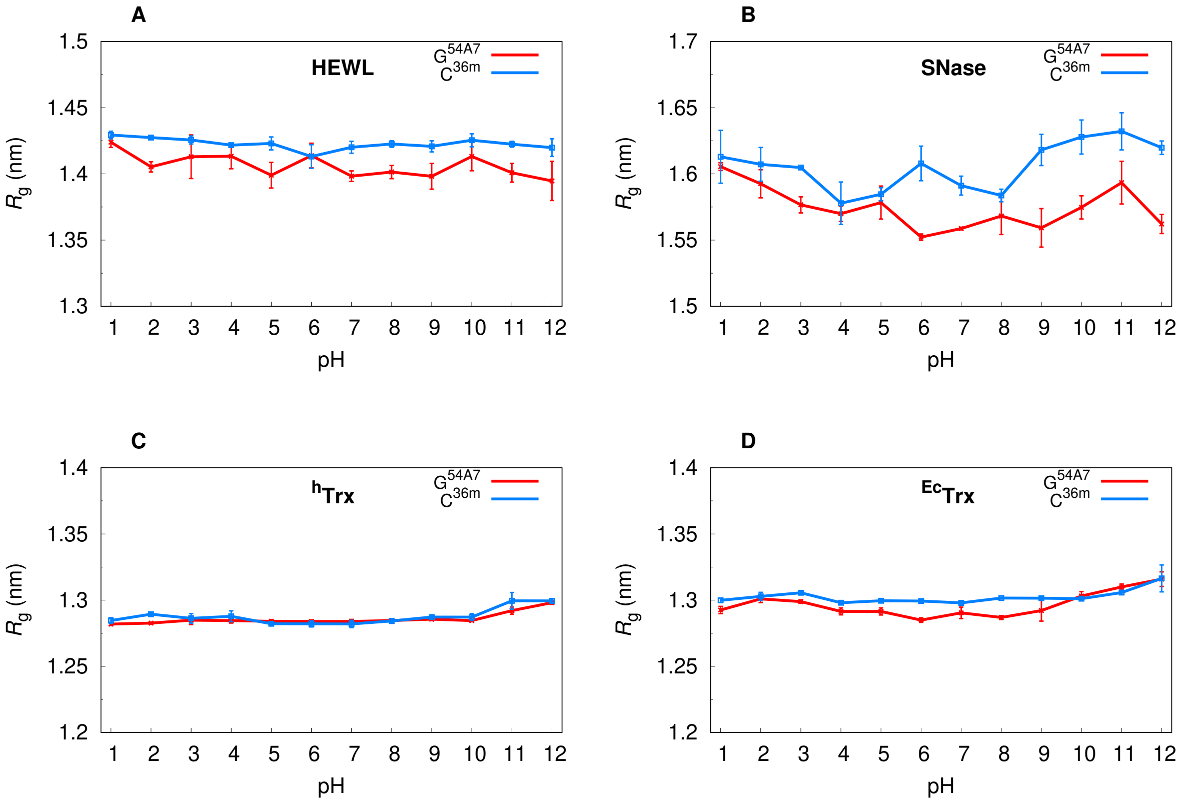

Figure S5: Average Gyration radii values for each pH. The CpHMD simulations using G^{54A7} (red) and C^{36m} (blue) force fields are shown. The error bars were calculated with the standard error of the mean.

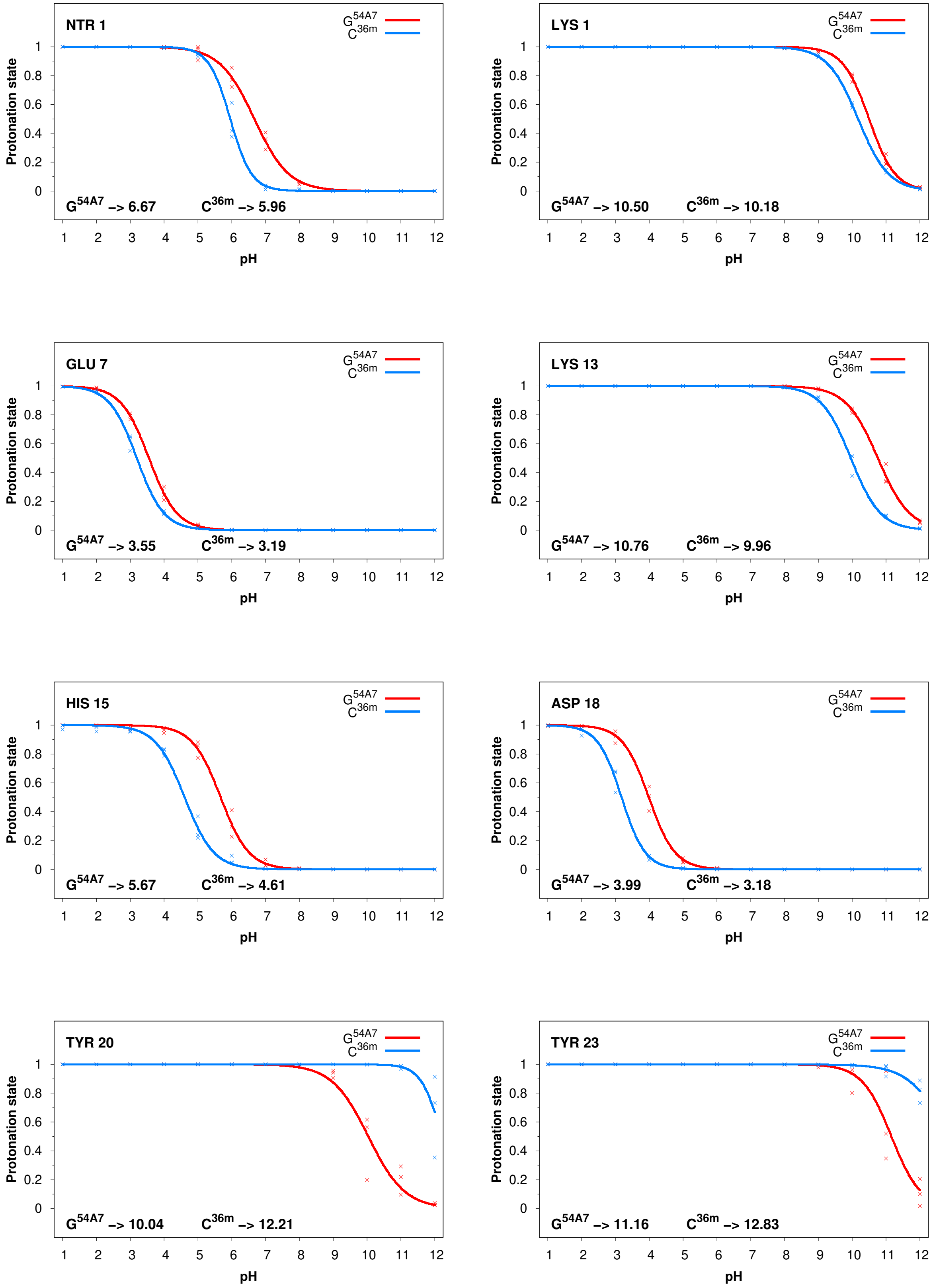
NTR 1
G^{54A7} –> 6.67
C^{36m} –> 5.96
LYS 1
G^{54A7} –> 10.50
C^{36m} –> 10.18
GLU 7
G^{54A7} –> 3.55
C^{36m} –> 3.19
LYS 13
G^{54A7} –> 10.76
C^{36m} –> 9.96
HIS 15
G^{54A7} –> 5.67
C^{36m} –> 4.61
ASP 18
G^{54A7} –> 3.99
C^{36m} –> 3.18
TYR 20
G^{54A7} –> 10.04
C^{36m} –> 12.21
TYR 23
G^{54A7} –> 11.16
C^{36m} –> 12.83
Protonation state
pH

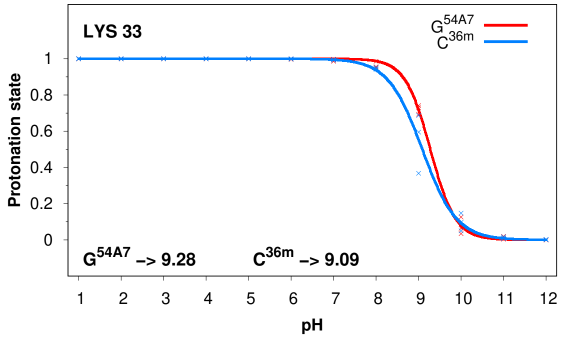
LYS 33
G54A7
C36m
G54A7 –> 9.28
C36m –> 9.09
Protonation state
pH

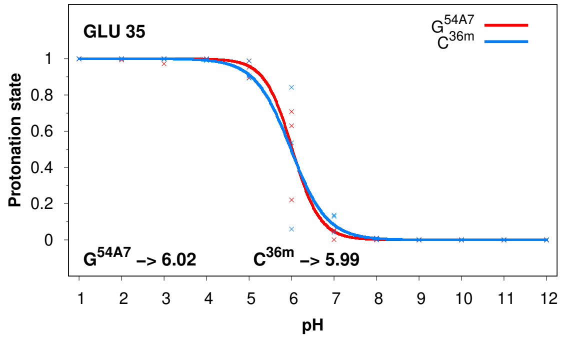
GLU 35
G54A7
C36m
G54A7 –> 6.02
C36m –> 5.99
Protonation state
pH

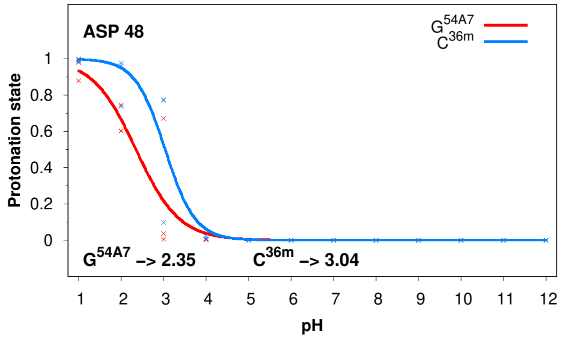
ASP 48
G54A7
C36m
G54A7 –> 2.35
C36m –> 3.04
Protonation state
pH

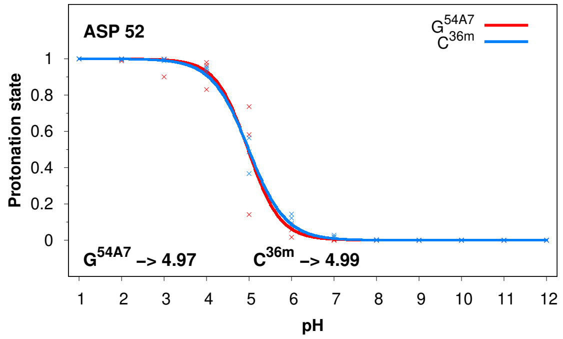
ASP 52
G54A7
C36m
G54A7 –> 4.97
C36m –> 4.99
Protonation state
pH

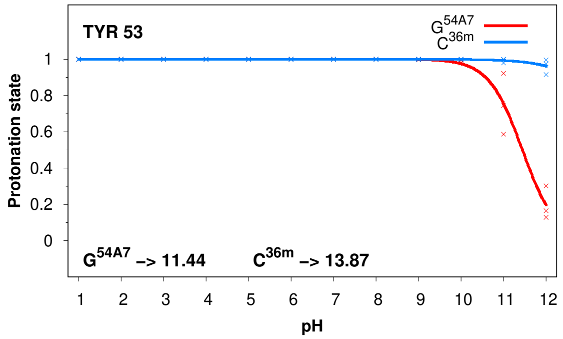
TYR 53
G54A7
C36m
G54A7 –> 11.44
C36m –> 13.87
Protonation state
pH

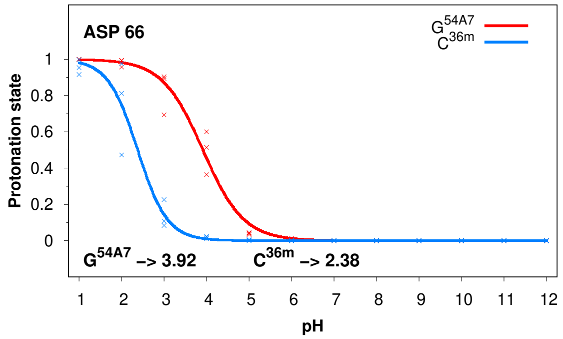
ASP 66
G54A7
C36m
G54A7 –> 3.92
C36m –> 2.38
Protonation state
pH

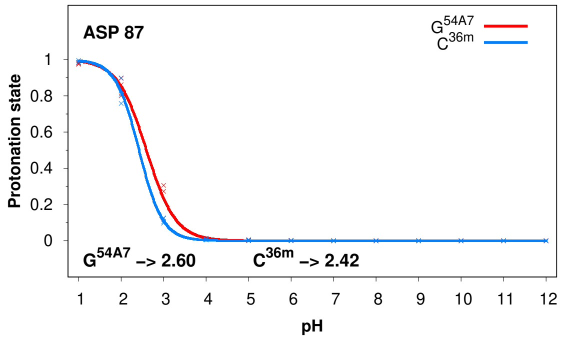
ASP 87
G54A7
C36m
G54A7 –> 2.60
C36m –> 2.42
Protonation state
pH

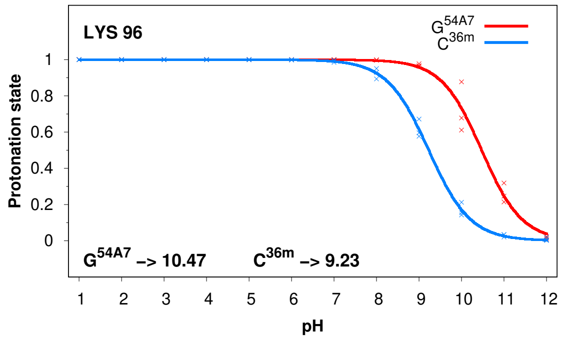
LYS 96
G54A7
C36m
G54A7 –> 10.47
C36m –> 9.23
Protonation state
pH

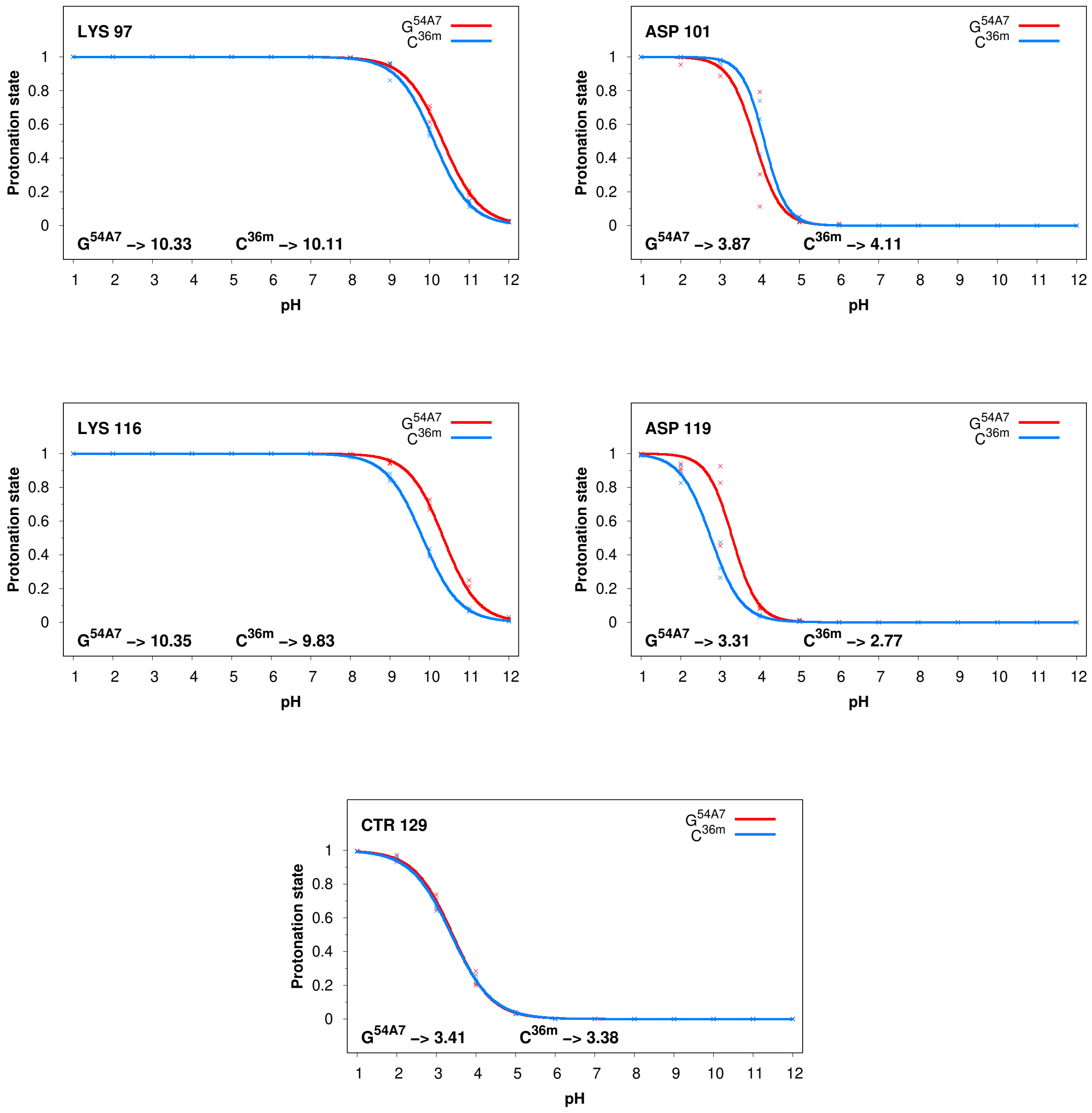

Figure S6: HEWL titration curves calculated for each residue. The average protonation state of each replicate, at each pH value, is represented by the colored dots.

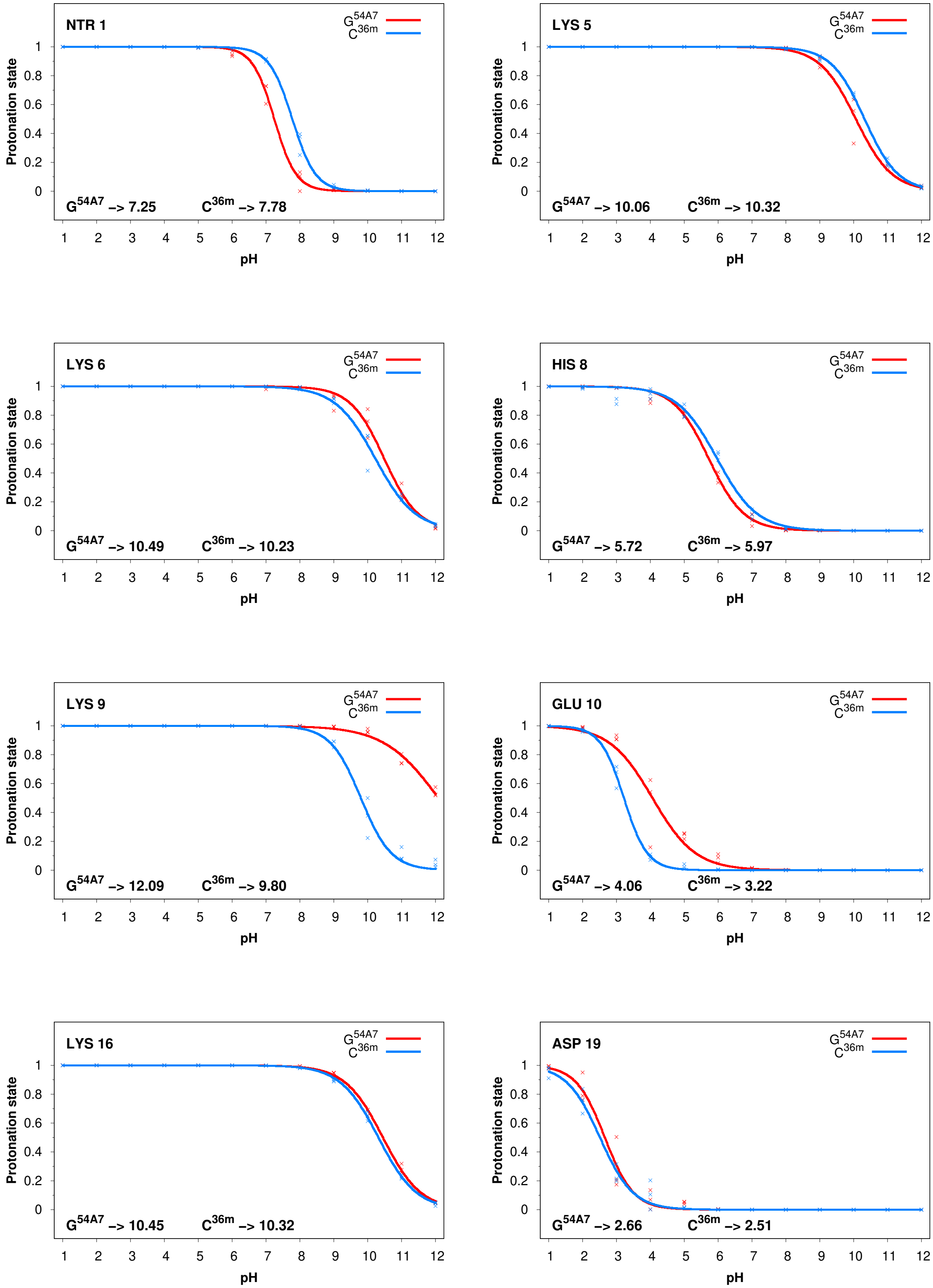
NTR 1
G54A7 –> 7.25
C36m –> 7.78
LYS 5
G54A7 –> 10.06
C36m –> 10.32
LYS 6
G54A7 –> 10.49
C36m –> 10.23
HIS 8
G54A7 –> 5.72
C36m –> 5.97
LYS 9
G54A7 –> 12.09
C36m –> 9.80
GLU 10
G54A7 –> 4.06
C36m –> 3.22
LYS 16
G54A7 –> 10.45
C36m –> 10.32
ASP 19
G54A7 –> 2.66
C36m –> 2.51
Protonation state
pH

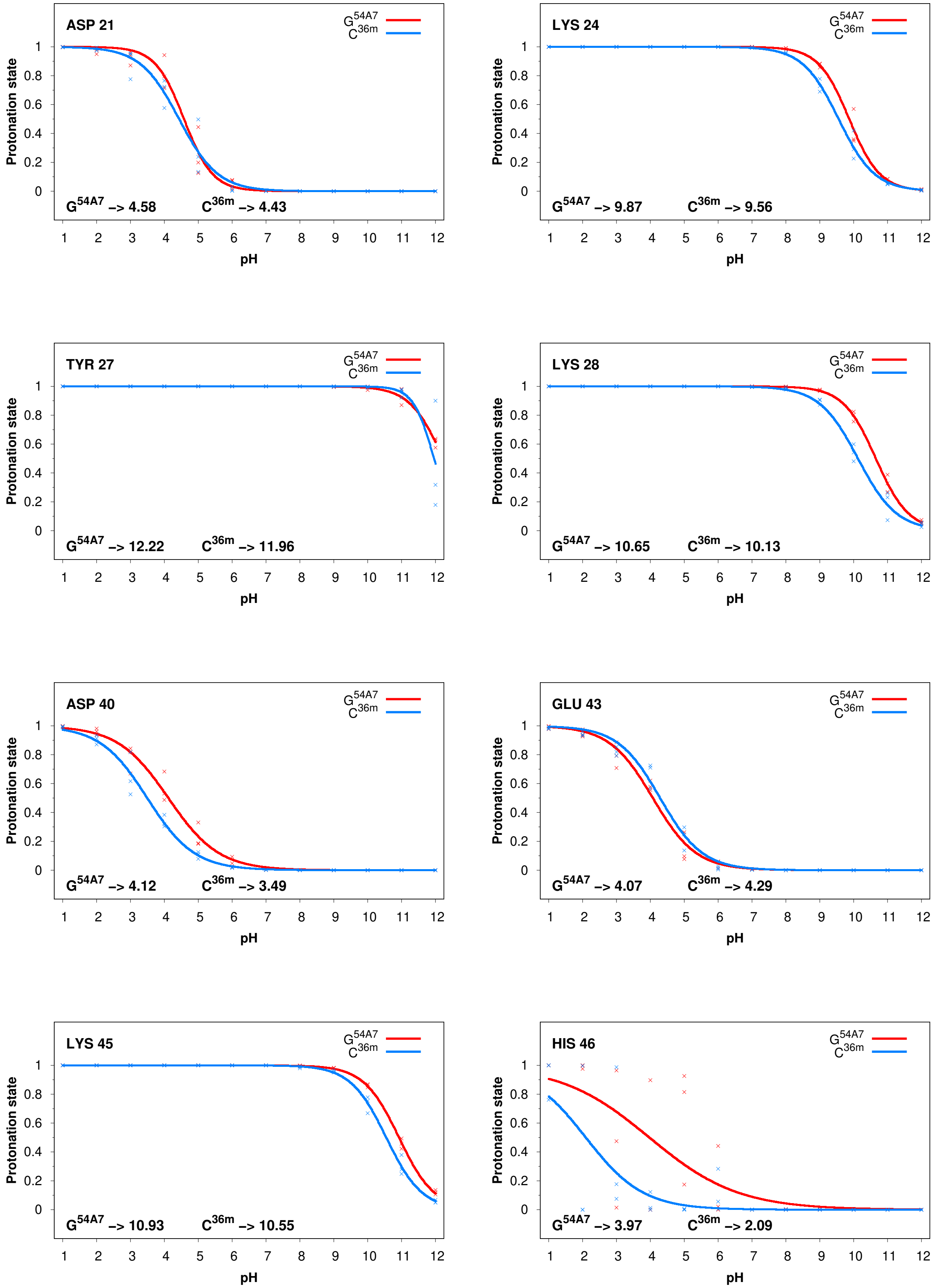

ASP 21
G54A7 -> 4.58
C36m -> 4.43
LYS 24
G54A7 -> 9.87
C36m -> 9.56
TYR 27
G54A7 -> 12.22
C36m -> 11.96
LYS 28
G54A7 -> 10.65
C36m -> 10.13
ASP 40
G54A7 -> 4.12
C36m -> 3.49
GLU 43
G54A7 -> 4.07
C36m -> 4.29
LYS 45
G54A7 -> 10.93
C36m -> 10.55
HIS 46
G54A7 -> 3.97
C36m -> 2.09
G54A7
C36m
Protonation state
pH

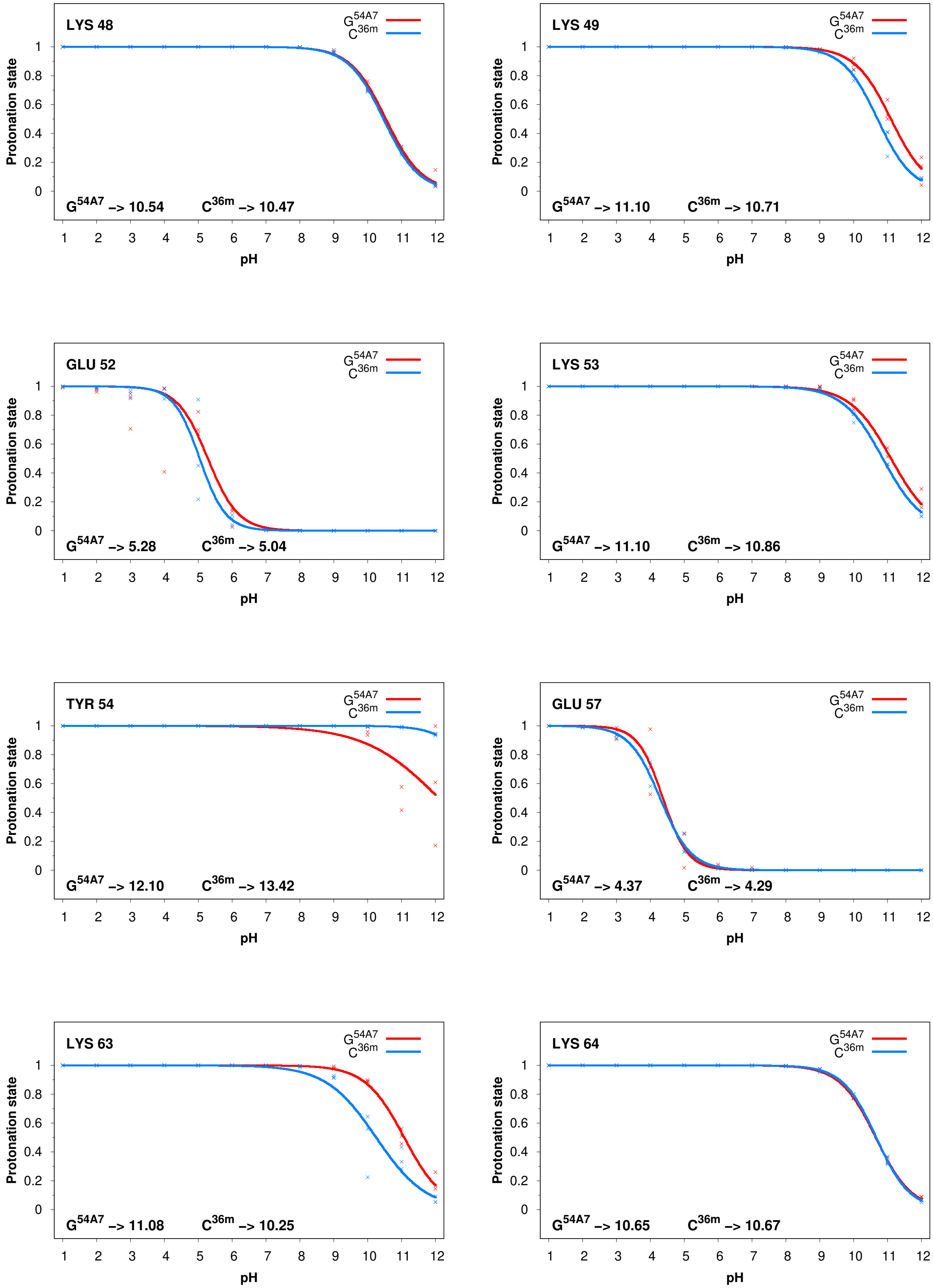
LYS 48
G54A7 –> 10.54
C36m –> 10.47
LYS 49
G54A7 –> 11.10
C36m –> 10.71
GLU 52
G54A7 –> 5.28
C36m –> 5.04
LYS 53
G54A7 –> 11.10
C36m –> 10.86
TYR 54
G54A7 –> 12.10
C36m –> 13.42
GLU 57
G54A7 –> 4.37
C36m –> 4.29
LYS 63
G54A7 –> 11.08
C36m –> 10.25
LYS 64
G54A7 –> 10.65
C36m –> 10.67
G54A7
C36m
Protonation state
pH

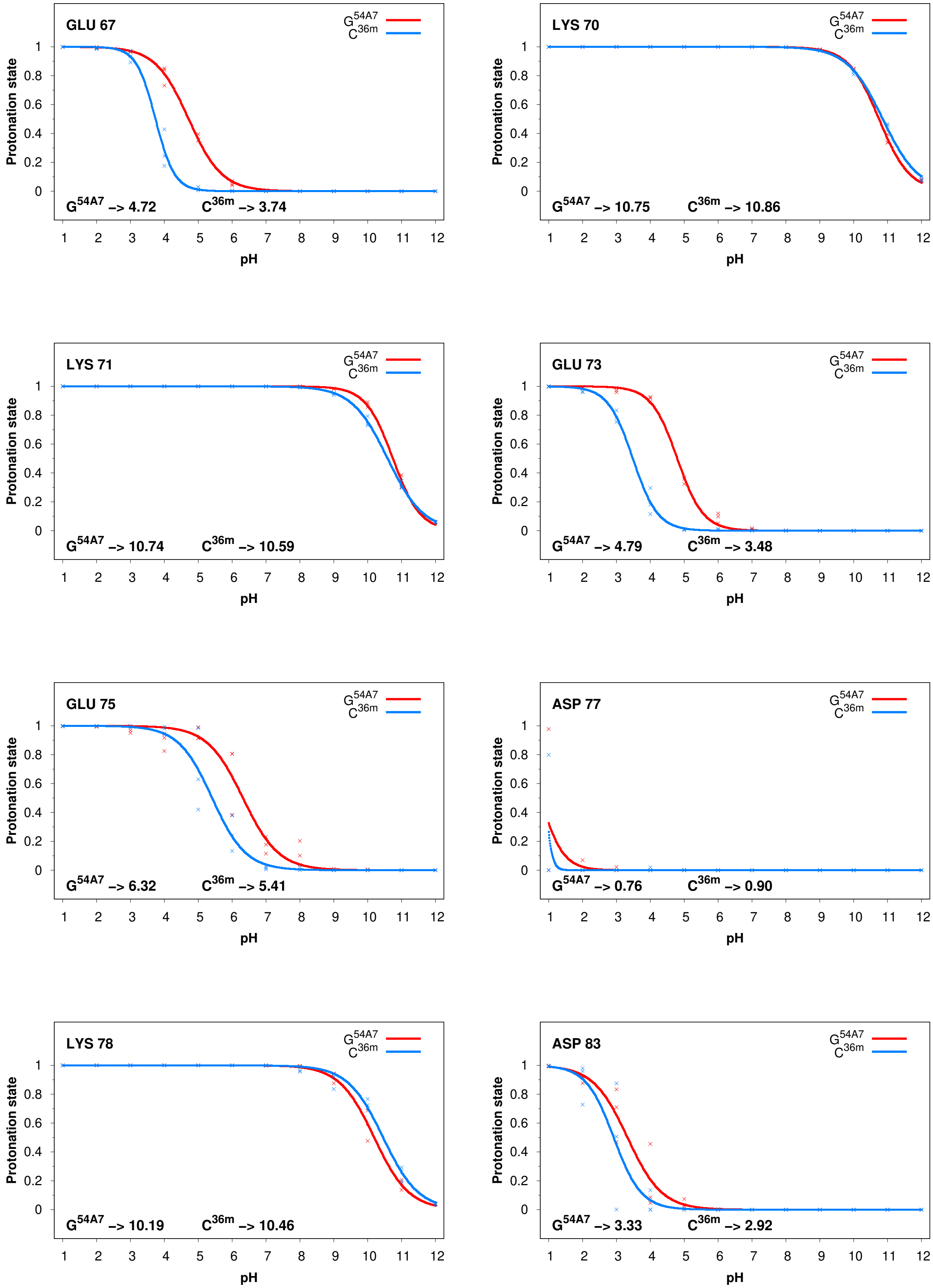
GLU 67
G^{54A7} –> 4.72
C^{36m} –> 3.74
LYS 70
G^{54A7} –> 10.75
C^{36m} –> 10.86
LYS 71
G^{54A7} –> 10.74
C^{36m} –> 10.59
GLU 73
G^{54A7} –> 4.79
C^{36m} –> 3.48
GLU 75
G^{54A7} –> 6.32
C^{36m} –> 5.41
ASP 77
G^{54A7} –> 0.76
C^{36m} –> 0.90
LYS 78
G^{54A7} –> 10.19
C^{36m} –> 10.46
ASP 83
G^{54A7} –> 3.33
C^{36m} –> 2.92
Protonation state
pH

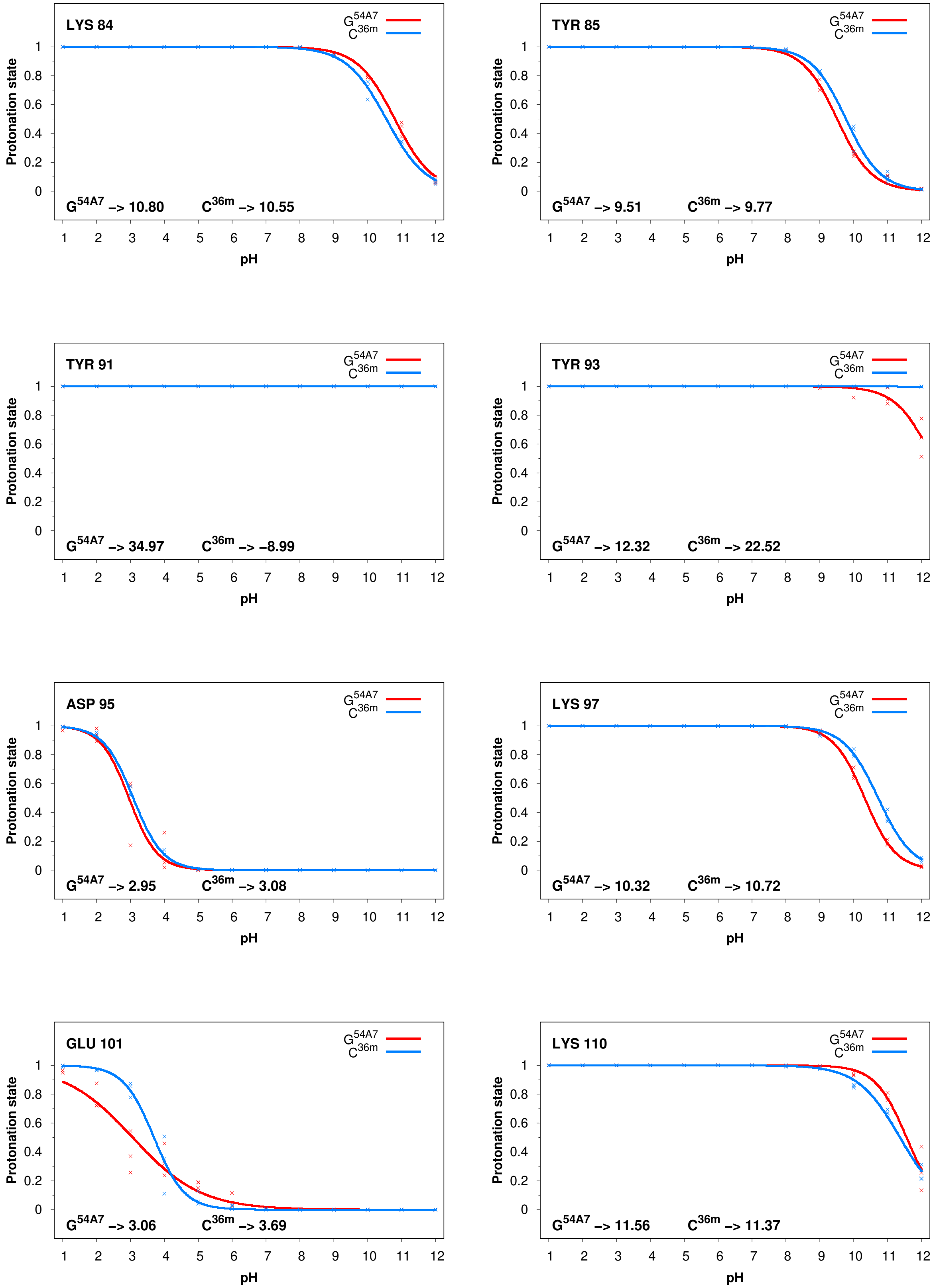
LYS 84
G^54A7 –> 10.80
C^36m –> 10.55
TYR 85
G^54A7 –> 9.51
C^36m –> 9.77
TYR 91
G^54A7 –> 34.97
C^36m –> –8.99
TYR 93
G^54A7 –> 12.32
C^36m –> 22.52
ASP 95
G^54A7 –> 2.95
C^36m –> 3.08
LYS 97
G^54A7 –> 10.32
C^36m –> 10.72
GLU 101
G^54A7 –> 3.06
C^36m –> 3.69
LYS 110
G^54A7 –> 11.56
C^36m –> 11.37
Protonation state
pH

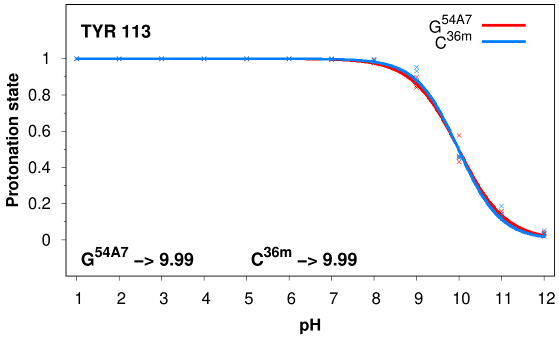
TYR 113
G54A7
C36m
Protonation state
pH
G54A7 –> 9.99
C36m –> 9.99

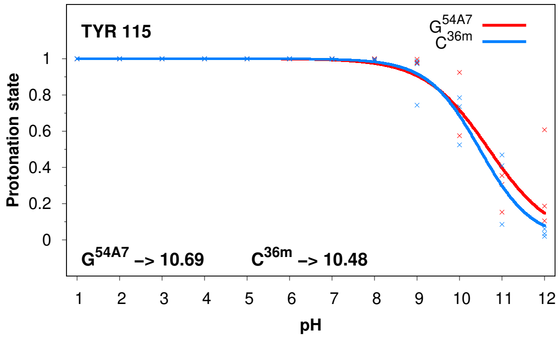
TYR 115
G54A7
C36m
Protonation state
pH
G54A7 –> 10.69
C36m –> 10.48

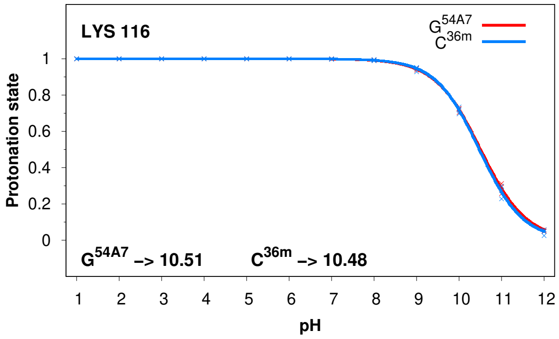
LYS 116
G54A7
C36m
Protonation state
pH
G54A7 –> 10.51
C36m –> 10.48

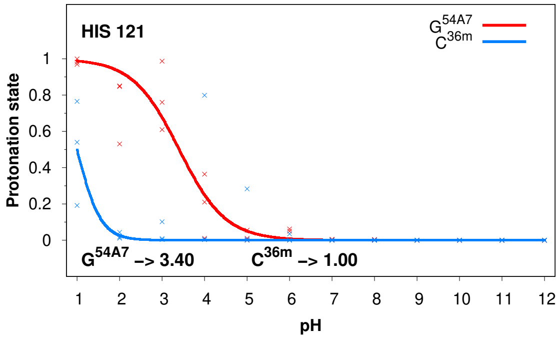
HIS 121
G54A7
C36m
Protonation state
pH
G54A7 –> 3.40
C36m –> 1.00

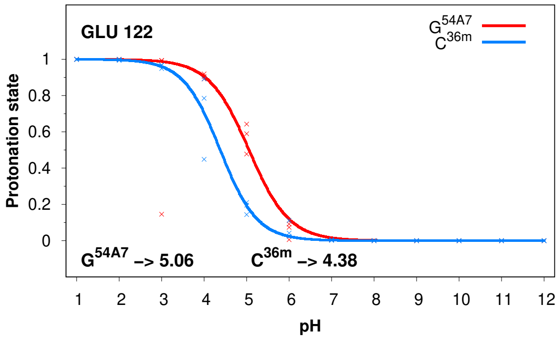
GLU 122
G54A7
C36m
Protonation state
pH
G54A7 –> 5.06
C36m –> 4.38

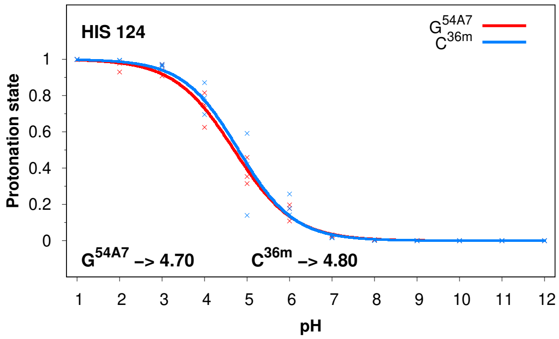
HIS 124
G54A7
C36m
Protonation state
pH
G54A7 –> 4.70
C36m –> 4.80

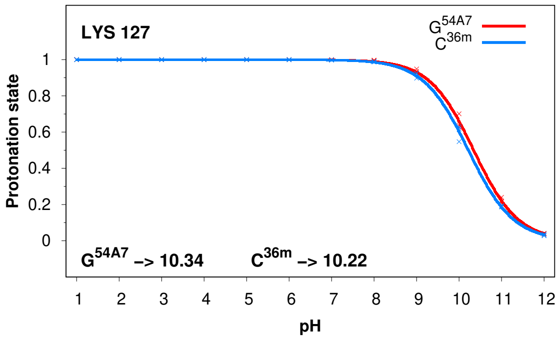
LYS 127
G54A7
C36m
Protonation state
pH
G54A7 –> 10.34
C36m –> 10.22

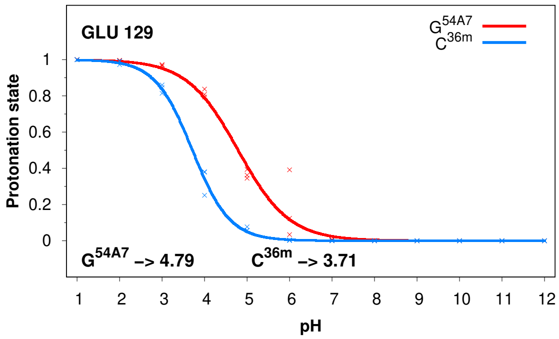
GLU 129
G54A7
C36m
Protonation state
pH
G54A7 –> 4.79
C36m –> 3.71

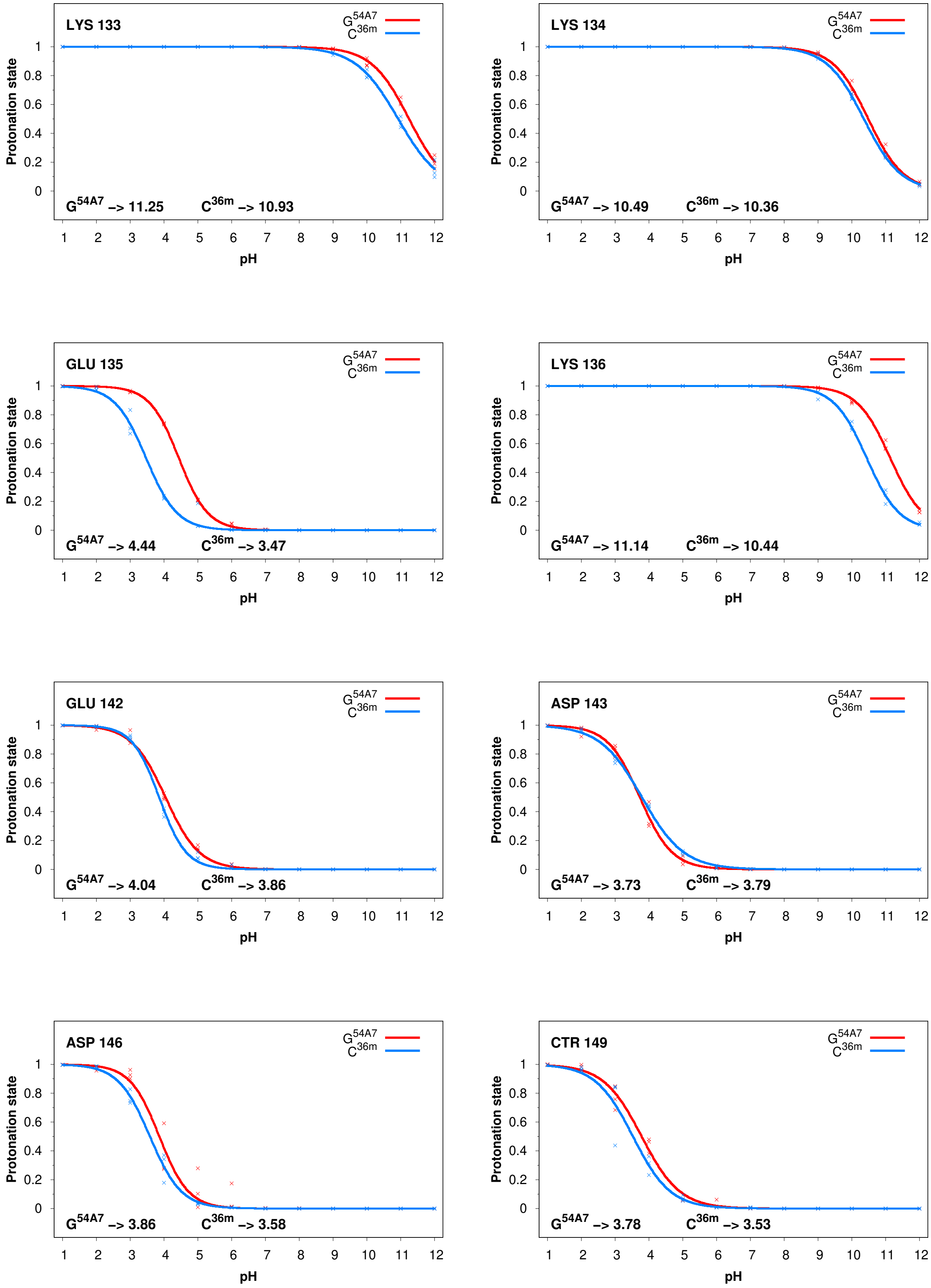

Figure S7: SNase titration curves calculated for each residue. The average protonation state of each replicate, at each pH value, is represented by the colored dots.

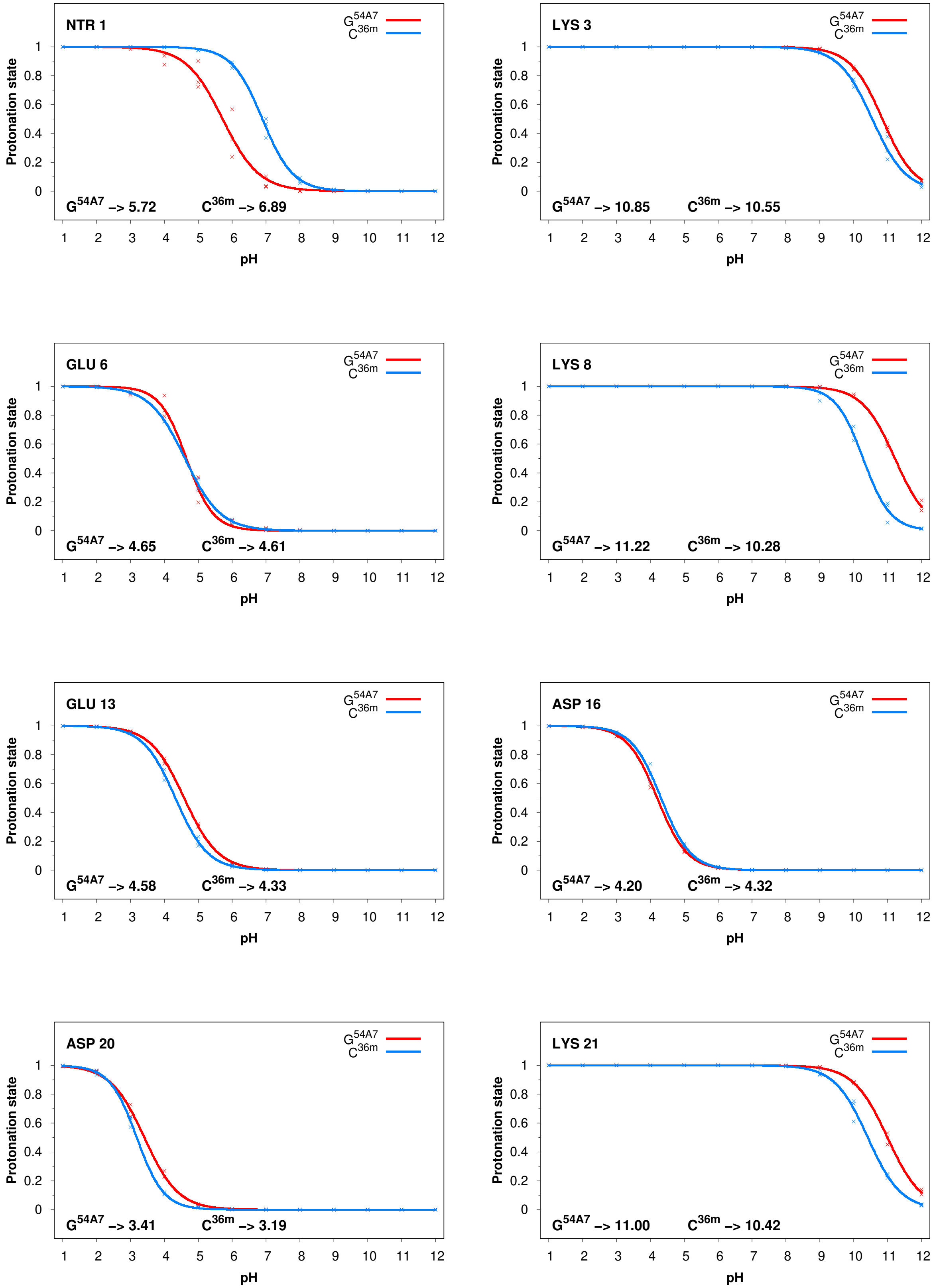
NTR 1
G54A7
C36m
G54A7 –> 5.72
C36m –> 6.89
LYS 3
G54A7 –> 10.85
C36m –> 10.55
GLU 6
G54A7 –> 4.65
C36m –> 4.61
LYS 8
G54A7 –> 11.22
C36m –> 10.28
GLU 13
G54A7 –> 4.58
C36m –> 4.33
ASP 16
G54A7 –> 4.20
C36m –> 4.32
ASP 20
G54A7 –> 3.41
C36m –> 3.19
LYS 21
G54A7 –> 11.00
C36m –> 10.42
Protonation state
pH

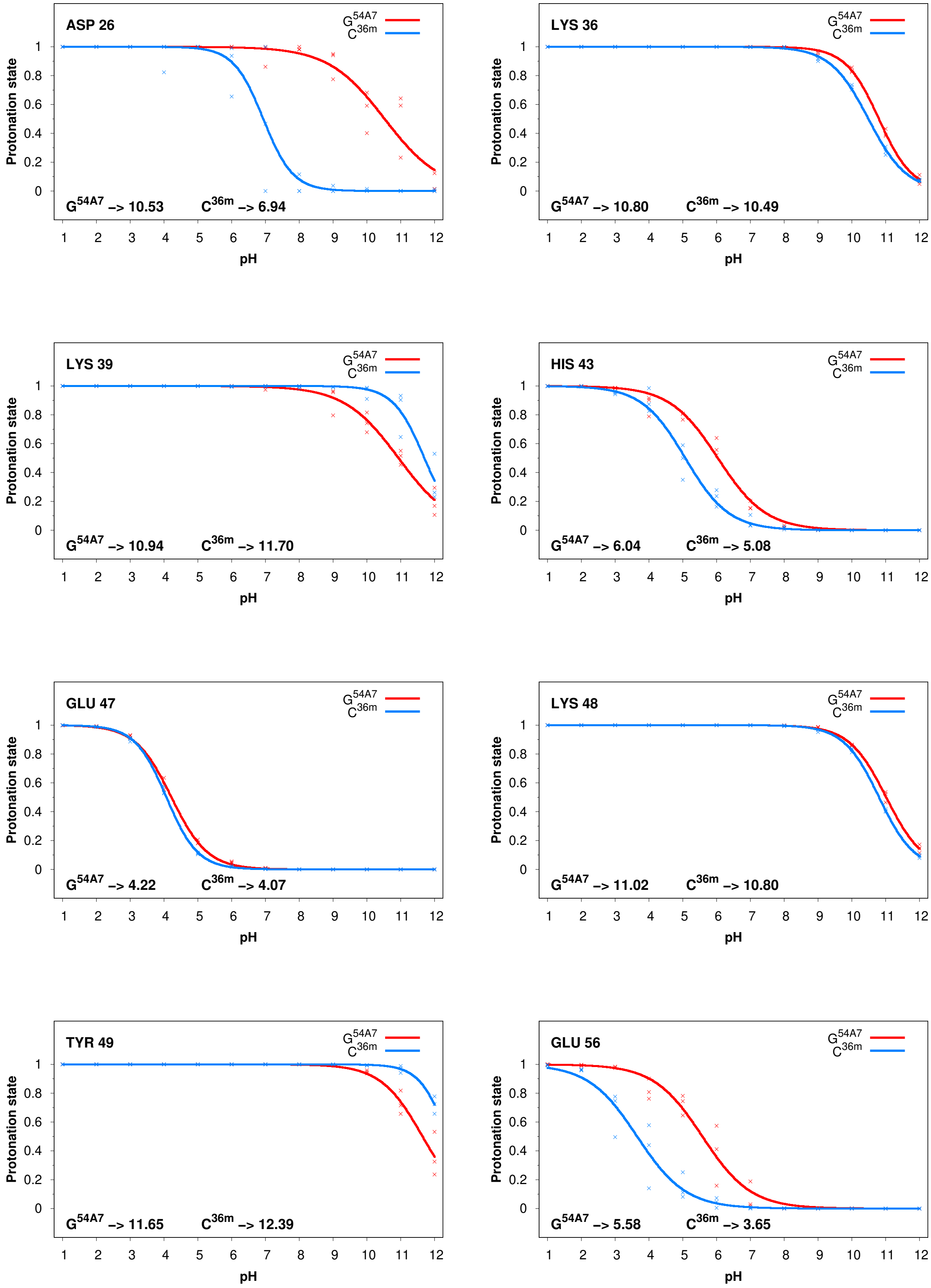
ASP 26
G54A7 –> 10.53
C36m –> 6.94
LYS 36
G54A7 –> 10.80
C36m –> 10.49
LYS 39
G54A7 –> 10.94
C36m –> 11.70
HIS 43
G54A7 –> 6.04
C36m –> 5.08
GLU 47
G54A7 –> 4.22
C36m –> 4.07
LYS 48
G54A7 –> 11.02
C36m –> 10.80
TYR 49
G54A7 –> 11.65
C36m –> 12.39
GLU 56
G54A7 –> 5.58
C36m –> 3.65
G54A7
C36m
Protonation state
pH

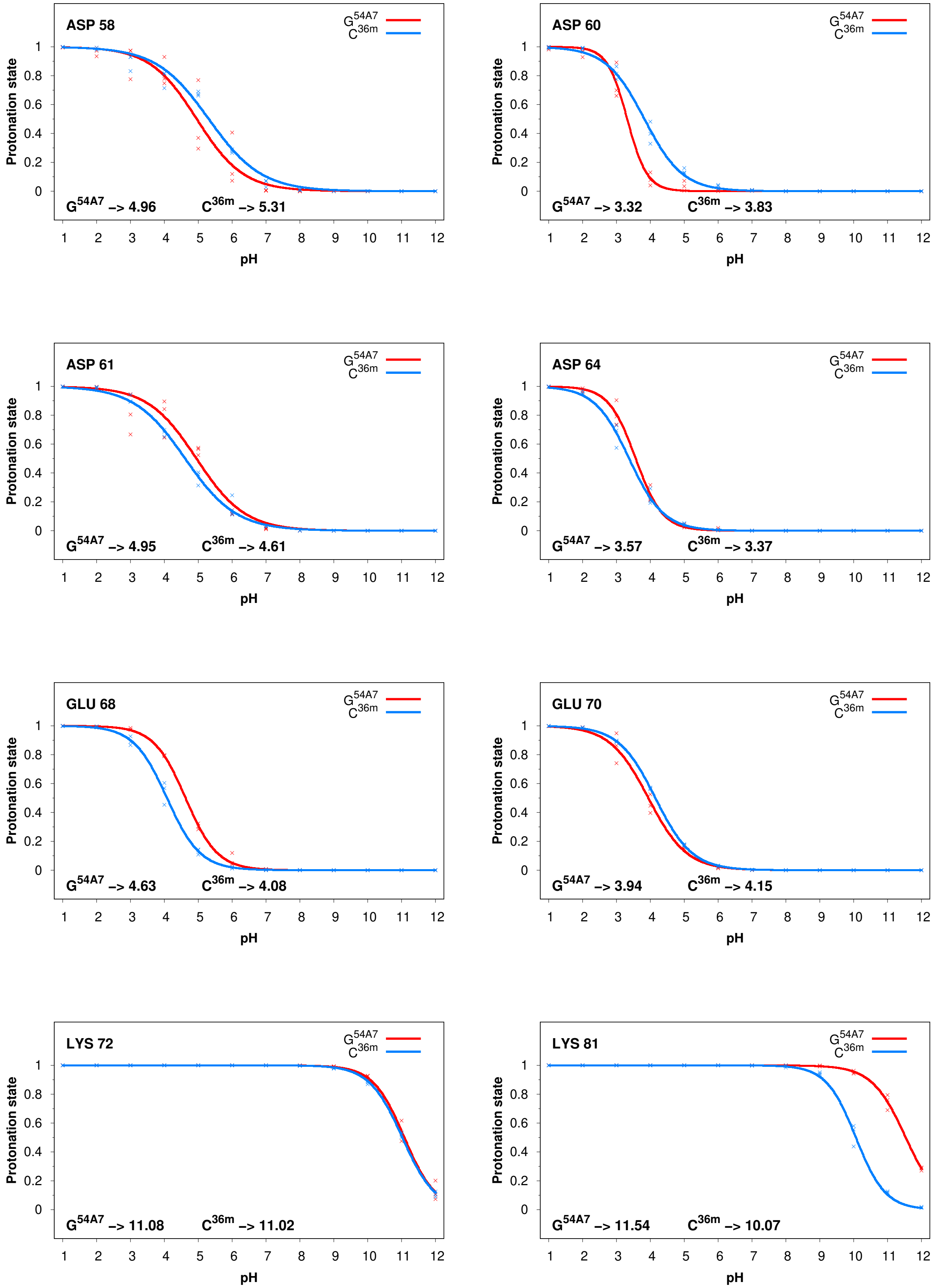
ASP 58
G54A7 –> 4.96
C36m –> 5.31
ASP 60
G54A7 –> 3.32
C36m –> 3.83
ASP 61
G54A7 –> 4.95
C36m –> 4.61
ASP 64
G54A7 –> 3.57
C36m –> 3.37
GLU 68
G54A7 –> 4.63
C36m –> 4.08
GLU 70
G54A7 –> 3.94
C36m –> 4.15
LYS 72
G54A7 –> 11.08
C36m –> 11.02
LYS 81
G54A7 –> 11.54
C36m –> 10.07
Protonation state
pH

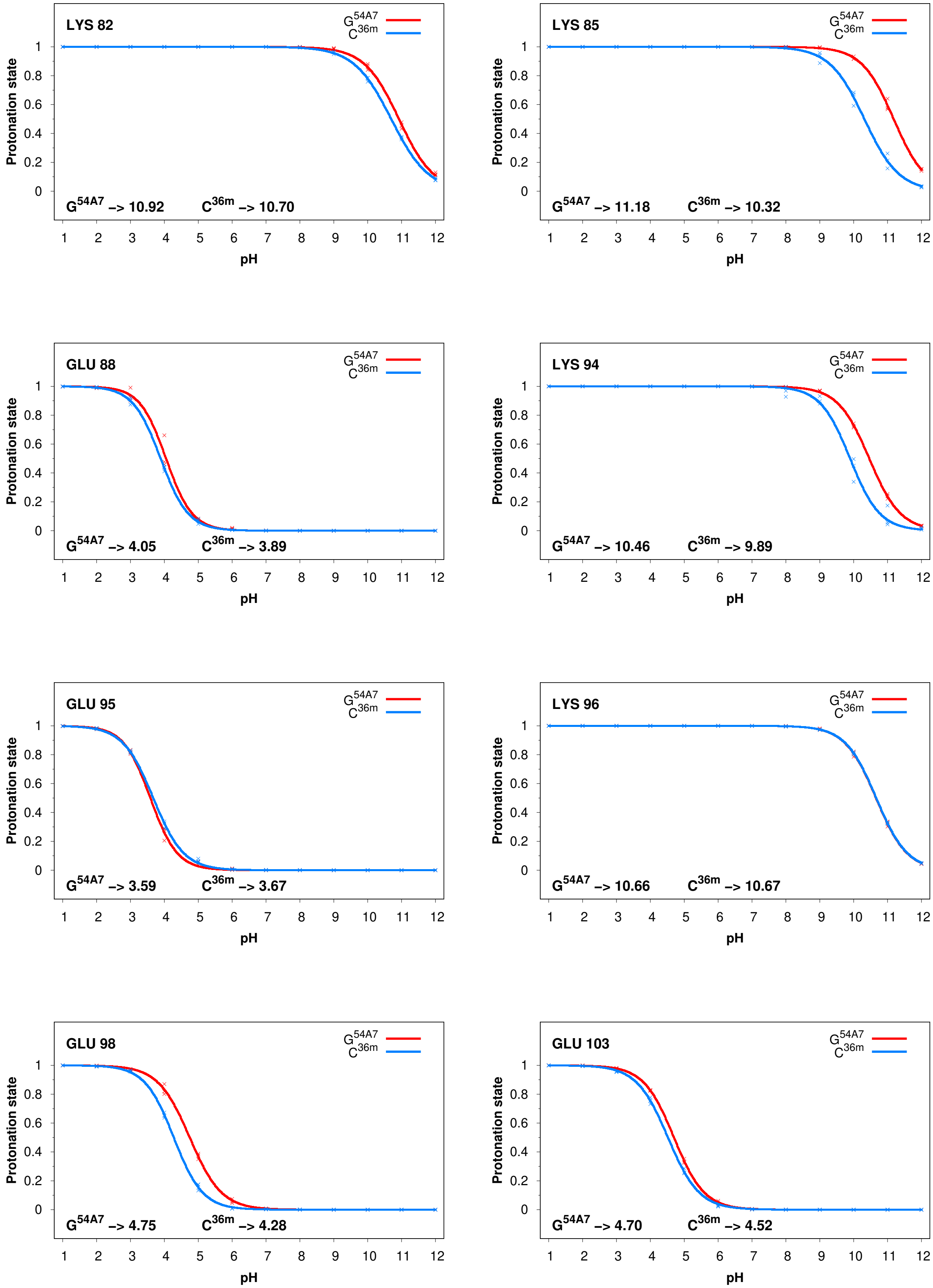
LYS 82
G54A7 –> 10.92 C36m –> 10.70
LYS 85
G54A7 –> 11.18 C36m –> 10.32
GLU 88
G54A7 –> 4.05 C36m –> 3.89
LYS 94
G54A7 –> 10.46 C36m –> 9.89
GLU 95
G54A7 –> 3.59 C36m –> 3.67
LYS 96
G54A7 –> 10.66 C36m –> 10.67
GLU 98
G54A7 –> 4.75 C36m –> 4.28
GLU 103
G54A7 –> 4.70 C36m –> 4.52
Protonation state
pH

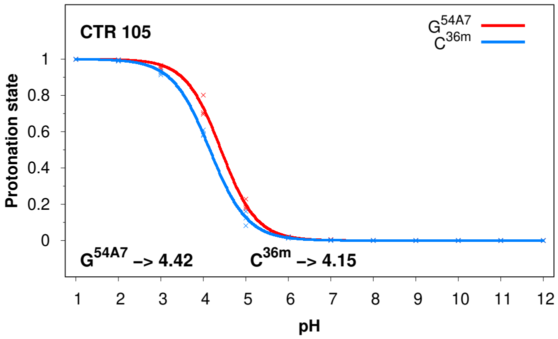

Figure S8: ^{h}Trx titration curves calculated for each residue. The average protonation state of each replicate, at each pH value, is represented by the colored dots.

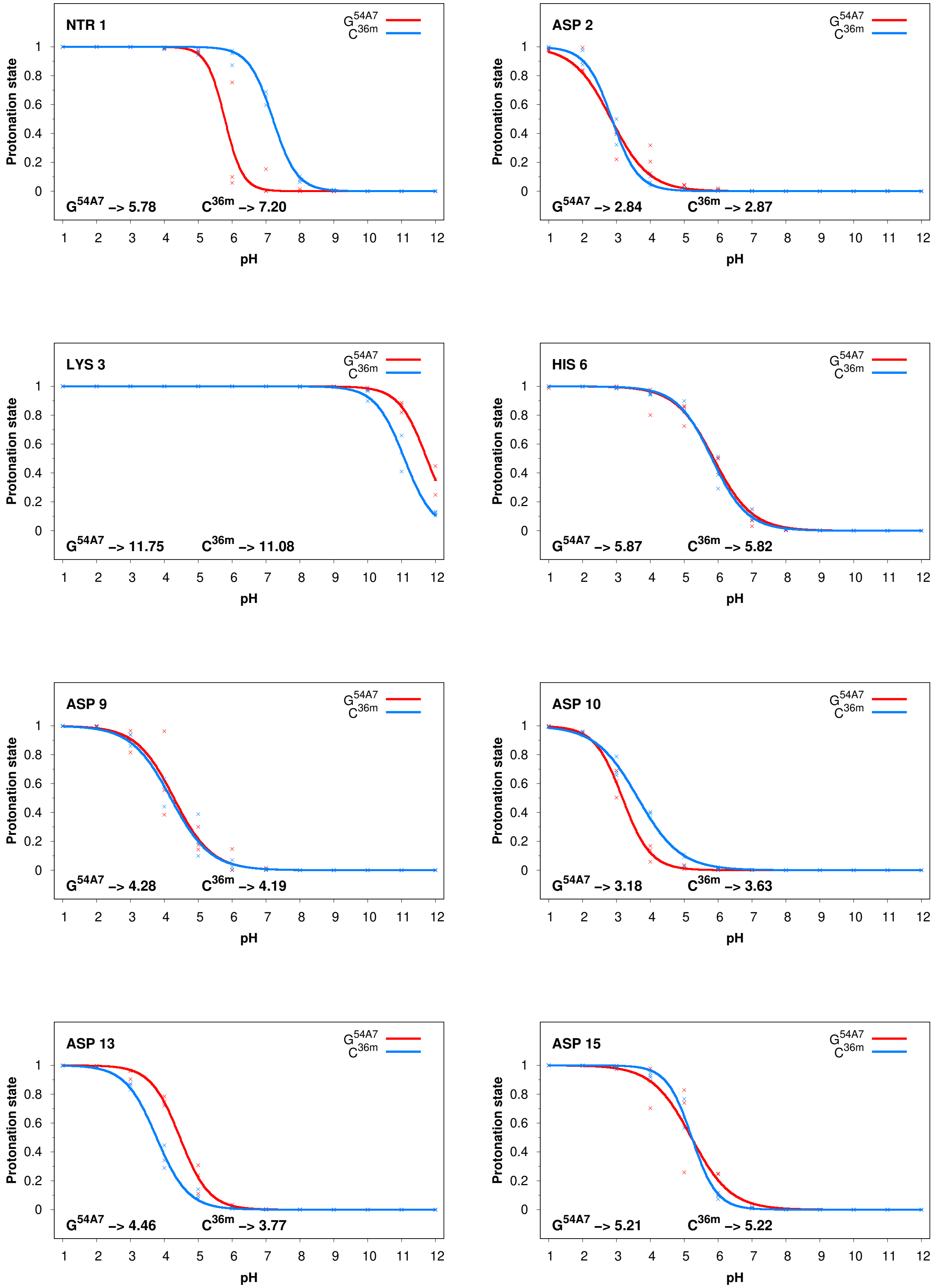
NTR 1
G^{54A7} –> 5.78
C^{36m} –> 7.20
ASP 2
G^{54A7} –> 2.84
C^{36m} –> 2.87
LYS 3
G^{54A7} –> 11.75
C^{36m} –> 11.08
HIS 6
G^{54A7} –> 5.87
C^{36m} –> 5.82
ASP 9
G^{54A7} –> 4.28
C^{36m} –> 4.19
ASP 10
G^{54A7} –> 3.18
C^{36m} –> 3.63
ASP 13
G^{54A7} –> 4.46
C^{36m} –> 3.77
ASP 15
G^{54A7} –> 5.21
C^{36m} –> 5.22
G^{54A7}
C^{36m}
Protonation state
pH

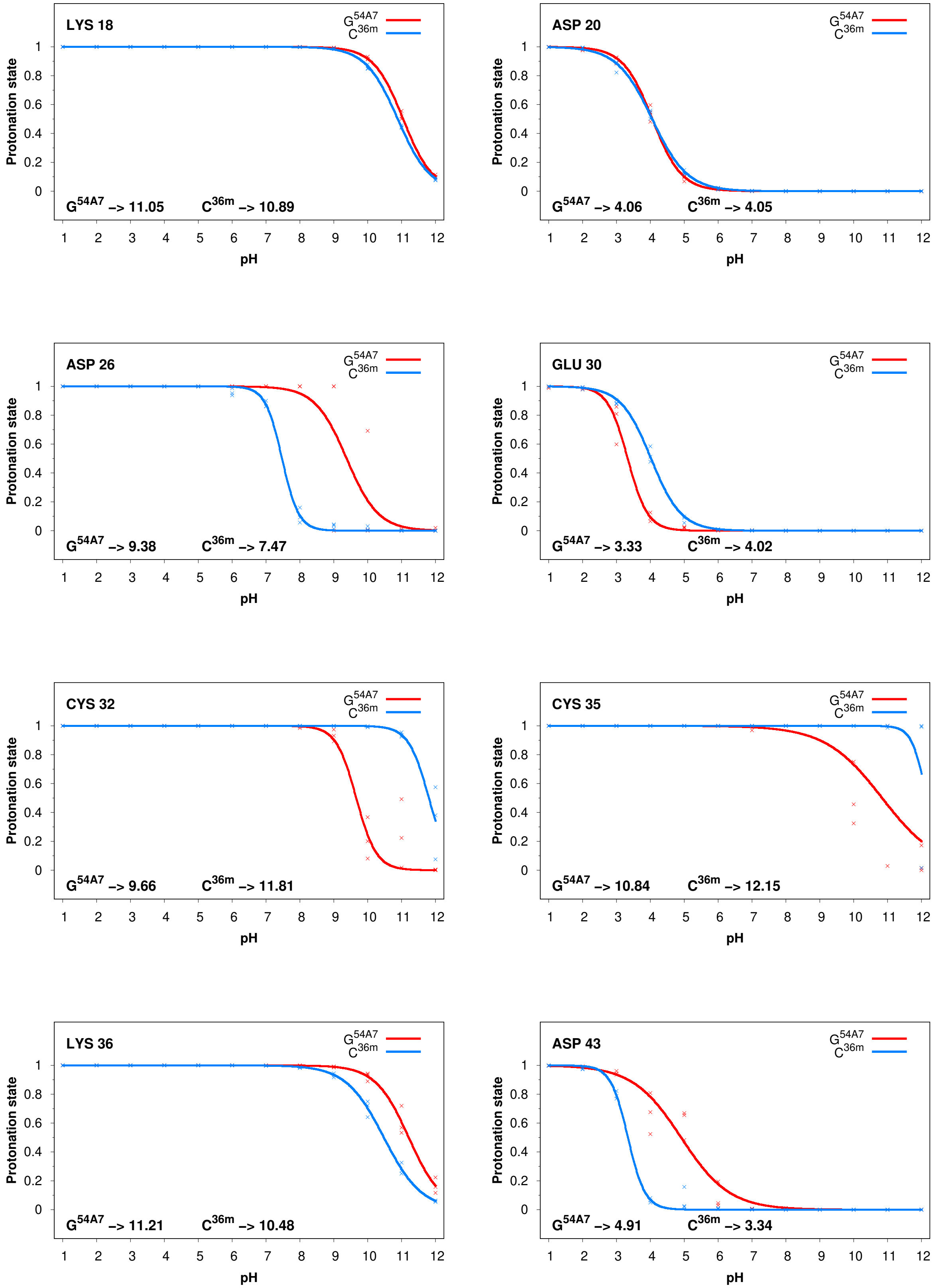
LYS 18
G54A7 -> 11.05
C36m -> 10.89
ASP 20
G54A7 -> 4.06
C36m -> 4.05
ASP 26
G54A7 -> 9.38
C36m -> 7.47
GLU 30
G54A7 -> 3.33
C36m -> 4.02
CYS 32
G54A7 -> 9.66
C36m -> 11.81
CYS 35
G54A7 -> 10.84
C36m -> 12.15
LYS 36
G54A7 -> 11.21
C36m -> 10.48
ASP 43
G54A7 -> 4.91
C36m -> 3.34
Protonation state
pH

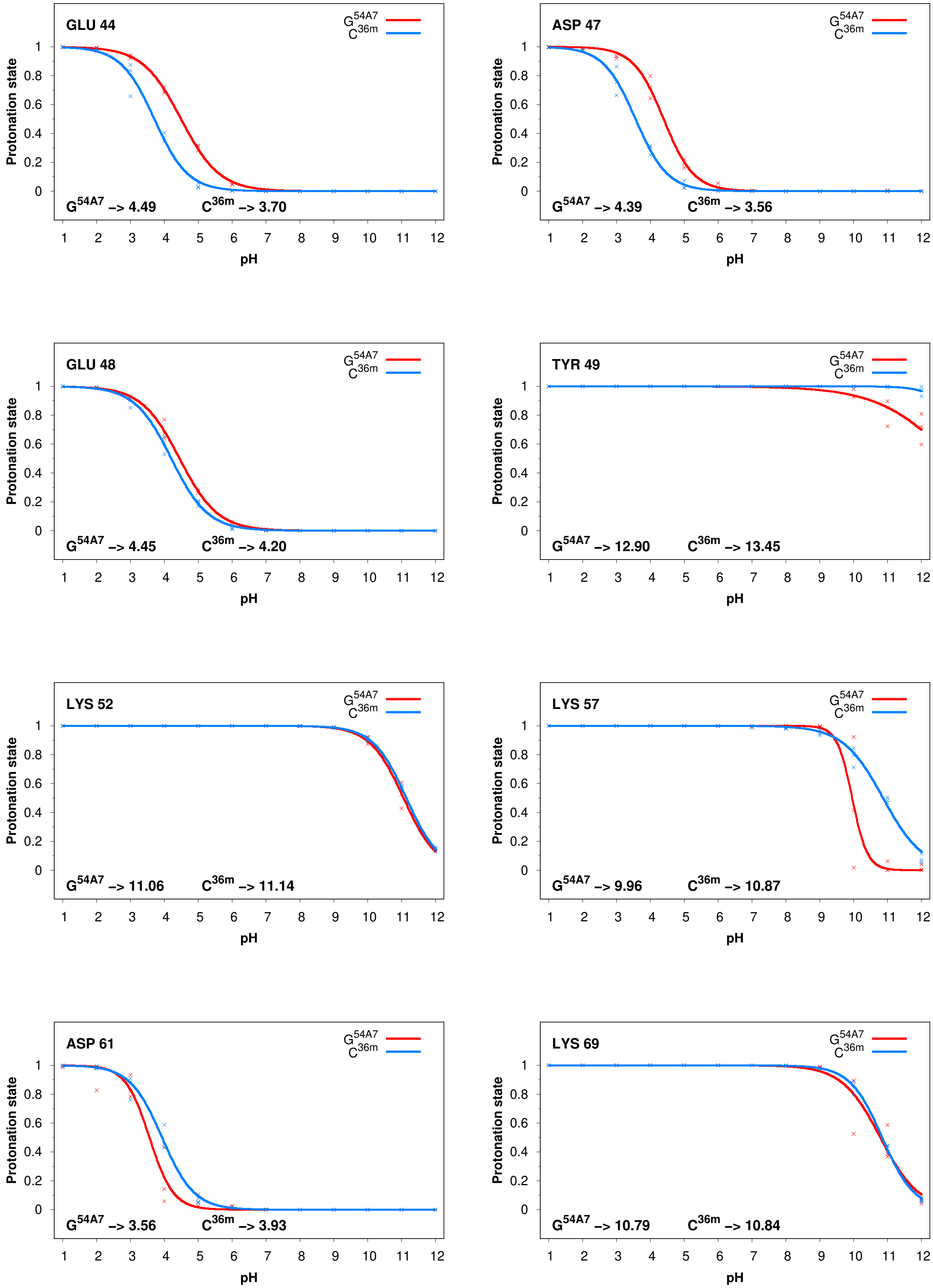
GLU 44
G54A7
C36m
Protonation state
pH
G54A7 –> 4.49
C36m –> 3.70
ASP 47
G54A7 –> 4.39
C36m –> 3.56
GLU 48
G54A7 –> 4.45
C36m –> 4.20
TYR 49
G54A7 –> 12.90
C36m –> 13.45
LYS 52
G54A7 –> 11.06
C36m –> 11.14
LYS 57
G54A7 –> 9.96
C36m –> 10.87
ASP 61
G54A7 –> 3.56
C36m –> 3.93
LYS 69
G54A7 –> 10.79
C36m –> 10.84

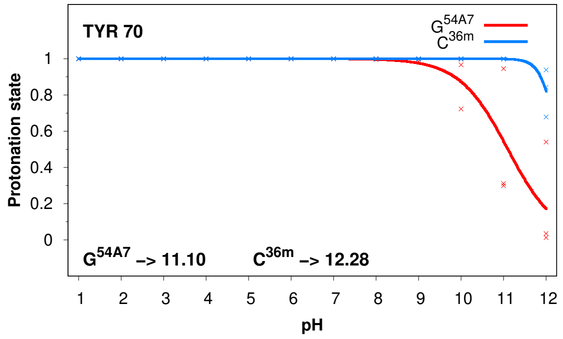
TYR 70
G^54A7
C^36m
Protonation state
pH
G^54A7 –> 11.10
C^36m –> 12.28

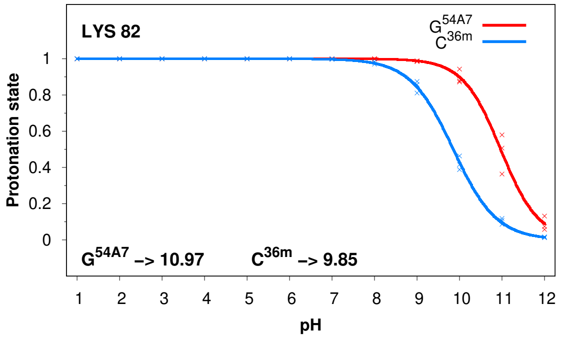
LYS 82
G^54A7
C^36m
Protonation state
pH
G^54A7 –> 10.97
C^36m –> 9.85

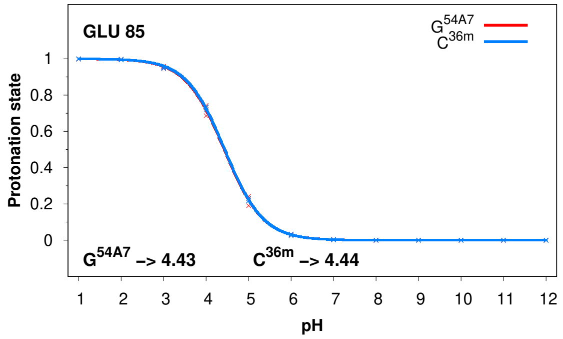
GLU 85
G^54A7
C^36m
Protonation state
pH
G^54A7 –> 4.43
C^36m –> 4.44

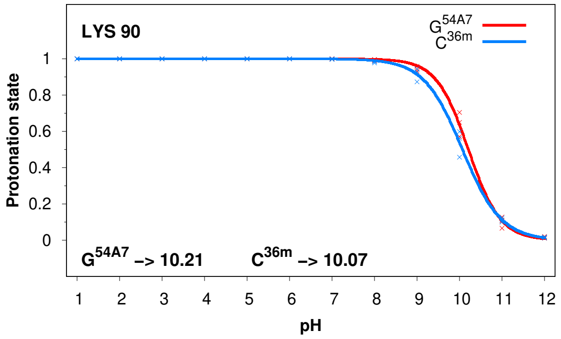
LYS 90
G^54A7
C^36m
Protonation state
pH
G^54A7 –> 10.21
C^36m –> 10.07

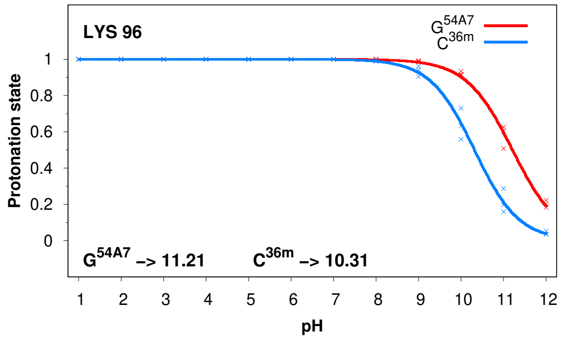
LYS 96
G^54A7
C^36m
Protonation state
pH
G^54A7 –> 11.21
C^36m –> 10.31

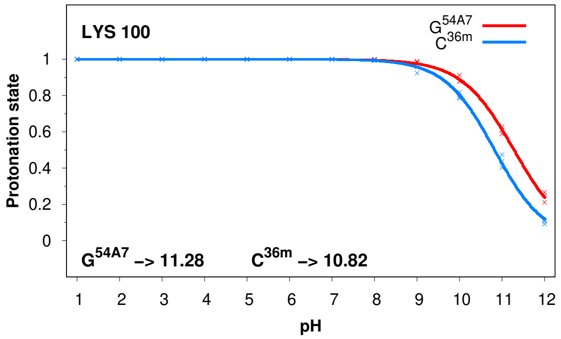
LYS 100
G^54A7
C^36m
Protonation state
pH
G^54A7 –> 11.28
C^36m –> 10.82

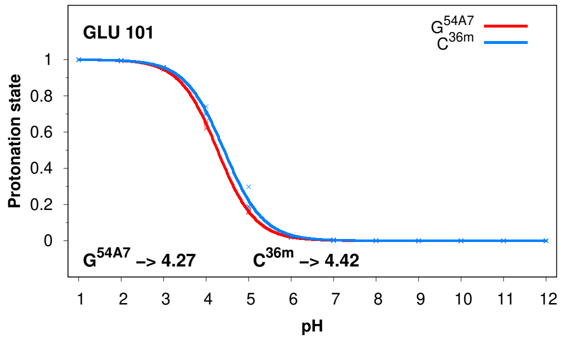
GLU 101
G^54A7
C^36m
Protonation state
pH
G^54A7 –> 4.27
C^36m –> 4.42

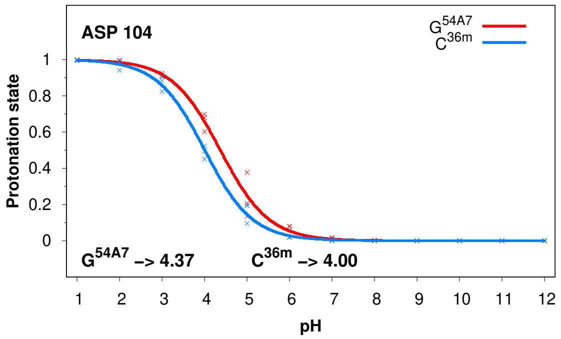
ASP 104
G^54A7
C^36m
Protonation state
pH
G^54A7 –> 4.37
C^36m –> 4.00

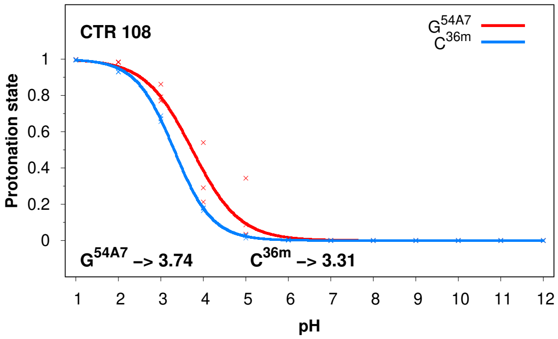

Figure S9: ^{Ec}Trx titration curves calculated for each residue. The average protonation state of each replicate, at each pH value, is represented by the colored dots.

References

(S1) Thurlkill, R. L.; Grimsley, G. R.; Scholtz, J. M.; Pace, C. N. pK values of the ionizable groups of proteins. *Protein Sci.* **2006**, *15*, 1214–1218.

(S2) Grimsley, G. R.; Scholtz, J. M.; Pace, C. N. A summary of the measured pK values of the ionizable groups in folded proteins. *Protein Sci.* **2009**, *18*, 247–251.

(S3) Reis, P. B.; Vila-Viçosa, D.; Rocchia, W.; Machuqueiro, M. PypKa: A Flexible Python Module for Poisson–Boltzmann-Based p K a Calculations. *Journal of Chemical Information and Modeling* **2020**, *60*, 4442–4448.

(S4) Tanford, C.; Roxby, R. Interpretation of protein titration curves. Application to lysozyme. *Biochemistry* **1972**, *11*, 2192–2198.

(S5) Visser, A.; van Engelen, J.; Visser, N.; van Hoek, A.; Hilhorst, R.; Freedman, R. Fluorescence dynamics of staphylococcal nuclease in aqueous solution and reversed micelles. *Biochimica et Biophysica Acta (BBA) - Protein Structure and Molecular Enzymology* **1994**, *1204*, 225–234.

(S6) Wollman, E.; d'Auriol, L.; Rimsky, L.; Shaw, A.; Jacquot, J.; Wingfield, P.; Graber, P.; Dessarps, F.; Robin, P.; Galibert, F. Cloning and expression of a cDNA for human thioredoxin. *Journal of Biological Chemistry* **1988**, *263*, 15506–15512.

(S7) Stefanková, P.; Barák, I. Thioredoxin - structural and functional complexity. *General physiology and biophysics* **2005**, *24*, 3–11.

(S8) Pahari, S.; Sun, L.; Alexov, E. PKAD: a database of experimentally measured pKa values of ionizable groups in proteins. *Database* **2019**, *2019*.

(S9) Machuqueiro, M.; Baptista, A. M. Is the prediction of pK_a values by constant-pH molecular dynamics being hindered by inherited problems? *Proteins Struct. Funct. Bioinf.* **2011**, *79*, 3437–3447.

(S10) Vila-Viçosa, D.; Reis, P. B. P. S.; Baptista, A. M.; Oostenbrink, C.; Machuqueiro, M. A pH Replica Exchange Scheme in the Stochastic Titration Constant-pH MD Method. *J. Chem. Theory Comput.* **2019**, *15*, 3108–3116.

(S11) Swails, J. M.; Roitberg, A. E. Enhancing conformation and protonation state sampling of hen egg white lysozyme using pH replica exchange molecular dynamics. *J. Chem. Theory Comput.* **2012**, *8*, 4393–4404.

(S12) Huang, Y.; Harris, R. C.; Shen, J. Generalized Born based continuous constant pH molecular dynamics in Amber: Implementation, benchmarking and analysis. *J. Chem. Inf. Model.* **2018**, *58*, 1372–1383.

(S13) Huang, Y.; Chen, W.; Wallace, J. A.; Shen, J. All-atom continuous constant pH molecular dynamics with particle mesh Ewald and titratable water. *J. Chem. Theory Comput.* **2016**, *12*, 5411–5421.

(S14) Goh, G. B.; Hulbert, B. S.; Zhou, H.; Brooks III, C. L. Constant pH molecular dynamics of proteins in explicit solvent with proton tautomerism. *Proteins Struct. Funct. Bioinf.* **2014**, *82*, 1319–1331.

(S15) Radak, B. K.; Chipot, C.; Suh, D.; Jo, S.; Jiang, W.; Phillips, J. C.; Schulten, K.; Roux, B. Constant-pH Molecular Dynamics Simulations for Large Biomolecular Systems. *J. Chem. Theory Comput.* **2017**, *13*, 5933–5934.
